# Transcript expression-aware annotation improves rare variant interpretation

**DOI:** 10.1038/s41586-020-2329-2

**Published:** 2020-05-27

**Authors:** Beryl B. Cummings, Konrad J. Karczewski, Jack A. Kosmicki, Eleanor G. Seaby, Nicholas A. Watts, Moriel Singer-Berk, Jonathan M. Mudge, Juha Karjalainen, F. Kyle Satterstrom, Anne H. O’Donnell-Luria, Timothy Poterba, Cotton Seed, Matthew Solomonson, Jessica Alföldi, Genome Aggregation Database Production Team, Jessica Alföldi, Irina M. Armean, Eric Banks, Louis Bergelson, Kristian Cibulskis, Ryan L. Collins, Kristen M. Connolly, Miguel Covarrubias, Beryl B. Cummings, Mark J. Daly, Stacey Donnelly, Yossi Farjoun, Steven Ferriera, Laurent Francioli, Stacey Gabriel, Laura D. Gauthier, Jeff Gentry, Namrata Gupta, Thibault Jeandet, Diane Kaplan, Konrad J. Karczewski, Kristen M. Laricchia, Christopher Llanwarne, Eric V. Minikel, Ruchi Munshi, Benjamin M. Neale, Sam Novod, Anne H. O’Donnell-Luria, Nikelle Petrillo, Timothy Poterba, David Roazen, Valentin Ruano-Rubio, Andrea Saltzman, Kaitlin E. Samocha, Molly Schleicher, Cotton Seed, Matthew Solomonson, Jose Soto, Grace Tiao, Kathleen Tibbetts, Charlotte Tolonen, Christopher Vittal, Gordon Wade, Arcturus Wang, Qingbo Wang, James S. Ware, Nicholas A. Watts, Ben Weisburd, Nicola Whiffin, Genome Aggregation Database Consortium, Carlos A. Aguilar Salinas, Tariq Ahmad, Christine M. Albert, Diego Ardissino, Gil Atzmon, John Barnard, Laurent Beaugerie, Emelia J. Benjamin, Michael Boehnke, Lori L. Bonnycastle, Erwin P. Bottinger, Donald W. Bowden, Matthew J. Bown, John C. Chambers, Juliana C. Chan, Daniel Chasman, Judy Cho, Mina K. Chung, Bruce Cohen, Adolfo Correa, Dana Dabelea, Mark J. Daly, Dawood Darbar, Ravindranath Duggirala, Josée Dupuis, Patrick T. Ellinor, Roberto Elosua, Jeanette Erdmann, Tõnu Esko, Martti Färkkilä, Jose Florez, Andre Franke, Gad Getz, Benjamin Glaser, Stephen J. Glatt, David Goldstein, Clicerio Gonzalez, Leif Groop, Christopher Haiman, Craig Hanis, Matthew Harms, Mikko Hiltunen, Matti M. Holi, Christina M. Hultman, Mikko Kallela, Jaakko Kaprio, Sekar Kathiresan, Bong-Jo Kim, Young Jin Kim, George Kirov, Jaspal Kooner, Seppo Koskinen, Harlan M. Krumholz, Subra Kugathasan, Soo Heon Kwak, Markku Laakso, Terho Lehtimäki, Ruth J. F. Loos, Steven A. Lubitz, Ronald C. W. Ma, Daniel G. MacArthur, Jaume Marrugat, Kari M. Mattila, Steven McCarroll, Mark I. McCarthy, Dermot McGovern, Ruth McPherson, James B. Meigs, Olle Melander, Andres Metspalu, Benjamin M. Neale, Peter M. Nilsson, Michael C. O’Donovan, Dost Ongur, Lorena Orozco, Michael J. Owen, Colin N. A. Palmer, Aarno Palotie, Kyong Soo Park, Carlos Pato, Ann E. Pulver, Nazneen Rahman, Anne M. Remes, John D. Rioux, Samuli Ripatti, Dan M. Roden, Danish Saleheen, Veikko Salomaa, Nilesh J. Samani, Jeremiah Scharf, Heribert Schunkert, Moore B. Shoemaker, Pamela Sklar, Hilkka Soininen, Harry Sokol, Tim Spector, Patrick F. Sullivan, Jaana Suvisaari, E. Shyong Tai, Yik Ying Teo, Tuomi Tiinamaija, Ming Tsuang, Dan Turner, Teresa Tusie-Luna, Erkki Vartiainen, Marquis P. Vawter, James S. Ware, Hugh Watkins, Rinse K. Weersma, Maija Wessman, James G. Wilson, Ramnik J. Xavier, Mark J. Daly, Daniel G. MacArthur

**Affiliations:** 1 Program in Medical and Population Genetics, Broad Institute of MIT and Harvard, Cambridge, MA USA; 2 Analytical and Translational Genetics Unit, Massachusetts General Hospital, Boston, MA USA; 3 Program in Biological and Biomedical Sciences, Harvard Medical School, Boston, MA USA; 4 Program in Bioinformatics and Integrative Genomics, Harvard Medical School, Boston, MA USA; 5 Genomic Informatics Group, University Hospital Southampton, Southampton, UK; 6 European Molecular Biology Laboratory, European Bioinformatics Institute, Wellcome Genome Campus, Hinxton, Cambridge UK; 7 Stanley Center for Psychiatric Research, Broad Institute of MIT and Harvard, Cambridge, MA USA; 8 Division of Genetics and Genomics, Boston Children’s Hospital, Boston, MA USA; 9 Department of Pediatrics, Harvard Medical School, Boston, MA USA; 146 Present Address: Centre for Population Genomics, Garvan Institute of Medical Research, and UNSW Sydney, Syndney, Australia; 147 Present Address: Centre for Population Genomics, Murdoch Children’s Research Institute, Melbourne, Australia; 10 Data Sciences Platform, Broad Institute of MIT and Harvard, Cambridge, MA USA; 11 Center for Genomic Medicine, Massachusetts General Hospital, Boston, MA USA; 12 Genomics Platform, Broad Institute of MIT and Harvard, Cambridge, MA USA; 13 Broad Genomics, Broad Institute of MIT and Harvard, Cambridge, MA USA; 14 Wellcome Sanger Institute, Hinxton, Cambridgeshire UK; 15 National Heart & Lung Institute and MRC London Institute of Medical Sciences, Imperial College London, London, UK; 16 Cardiovascular Research Centre, Royal Brompton & Harefield Hospitals NHS Trust, London, UK; 17 Unidad de Investigacion de Enfermedades Metabolicas, Instituto Nacional de Ciencias Medicas y Nutricion, Mexico City, Mexico; 18 Peninsula College of Medicine and Dentistry, Exeter, UK; 19 Division of Preventive Medicine, Brigham and Women’s Hospital and Harvard Medical School, Boston, MA USA; 20 Division of Cardiovascular Medicine, Brigham and Women’s Hospital and Harvard Medical School, Boston, MA USA; 21 Department of Cardiology, University Hospital, Parma, Italy; 22 Department of Biology, Faculty of Natural Sciences, University of Haifa, Haifa, Israel; 23 Department of Medicine, Albert Einstein College of Medicine, Bronx, NY USA; 24 Department of Genetics, Albert Einstein College of Medicine, Bronx, NY USA; 25 Department of Quantitative Health Sciences, Lerner Research Institute, Cleveland Clinic, Cleveland, OH USA; 26 Sorbonne Université, APHP, Gastroenterology Department, Saint Antoine Hospital, Paris, France; 27 Framingham Heart Study, National Heart, Lung, & Blood Institute and Boston University, Framingham, MA USA; 28 Department of Medicine, Boston University School of Medicine, Boston, MA USA; 29 Department of Epidemiology, Boston University School of Public Health, Boston, MA USA; 30 Department of Biostatistics, Center for Statistical Genetics, University of Michigan, Ann Arbor, MI USA; 31 National Human Genome Research Institute, National Institutes of Health, Bethesda, MD USA; 32 The Charles Bronfman Institute for Personalized Medicine, Icahn School of Medicine at Mount Sinai, New York, NY USA; 33 Department of Biochemistry, Wake Forest School of Medicine, Winston-Salem, NC USA; 34 Center for Genomics and Personalized Medicine Research, Wake Forest School of Medicine, Winston-Salem, NC USA; 35 Center for Diabetes Research, Wake Forest School of Medicine, Winston-Salem, NC USA; 36 Department of Cardiovascular Sciences and NIHR Leicester Biomedical Research Centre, University of Leicester, Leicester, UK; 37 NIHR Leicester Biomedical Research Centre, Glenfield Hospital, Leicester, UK; 38 Department of Epidemiology and Biostatistics, Imperial College London, London, UK; 39 Department of Cardiology, Ealing Hospital NHS Trust, Southall, UK; 40 Imperial College Healthcare NHS Trust, Imperial College London, London, UK; 41 Department of Medicine and Therapeutics, The Chinese University of Hong Kong, Hong Kong, Hong Kong; 42 Program for Neuropsychiatric Research, McLean Hospital, Belmont, MA USA; 43 Department of Psychiatry, Harvard Medical School, Boston, MA USA; 44 Department of Medicine, University of Mississippi Medical Center, Jackson, MI USA; 45 Department of Epidemiology, Colorado School of Public Health, Aurora, CP USA; 46 Department of Medicine and Pharmacology, University of Illinois at Chicago, Chicago, IL USA; 47 Department of Genetics, Texas Biomedical Research Institute, San Antonio, TX USA; 48 Department of Biostatistics, Boston University School of Public Health, Boston, MA USA; 49 Cardiac Arrhythmia Service and Cardiovascular Research Center, Massachusetts General Hospital, Boston, MA USA; 50 Cardiovascular Epidemiology and Genetics, Hospital del Mar Medical Research Institute (IMIM), Barcelona, Spain; 51 Centro de Investigación Biomédica en Red Enfermedades Cardiovaculares (CIBERCV), Barcelona, Spain; 52 Department of Medicine, Medical School, University of Vic-Central University of Catalonia, Vic, Spain; 53 Institute for Cardiogenetics, University of Lübeck, Lübeck, Germany; 54 DZHK (German Research Centre for Cardiovascular Research), partner site Hamburg/Lübeck/Kiel, Lübeck, Germany; 55 University Heart Center Lübeck, Lübeck, Germany; 56 Estonian Genome Center, Institute of Genomics, University of Tartu, Tartu, Estonia; 57 Clinic of Gastroenterology, Helsinki University and Helsinki University Hospital, Helsinki, Finland; 58 Institute of Clinical Molecular Biology (IKMB), Christian-Albrechts-University of Kiel, Kiel, Germany; 59 Cancer Genome Computational Analysis Group, Broad Institute of MIT and Harvard, Cambridge, MA USA; 60 Endocrinology and Metabolism Department, Hadassah-Hebrew University Medical Center, Jerusalem, Israel; 61 Department of Psychiatry and Behavioral Sciences, SUNY Upstate Medical University, Syracuse, NY USA; 62 Institute for Genomic Medicine, Columbia University Medical Center, Hammer Health Sciences, New York, NY USA; 63 Department of Genetics & Development, Columbia University Medical Center, Hammer Health Sciences, New York, NY USA; 64 Centro de Investigacion en Salud Poblacional, Instituto Nacional de Salud Publica, Cuernavaca, Mexico; 65 Genomics, Diabetes and Endocrinology, Lund University, Lund, Sweden; 66 Lund University Diabetes Centre, Malmö, Sweden; 67 Human Genetics Center, University of Texas Health Science Center at Houston, Houston, TX USA; 68 Department of Neurology, Columbia University, New York, NY USA; 69 Institute of Genomic Medicine, Columbia University, New York, NY USA; 70 Institute of Biomedicine, University of Eastern Finland, Kuopio, Finland; 71 Department of Psychiatry, Helsinki University Central Hospital, Lapinlahdentie, Helsinki, Finland; 72 Department of Medical Epidemiology and Biostatistics, Karolinska Institutet, Stockholm, Sweden; 73 Department of Neurology, Helsinki University Central Hospital, Helsinki, Finland; 74 Institute for Molecular Medicine FIMM, University of Helsinki, Helsinki, Finland; 75 Department of Public Health, University of Helsinki, Helsinki, Finland; 76 Department of Medicine, Harvard Medical School, Boston, MA USA; 77 Center for Genome Science, Korea National Institute of Health, Chungcheongbuk-do, South Korea; 78 MRC Centre for Neuropsychiatric Genetics & Genomics, Cardiff University School of Medicine, Cardiff, UK; 79 National Heart and Lung Institute and MRC London Institute of Medical Sciences, Imperial College London, London, UK; 80 Department of Health, THL-National Institute for Health and Welfare, Helsinki, Finland; 81 Section of Cardiovascular Medicine, Department of Internal Medicine, Yale School of Medicine, New Haven, CT USA; 82 Division of Pediatric Gastroenterology, Emory University School of Medicine, Atlanta, Georgia USA; 83 Department of Internal Medicine, Seoul National University Hospital, Seoul, South Korea; 84 The University of Eastern Finland, Institute of Clinical Medicine, Kuopio, Finland; 85 Kuopio University Hospital, Kuopio, Finland; 86 Department of Clinical Chemistry, Fimlab Laboratories and Finnish Cardiovascular Research Center-Tampere, Faculty of Medicine and Health Technology, Tampere University, Tampere, Finland; 87 The Mindich Child Health and Development Institute, Icahn School of Medicine at Mount Sinai, New York, NY USA; 88 Cardiac Arrhythmia Service, Massachusetts General Hospital, Boston, MA USA; 89 Department of Medicine and Therapeutics, The Chinese University of Hong Kong, Hong Kong, China; 90 Li Ka Shing Institute of Health Sciences, The Chinese University of Hong Kong, Hong Kong, China; 91 Hong Kong Institute of Diabetes and Obesity, The Chinese University of Hong Kong, Hong Kong, China; 92 Cardiovascular Research REGICOR Group, Hospital del Mar Medical Research Institute (IMIM), Barcelona, Spain; 93 Department of Genetics, Harvard Medical School, Boston, MA USA; 94 Oxford Centre for Diabetes, Endocrinology and Metabolism, University of Oxford, Churchill Hospital, Oxford, UK; 95 Wellcome Centre for Human Genetics, University of Oxford, Oxford, UK; 96 Oxford NIHR Biomedical Research Centre, Oxford University Hospitals NHS Foundation Trust, John Radcliffe Hospital, Oxford, UK; 97 F Widjaja Foundation Inflammatory Bowel and Immunobiology Research Institute, Cedars-Sinai Medical Center, Los Angeles, CA USA; 98 Atherogenomics Laboratory, University of Ottawa Heart Institute, Ottawa, Canada; 99 Division of General Internal Medicine, Massachusetts General Hospital, Boston, MA USA; 100 Department of Clinical Sciences, University Hospital Malmo Clinical Research Center, Lund University, Malmo, Sweden; 101 Lund University, Dept. Clinical Sciences, Skane University Hospital, Malmo, Sweden; 102 Instituto Nacional de Medicina Genómica (INMEGEN), Mexico City, Mexico; 103 Medical Research Institute, Ninewells Hospital and Medical School, University of Dundee, Dundee, UK; 104 Department of Molecular Medicine and Biopharmaceutical Sciences, Graduate School of Convergence Science and Technology, Seoul National University, Seoul, South Korea; 105 Department of Psychiatry, Keck School of Medicine at the University of Southern California, Los Angeles, CA USA; 106 Department of Psychiatry and Behavioral Sciences, Johns Hopkins University School of Medicine, Baltimore, MD USA; 107 Division of Genetics and Epidemiology, Institute of Cancer Research, London, UK; 108 Research Unit of Clinical Neuroscience, University of Oulu, Oulu, Finland; 109 Research Center, Montreal Heart Institute, Montreal, Quebec Canada; 110 Department of Medicine, Faculty of Medicine, Université de Montréal, Montreal, Quebec Canada; 111 Department of Public Health, Faculty of Medicine, University of Helsinki, Helsinki, Finland; 112 Department of Biomedical Informatics, Vanderbilt University Medical Center, Nashville, TN USA; 113 Department of Medicine, Vanderbilt University Medical Center, Nashville, TN USA; 114 Department of Biostatistics and Epidemiology, Perelman School of Medicine at the University of Pennsylvania, Philadelphia, PA USA; 115 Department of Medicine, Perelman School of Medicine at the University of Pennsylvania, Philadelphia, PA USA; 116 Center for Non-Communicable Diseases, Karachi, Pakistan; 117 National Institute for Health and Welfare, Helsinki, Finland; 118 Deutsches Herzzentrum München, Munich, Germany; 119 Technische Universität München, Munich, Germany; 120 Division of Cardiovascular Medicine, Nashville VA Medical Center and Vanderbilt University, School of Medicine, Nashville, TN USA; 121 Department of Psychiatry, Icahn School of Medicine at Mount Sinai, New York, NY USA; 122 Department of Genetics and Genomic Sciences, Icahn School of Medicine at Mount Sinai, New York, NY USA; 123 Institute for Genomics and Multiscale Biology, Icahn School of Medicine at Mount Sinai, New York, NY USA; 124 Institute of Clinical Medicine Neurology, University of Eastern Finland, Kuopio, Finland; 125 Department of Twin Research and Genetic Epidemiology, King’s College London, London, UK; 126 Departments of Genetics and Psychiatry, University of North Carolina, Chapel Hill, NC USA; 127 Saw Swee Hock School of Public Health, National University of Singapore, National University Health System, Singapore, Singapore; 128 Department of Medicine, Yong Loo Lin School of Medicine, National University of Singapore, Singapore, Singapore; 129 Duke-NUS Graduate Medical School, Singapore, Singapore; 130 Life Sciences Institute, National University of Singapore, Singapore, Singapore; 131 Department of Statistics and Applied Probability, National University of Singapore, Singapore, Singapore; 132 Folkhälsan Institute of Genetics, Folkhälsan Research Center, Helsinki, Finland; 133 HUCH Abdominal Center, Helsinki University Hospital, Helsinki, Finland; 134 Center for Behavioral Genomics, Department of Psychiatry, University of California, San Diego, CA USA; 135 Institute of Genomic Medicine, University of California, San Diego, CA USA; 136 Juliet Keidan Institute of Pediatric Gastroenterology, Shaare Zedek Medical Center, The Hebrew University of Jerusalem, Jerusalem, Israel; 137 Instituto de Investigaciones Biomédicas UNAM, Mexico City, Mexico; 138 Instituto Nacional de Ciencias Médicas y Nutrición Salvador Zubirán, Mexico City, Mexico; 139 Department of Public Health Solutions, National Institute for Health and Welfare, Helsinki, Finland; 140 Radcliffe Department of Medicine, University of Oxford, Oxford, UK; 141 Department of Gastroenterology and Hepatology, University of Groningen and University Medical Center Groningen, Groningen, The Netherlands; 142 Department of Physiology and Biophysics, University of Mississippi Medical Center, Jackson, MS USA; 143 Program in Infectious Disease and Microbiome, Broad Institute of MIT and Harvard, Cambridge, MA USA; 144 Center for Computational and Integrative Biology, Massachusetts General Hospital, Boston, MA USA; 145 Department of Psychiatry & Human Behavior, University of California Irvine, Irvine, CA USA

**Keywords:** Disease genetics, Medical genomics, Transcriptomics

## Abstract

The acceleration of DNA sequencing in samples from patients and population studies has resulted in extensive catalogues of human genetic variation, but the interpretation of rare genetic variants remains problematic. A notable example of this challenge is the existence of disruptive variants in dosage-sensitive disease genes, even in apparently healthy individuals. Here, by manual curation of putative loss-of-function (pLoF) variants in haploinsufficient disease genes in the Genome Aggregation Database (gnomAD)^1^, we show that one explanation for this paradox involves alternative splicing of mRNA, which allows exons of a gene to be expressed at varying levels across different cell types. Currently, no existing annotation tool systematically incorporates information about exon expression into the interpretation of variants. We develop a transcript-level annotation metric known as the ‘proportion expressed across transcripts’, which quantifies isoform expression for variants. We calculate this metric using 11,706 tissue samples from the Genotype Tissue Expression (GTEx) project^2^ and show that it can differentiate between weakly and highly evolutionarily conserved exons, a proxy for functional importance. We demonstrate that expression-based annotation selectively filters 22.8% of falsely annotated pLoF variants found in haploinsufficient disease genes in gnomAD, while removing less than 4% of high-confidence pathogenic variants in the same genes. Finally, we apply our expression filter to the analysis of de novo variants in patients with autism spectrum disorder and intellectual disability or developmental disorders to show that pLoF variants in weakly expressed regions have similar effect sizes to those of synonymous variants, whereas pLoF variants in highly expressed exons are most strongly enriched among cases. Our annotation is fast, flexible and generalizable, making it possible for any variant file to be annotated with any isoform expression dataset, and will be valuable for the genetic diagnosis of rare diseases, the analysis of rare variant burden in complex disorders, and the curation and prioritization of variants in recall-by-genotype studies.

## Abstract (web summary)

A novel variant annotation metric that quantifies the level of expression of genetic variants across tissues is validated in the Genome Aggregation Database (gnomAD) and is shown to improve rare variant interpretation.

## Main

A primary challenge in the use of genome and exome sequencing to predict human phenotypes is that our capacity to identify genetic variation exceeds our ability to interpret their functional impact^3,4^. One underappreciated source of variability for variant interpretation involves differences in alternative mRNA splicing, which enables exons to be expressed at different levels across tissues. These expression differences mean that variants in different regions of a gene can have different phenotypic outcomes depending on the isoforms they affect. For example, variants that occur in an exon differentially included in two isoforms of *CACNA1C* with diverse patterns of tissue expression result in distinct types of Timothy syndrome^5^. Pathogenic variants in the isoform that exhibits multi-tissue expression result in a multi-system disorder^5–7^, whereas those on the isoform predominantly expressed in the heart result in more severe and specific cardiac defects^8^. In addition, Mendelian disease variants have been found on tissue-specific isoforms^9,10^ and isoform expression levels in *TTN* have been used to show that pLoF variants found in healthy controls occur in exons that are absent from dominantly expressed isoforms, whereas those in patients with dilated cardiomyopathy occur on constitutive exons^11^, emphasizing the utility of exon expression information for variant interpretation.

## Isoform diversity and variant interpretation

**Fig. 1:**
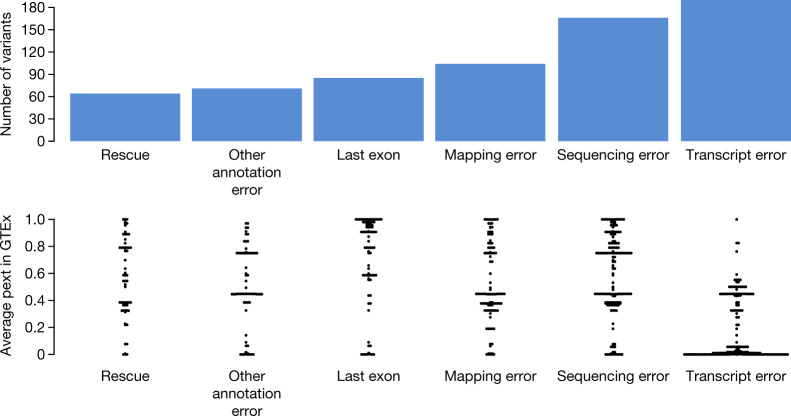
Curation of pLoF variants in haploinsufficient disease genes found in gnomAD reveals transcript errors as a major confounding error mode in variant annotation. We identified and manually curated 401 pLoF variants in the gnomAD dataset in 61 haploinsufficient severe developmental delay genes and flagged any reason the pLoF may not be a true LoF variant. Top, the frequency of each error mode present in the 306 variants classified as unlikely to be a true LoF. Transcript errors emerge as a major putative error mode in the annotation of these pLoF variants. Bottom, bee swarm plot shows the average pext score across GTEx tissues for each variant in the error categories. This shows that pext values are discriminately lower for variants that are annotated as possible transcript errors (*P* = 4.1 × 10^−38^, two-sided Wilcoxon test between transcript errors and other error modes).

We find that isoform diversity is a contributor to the paradoxical finding of disruptive variants in dosage-sensitive disease genes in ostensibly healthy individuals. In the gnomAD database, we identify 401 high-quality pLoF variants that pass both sequencing and annotation quality filters in 61 haploinsufficient disease genes in which heterozygous pLoF variants are established to cause severe developmental delay phenotypes with high penetrance (Methods). Given the severity of these phenotypes and their extremely low prevalence worldwide, ranging from 1 in 10,000 to less than 1 in a million, very few, if any true pLoF variants would be expected to be found in the gnomAD population. As such, most or all of these observed pLoF variants are likely to be sequencing or annotation errors^12^. Manual curation of these variants reveals common error modes that result in probable misannotation of pLoFs, with diversity of transcript structure, mediated by variants falling on low-confidence transcripts, emerging as a major consideration (Fig. 1, Supplementary Fig. 1, Supplementary Tables 1–3). However, no existing tools systematically incorporate information on transcript expression into variant interpretation.

## pext score summarizes isoform expression

**Fig. 2:**
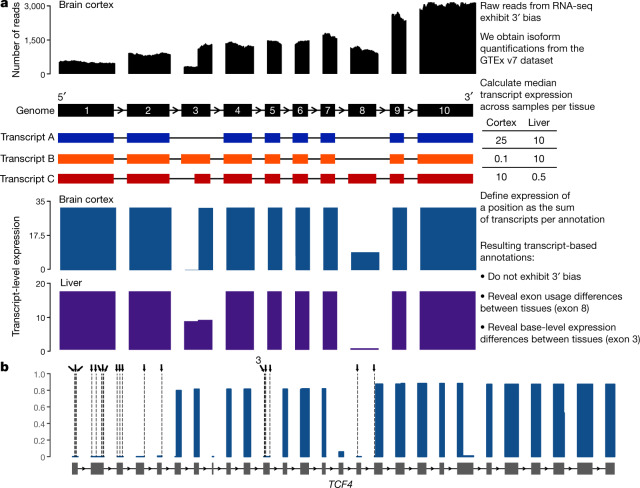
Summary of transcript-expression based annotation method. **a**, Overview of transcript-aware annotation. Most genes have many annotated isoforms, which can have varying expression patterns across tissues. Using the number of reads aligning to exonic regions in transcriptome datasets as a proxy for exon expression (top, black) has confounding effects, due to 3′ bias. In this example, although exons 3 and 8 have markedly different expression levels in brain cortex, the number of reads aligning to the two exons is similar, and this masks the differences in exon usage. Transcript-aware annotation defines the expression of every variant as the sum of transcripts that have the same annotation. The resulting transcript-level expression plots do not exhibit 3′ bias, and reveal differences in exon usage, such as those in exons 3 and 8, across tissues. **b**, Example of utility of transcript-expression based annotation. There are 20 high-quality pLoF variants in the haploinsufficient developmental delay gene *TCF4* in gnomAD, annotated as dashed lines and arrows. All 20 variants have no evidence of expression in the GTEx dataset, which suggests that functional TCF4 protein can be made in the presence of these variants.

The advent of large-scale transcriptome sequencing datasets, such as GTEx^2^, provides an opportunity to incorporate cross-tissue exon expression into variant interpretation. However, the current formats of these databases do not readily allow for unbiased estimation of exon expression. The GTEx web browser offers information on exon-level read pileup across tissues, but this approach is confounded by technical artefacts such as 3′ bias^13^ (preferential coverage of bases close to the 3′ end of a transcript) (Supplementary Fig. 2a). Such systematic biases mean that simple exon-level coverage in a transcriptome dataset cannot be used as a reliable proxy for exon expression, especially in longer genes (Fig. 2a, Supplementary Fig. 2b).

Isoform quantification tools provide estimates of isoform expression levels that correct, albeit imperfectly^13,14^, for confounding by 3′ bias as well as other technical artefacts such as isoform length, isoform GC content, and transcript sequence complexity^15–17^. Here, we use isoform-level quantifications from 11,706 tissue samples from the GTEx v7 dataset to derive an annotation-specific expression metric. For each tissue, we annotate each variant with the expression of every possible consequence across all transcripts, which can be used to summarize expression in any combination of tissues of interest. We first compute the median expression of a transcript across tissue samples, and define the expression of a given variant as the sum of the expression of all transcripts for which the variant has the same annotation (Fig. 2a, Supplementary Fig. 3a). By normalizing the expression of the annotation to the total gene expression, we define a metric (proportion expression across transcripts, or ‘pext’), which can be interpreted as a measure of the proportion of the total transcriptional output from a gene that would be affected by the variant annotation in question (Supplementary Fig. 3b).

The pext metric allows for quick visualization of the expression of exons across a gene. In Fig. 2b, transcript-expression based annotation is shown for *TCF4*, a haploinsufficient gene in which heterozygous variants result in Pitt–Hopkins syndrome^18^, a highly penetrant disorder associated with severe developmental delay. This gene contains 20 unique high-quality pLoF mutations across 56 individuals in the gnomAD database. All 20 variants lie on exons with no evidence of expression across the GTEx dataset (Fig. 2b, Supplementary Fig. 4), which indicates that functional TCF4 protein can be made in the presence of these variants. This visualization is now available for all genes in the gnomAD browser (https://gnomad.broadinstitute.org), and can aid in the rapid identification of variants occurring on exons with little to no evidence of expression in GTEx.

## Functional validation of pext

**Fig. 3:**
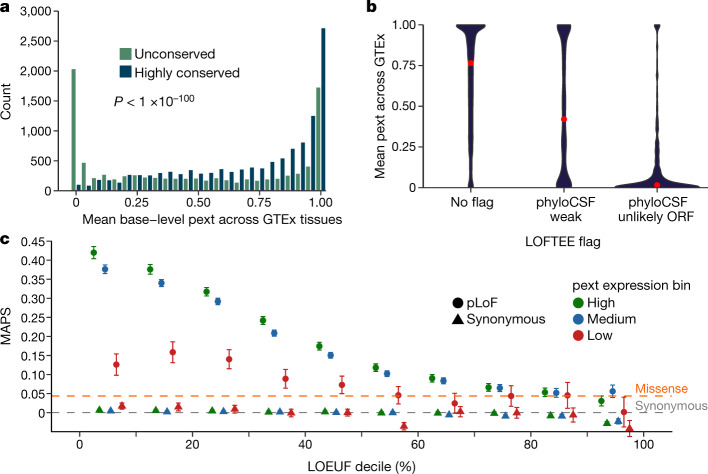
Functional validation of transcript-expression based annotation. **a**, We define highly conserved and unconserved regions as phyloCSF > 1,000 (*n* = 9,817) and phyloCSF < −100 (*n* = 11,860), respectively, and compare the expression status of these regions across GTEx. Regions with high phyloCSF scores are enriched for near-constitutive expression, whereas unconserved regions are enriched for little to no usage across GTEx. This difference is significant after correcting for gene length (logistic regression *P* < 1 × 10^−100^). We note that unconserved regions with high levels of expression (pext > 0.9) are enriched for immune-related genes, which are selected for diversity and thus have low conservation, but represent true coding regions. **b**, Transcript-expression based annotation recapitulates, and adds information to, existing interpretation tools. High-confidence pLoF LOFTEE variants in gnomAD with no flags (*n* = 458,880) are enriched for higher pext values, whereas high-confidence pLoF variants falling on low phyloCSF (*n* = 44,373) or unlikely open-reading frame regions (*n* = 2,437) are enriched for low expression. However, high-confidence pLoF variants can also have a low pext score. Variants flagged falling on regions that are unlikely open-reading frame or have weak conservation are enriched for lower pext values. Red dots denote the median pext value across GTEx, **c**, Non-synonymous variants found on near-constitutive regions tend to be more deleterious. We compared the MAPS score for variants with low (<0.1), medium (0.1 ≤ pext ≤ 0.9) and high (pext > 0.9) expression. Variants with near-constitutive expression have a higher MAPS score, which indicates higher deleteriousness than those with little to no evidence of expression. Points represent MAPS values and error bars denote the 95% confidence interval. Dashed grey and orange lines represent MAPS values for all gnomAD missense and synonymous variants, respectively. The number of variants evaluated per category and unadjusted proportion singleton values can be found in Supplementary Table 5a.

To explore whether expression-based annotation marks functionally important regions, we compared the distribution of the pext metric in evolutionarily conserved and unconserved regions using phyloCSF^19^. Exons with patterns of multi-species conservation consistent with coding regions have higher phyloCSF scores, and should exhibit detectable expression patterns, whereas regions with lower scores will be enriched for incorrect exon annotations, which are expected to have little evidence of expression in a population transcriptome dataset. As expected, we observe significantly lower expression for unconserved regions, and near-constitutive expression in highly conserved regions (Fig. 3a, Supplementary Fig. 5a). This difference remains statistically significant after correcting for exon length (logistic regression *P* < 1.0 × 10^−100^), which can influence both phyloCSF scores and isoform quantifications, indicating that transcript expression-aware annotation marks functionally relevant exonic regions.

Although the metrics are associated, we find that pext provides orthogonal information to conservation for variant interpretation. For example, regions with low evidence of conservation but high expression (Fig. 3a) are enriched for genes in immune-related pathways (Methods), which are selected for diversity but represent true coding regions. In addition, the pext value is higher for pLoF variants annotated as high confidence by the loss-of-function transcript effect estimator (LOFTEE) package^1^, with no additional flags than those flagged as having found on unlikely open-reading frames or weakly conserved regions (Fig. 3b, Supplementary Fig. 5b). However, high-confidence LOFTEE variants with no flags can also have low pext values, which suggests that transcript-expression-aware annotation adds additional information to the currently available interpretation toolkit.

We undertook manual evaluation of 128 regions marked as unexpressed (mean pext < 0.1 in all tissues and in GTEx brain) in 61 haploinsufficient genes following the GENCODE manual annotation workflow^20^ to evaluate the annotation quality in these coding sequence (CDS) regions. One-third of flagged regions were associated with low-quality models that have been removed or switched to non-coding biotypes in subsequent GENCODE releases (Supplementary Fig. 6), and 70% of the remaining regions correspond to models that satisfy only minimum criteria for inclusion in the gene set, corresponding to ‘putative’ annotations that lack markers for CDS functionality (Supplementary Table 4). Nonetheless, we find support for some highly conserved CDS regions, several of which show evidence of transcription in fetal tissues, underlining the importance of incorporating several isoform expression datasets for interpretation (Supplementary Fig. 6d).

Non-synonymous variants found on constitutively expressed regions would be expected to be more deleterious than those on regions with no evidence of expression. To test this, we defined expression bins based on the average pext value across GTEx tissues, in which an average pext value less than 0.1 was defined as low (or unexpressed), above 0.9 as high (or near-constitutive) and intermediate values as medium expression. We compared the mutability-adjusted proportion singleton (MAPS), a measure of negative selection on variant classes^21^, partitioned on the loss-of-function observed/expected upper-bound fraction (LOEUF) decile, a measure of constraint against pLoF variants in the gnomAD dataset^1^ in each of these expression bins. MAPS scores differed substantially between pLoF variants found on low-expressed and high-expressed regions in genes intolerant to pLoF variation (Fig. 3c, Supplementary Fig. 5c, Supplementary Table 5a, b). This information is complementary to existing variant prioritization tools such as PolyPhen-2^22^ (Supplementary Fig. 5d, Supplementary Table 5c). This skew of non-synonymous variation in high-expressed regions suggests that variation arising in such exons tends be more deleterious, whereas non-synonymous variants on regions with low expression are similar to missense variants in their inferred deleteriousness.

## Use of pext in variant interpretation

**Fig. 4:**
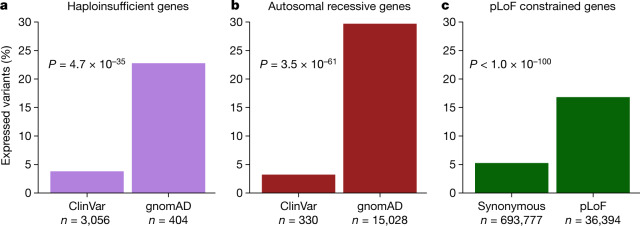
Transcript-expression based annotation aids Mendelian variant interpretation. **a**, Comparison of the proportion of high-quality pLoF variants filtered in a curated list of 61 haploinsufficient developmental delay genes in gnomAD versus ClinVar with a cut-off value of average pext across GTEx ≤ 0.1 (low expression). Expression-based filtering results in removal of 22.8% of gnomAD pLoFs and 3.8% of confidently curated set of pLoFs in ClinVar. **b**, Expression-based annotation filters 30% of pLoF variants found in gnomAD in a homozygous state in at least one individual, and 3.2% of any pLoF variants found in the same genes in ClinVar. **c**, We extended this filtering approach to pLoF and synonymous variants in gnomAD pLoF-intolerant genes (defined by LOEUF < 0.35). This filters 16.8% of LoF and 5.2% of synonymous variants. The total number of high-quality variants considered in each group is shown. For all pLoFs only high-confidence LOFTEE variants were considered. *P* values were determined by two-sided Fisher’s exact test for counts.

To evaluate the utility of transcript expression-based annotation in Mendelian variant interpretation, we assessed the number of variants that would be filtered based on a pext cut-off value of less than 0.1 (low expression) across GTEx tissues for three gene sets. First, we evaluated high-quality pLoF variants in the 61 manually curated haploinsufficient genes in gnomAD and ClinVar^23^. The low pext expression bin resulted in filtering of 22.8% of pLoF variants in haploinsufficient developmental delay genes in gnomAD, but only 3.8% of high-quality pathogenic variants in ClinVar (*P* = 4.7 × 10^−35^) (Fig. 4a, Methods). We next compared pLoF variants in autosomal recessive disease genes found in a homozygous state in at least one individual in gnomAD and any pLoF variant in these genes in ClinVar and observed similar results: expression-based annotation filters 30.0% of variants in gnomAD while only filtering 3.2% of variants in ClinVar (Fig. 4b) (*P* = 3.5 × 10^−61^).

Finally, we evaluated gnomAD pLoF variants in genes that are constrained against pLoF variation^1^ (LOEUF score < 0.35). Given that these genes are depleted for loss-of-function variation in the general population, we expect the observed pLoF variants in these genes to be enriched for annotation errors. We compared the proportion filtered to synonymous variants in the same genes, which we expect to be randomly distributed. Our metric removes 16.8% of pLoF variants in constrained genes, but only 5.2% of synonymous variants (Fig. 4c) (*P* < 1.0 × 10^−100^). In all cases, the vast majority of filtered variants were otherwise high-confidence with no LOFTEE annotation flags, which suggests again that pext provided additional information to existing variant prioritization tools in removing annotation errors (Supplementary Fig. 7).

## Use of pext in burden testing

**Fig. 5:**
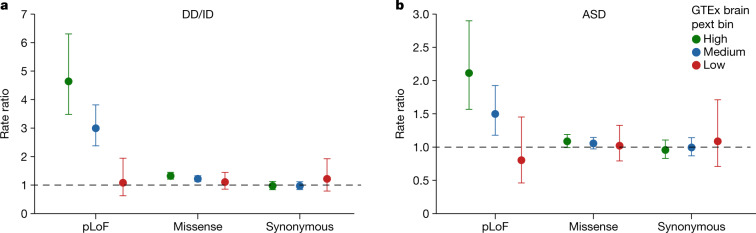
Application of transcript-expression based annotation to de novo variant analyses in ASD and DD/ID. **a**, **b**, Transcript-expression-based analyses in patients with DD/ID (**a**) or ASD (**b**). We find that de novo pLoF variants found on near-constitutively expressed regions in GTEx brain tissues have larger effect sizes than de novo LoF variants in weakly expressed regions in both disorders. Notably, de novo pLoF variants found on regions with little evidence for expression are as equally distributed in cases versus controls as de novo synonymous variants, which suggests that such variants can be removed from analyses of gene burden testing to boost discovery power. The high pext expression bin contains 46.1%, 42.3% and 11.4%, and the low-expression bin contains 4.0%, 6.0% and 11.4% of 1,249, 752 and 166 de novo pLoF variants found in patients with DD/ID, ASD and controls, respectively. Points represent rate ratio estimate and error bars represent 95% confidence interval from the Poisson exact test.

To explore the benefits of this approach for rare variant analysis, we applied pext binning to burden testing of de novo variants in patients with developmental delay/intellectual disability (DD/ID) or autism spectrum disorder (ASD) using a set of 23,970 de novo variants collated from several studies including the Deciphering Developmental Disorders (DDD) project and the Autism Sequencing Consortium (ASC)^24–29^. We find that de novo pLoF variants in patients with DD/ID in low-expressed regions have similar effect sizes to those of synonymous variants (rate ratio of low-expressed pLoFs = 1.08, *P* = 0.90), whereas pLoF variants in highly expressed regions have much larger effect sizes (rate ratio = 4.64, *P* = 3.74 × 10^−38^) (Fig. 5a). This observation is consistent for de novo variants in autism (rate ratio for low-expressed pLoFs = 0.80, *P* = 0.47; rate ratio for high-expressed pLoFs = 2.11, *P* = 8.2 × 10^−8^) (Fig. 5b) and congenital heart disease with co-morbid neurodevelopmental delay (Supplementary Fig. 8a) as well as rare variants (allele count ≤ 10) identified in highly constrained genes in the large iPSYCH case–control study of Danish patients with autism spectrum disorder and attention-deficit/hyperactivity disorder (Supplementary Fig. 8b). Overall, we consistently observe low-expressed pLoFs to have effect sizes similar to those of synonymous variants, with pLoF variants in constitutive regions having larger effect sizes, which suggests that incorporating transcript expression-aware annotation in rare variant studies can boost power for gene discovery.

## Discussion

We have described the development and validation of a transcript expression-based annotation framework to integrate results from transcriptome sequencing experiments into clinical variant interpretation. Although our initial analysis uses GTEx, our method can be used with any isoform expression dataset to annotate any variant file rapidly in the scalable software framework Hail (https://hail.is). For example, annotation of more than 120,000 gnomAD individuals with GTEx takes under an hour using 60 cores, at a cost of about US$5 on public cloud compute, which can be further scaled to larger datasets. In addition, the annotations we provide are flexible: although we have described the use of average transcript-level expression across many tissues, alternative approaches such as using maximum expression across any tissue may prove useful depending on variant interpretation goals (Supplementary Figs. 9, 10).

We note that although this metric successfully discriminates between near-constitutive and low expression levels, which are useful for prioritizing and filtering variants, respectively, regions with intermediate expression levels are more challenging to interpret. However, we hypothesize directed analyses of intermediate expression levels may help to determine the role of alternative splicing in phenotypic diversity^30,31^. In addition, although we have binned average pext scores across GTEx tissues into low, medium and high expression, different genes will probably have varying optimal tissues and thresholds for variant interpretation. Regions tagged as low expression are often corroborated by expert opinion of CDS curation, but domain knowledge of a gene will outperform this summary metric.

An important caveat in our approach is the imprecision of isoform quantification methods using short-read transcriptome data. However, we note that repeating key analyses in the manuscript with a different isoform quantification tool showed consistent results (Methods, Supplementary Fig. 11, Supplementary Table 6), suggesting robustness to the precise pipeline used. The utility of this framework will increase as our ability to quantify isoform expression across tissues improves, including refinement of methods and gene models, as well as availability of long-read RNA-sequencing data from human tissues. In addition, the improvement of single-cell RNA-seq technologies and the generation of data across human tissues will provide insight into cell type-specific exon usage for incorporation into variant interpretation^32^.

The code used to generate pext is available as open source software (https://github.com/macarthur-lab/tx_annotation). In addition, we provide a precomputed file of the transcript expression value for every possible single nucleotide variant in the human genome. This metric has already proven useful in variant curation for the identification of drug targets^33^ and for filtering variants for the identification of human knockouts^1^. Overall, our metric can be incorporated into variant interpretation in Mendelian disease pipelines, analyses of rare variant burden, and the prioritization of variants for recall-by-genotype studies.

## Methods

### Data reporting

No statistical methods were used to predetermine sample size. The experiments were not randomized, and investigators were not blinded to allocation during experiments and outcome assessment.

### Curation of pLoF variants in haploinsufficient developmental disease genes

To identify haploinsufficient developmental delay genes, we selected genes curated by the ClinGen Dosage Sensitivity Working Group^34^ 58 of the 61 genes had a score of 3 with sufficient evidence for pathogenicity, whereas two genes (*CHAMP1*, *CTCF*) had a score of 2 (some evidence) and one gene (*RERE*) was not yet scored. The penetrance of pathogenic variants in each gene was reviewed in the literature, and only genes with more than 75% reported penetrance were included. These conditions are those too severe to expect to see an individual in gnomAD (probably unable to consent for a study without guardianship). The 61 genes include 50 autosomal genes of high severity and high penetrance and 11 genes on chromosome X in which the phenotype is expected to be severe or lethal in males and moderate to severe in females. The resulting gene list is available at gs://gnomad-public/papers/2019-tx-annotation/data/gene_lists/HI_genes_100417.tsv.

We extracted pLoF variants, defined as essential splice acceptor, essential splice donor, stop-gained, and frameshift variants, identified in the 61 haploinsufficient disease genes from the gnomAD v2.1.1 exome and genome sites tables, and considered only those pLoF variants that passed random forest filtering in the gnomAD dataset, and were annotated as high confidence by LOFTEE v1.0. Of 61 genes, 55 had at least one high-quality pLoF available in gnomAD. We performed manual curation of 401 pLoF variants using a web-based curation portal to identify any reason a pLoF may have been a variant calling or annotation error, and categorized the likelihood of each variant being a true LoF.

Evidence for classifying an LoF variant as artefactual was categorized into the following groups: mapping error, strand bias, reference error, genotyping error, homopolymer sequence, in-frame multi-nucleotide variant or frame-restoring indel, essential splice site rescue, minority of transcripts, weak exon conservation, last exon, and other annotation error. All possible reasons also to reject a LoF consequence were flagged, even when a single criterion would categorize the variant as not LoF. Variants were then categorized as LoF, probable LoF, probably not LoF, and not LoF based on criteria outlined in Supplementary Table 2. Supplementary Fig. 1a shows the distribution of the LoF verdicts for the 401 pLoF variants.

Technical errors comprised genotyping errors, strand biases, reference errors, and repetitive regions that could be detected by visual inspection of reads in the Integrative Genomics Viewer^35^ (IGV) and from the UCSC genome browser^36^. Genotyping errors comprised skewed allele balances (conservative cutoff of ≤ 35%), low complexity sequences, GC-rich regions, homopolymer tracts (≥6 base pairs or ≥ 6 trinucleotide repeats) and low quality metrics (genotype quality < 20). Strand bias was flagged when a variant was skewed preferentially on the forward or reverse strand, or when the majority (>90%) of a given strand covered a region; this was often observed around intron–exon boundaries. Strand biases despite balanced coverage of the forward and reverse strands were weighted towards probably not LoF, whereas a strand bias due to skewed strand coverage was weighted alongside other genotyping errors. Reference errors were uncommon, but identified by a small deletion in a given exon, posing as a <5-base-pair intron. Most genotyping errors and strand biases in isolation were not deemed critical in deciding whether a variant was probably not LoF or not LoF, with the exception of allele balance ≤25%. Mapping errors were often identified by an enrichment of complex variation surrounding a variant of interest. Furthermore, the UCSC browser was used to highlight mapping discrepancies, such as self-chain alignments, segmental duplications, simple tandem repeats, and microsatellite regions.

In-frame multi-nucleotide variants (MNVs), essential splice site rescue, and frame-restoring insertion-deletions are rescue events that are predicted to restore gene function. MNVs were visualized in IGV and cross checked with codons from the UCSC browser; in frame MNVs that rescued stop codons were scored as not LoF. Essential splice site rescue occurs when an in frame alternative donor or acceptor site is present, which probably has a minimal effect on the transcript. A total of 36 base pairs upstream and downstream of the splice variant were assessed for splice site rescue. Cryptic splice sites within 6 base pairs of the splice variant were considered a complete rescue, rendering the variant not LoF. Rescue sites >6 base pairs away but within ±20 base pairs were weighted with less confidence, scoring as probably not LoF. All potential splice site rescues were validated using Alamut v.2.11 (https://www.interactive-biosoftware.com/alamut-visual/). Frame-restoring indels were identified by scanning approximately ±80 base pairs from the annotated indel and counting any insertions/deletions to assess if the frame would be restored.

Transcript errors encompass issues surrounding alternative transcripts, variants within a terminal coding exon, poorly conserved exons, and re-initiation events. Coding variants that occupied the minority (<50%) of NCBI coding RefSeq transcripts for a given gene were considered not LoF. These variants often affected poorly conserved exons, as determined by PhyloP^37^, PhyloCSF^19^ and visualization in the UCSC browser^36^. The only exceptions to the minority of transcript criteria were cases where the exon was well conserved, which relegated the categorization to probably not LoF. Variants within the last coding exon, or within 50 base pairs of the penultimate coding exon were also considered not LoF, unless 25% < *x* < 50% of the coding sequence was affected, in which case the variant was deemed probably not LoF. If >50% of the coding sequence was disrupted by a variant in the last exon, this was deemed probably LoF. Other transcript errors included: re-initiation errors; upstream stop codons of a given LoF variant; variants that fell on exactly 50% of coding RefSeq transcripts; and/or partial exon conservation. Re-initiation events were flagged when a methionine downstream of the variant in the first coding exon was predicted to restart transcription, and were predicted to be probably not LoF. Variants occurring after a stop codon in the last coding exon were considered not LoF, particularly across the region of the exon or transcript in question. Error categories were grouped for Fig. 1 as follows: Minority of transcripts and weak exon conservation were grouped as transcript errors, genotyping errors and homopolymers as sequencing errors, essential splice rescue and MNV grouped as rescue and strand bias was included in other annotation errors.

The criteria above were strictly adhered throughout and manual curation was performed by two independent reviewers to ensure maximum consistency and minimize human error. Any discordance in curation was re-curated by both curators together and resolved. Full results of manual curation are available in Supplementary Table 3.

### Calculation of transcript-expression aware annotation

We first imported the GTEx v7 isoform quantifications into Hail and calculated the median expression of every transcript per tissue. This precomputed summary isoform expression matrix is available for GTEx v7 in gs://gnomad-public/papers/2019-tx-annotation/data/GRCH37_hg19/. We also import and annotate a variant file with the Variant Effect Predictor (VEP) version 85^38^ against Gencode v19^20^, implemented in Hail with the LOFTEE v1.0 plugin.

We use the transcript consequences VEP field to calculate the sum of isoform expression for variant annotations, that is, the annotation-level expression across transcripts (ext). For variants that have multiple consequences for one transcript (for example, a single nucleotide variant that is both a missense and a splice region variant on one transcript) we use the worst consequence, ordered by VEP (in this example, missense takes precedence over splice region). We filter the consequences to those only occurring on protein coding transcripts. Full ordering of the VEP consequences is available at: useast.ensembl.org/info/genome/variation/prediction/predicted_data.html

We then sum the expression of every transcript per variant, for every combination of consequence, LOFTEE filter, and LOFTEE flag for every tissue (Supplementary Fig. 3a). For example, if a single nucleotide variant is synonymous on ENST1, a high-confidence LOFTEE stop-gained variant on ENST3 and ENST4, and low-confidence LOFTEE stop-gained variant on ENST5 and ENST6, the ext values will be synonymous: ENST1, stop-gained high-confidence: ENST3 + ENST4, and stop-gained low-confidence: ENST5 + ENST6 per tissue. This can be computed with the tx_annotate() function by setting the tx_annotation_type to ‘expression’. We foresee the non-normalized ext values to be useful when only considering one tissue of interest.

To allow for taking average expression values across tissues of interest, we normalize the expression value for a given value to the total expression of the gene on which the variant is found. This is carried out by dividing the ext value with the sum of the expression of all transcripts per tissue in transcripts per million (TPM) (Supplementary Fig. 3b). The resulting pext value can be interpreted as the proportion of the total transcriptional output from a gene that would be affected by the given variant annotation in question. If the gene expression value (and thus the denominator) in a given tissue is 0, the pext value will not be available (NA) for that tissue.

When taking averages across tissues, such unavailable pext values are not considered (that is, when taking the mean across tissues, we remove NA values). This value can be computed with the tx_annotate() function by setting the tx_annotation_type to ‘proportion’. For the analyses in this manuscript, we remove reproduction-associated GTEx tissues (endocervix, ectocervix, fallopian tube, prostate, uterus, ovary, testes and vagina), cell lines (transformed fibroblasts and transformed lymphocytes) and any tissue with less than 100 samples (bladder, brain Cervicalc-1 spinal cord, brain substantia nigra, kidney cortex and minor salivary gland), resulting in the use of 38 GTEx tissues.

We note that for a minority of genes, when RSEM^15^ assigns higher relative expression to non-coding transcripts, the sum of the value of coding transcripts can be much smaller than the gene expression value for the transcript, resulting in low pext scores for all coding variants in the gene, and thus resulting in possible filtering of all variants for a given gene. In many cases this seems to be the result of spurious non-coding transcripts with a high degree of exon overlap with true coding transcripts. To prevent this artefact from affecting our analyses, we first calculated the maximum pext score for all variants across all protein-coding genes, and removed any gene where the maximum pext score was below 0.2. This resulted in the filtering of 668 genes, representing 3.3% of all genes analysed. We note that there is no overlap with the 668 genes and the haploinsufficient gene list, 97 of the filtered genes are present in OMIM (representing 1.5% of the OMIM gene list) and 42 filtered genes are considered constrained (representing 1.4% of LOEUF <0.35, or constrained, genes) thus having low effect on variant interpretation in the context of disease associations.

The full transcript-expression aware annotation pipeline, implemented in Hail 0.2, is fully available at https://github.com/macarthur-lab/tx_annotation with commands laid out for analyses in the manuscript. Passing a Hail table through the tx_annotate() function returns the same table with a new field entitled ‘tx_annotation’ which provides either the ext or pext value per variant-annotation pair, depending on parameter choice. We provide a helper function to extract the worst consequence and the associated expression values for these annotations. All analyses in the manuscript are based on the worst consequence of variant, ordered by VEP^38^.

### Functional validation of transcript-expression aware annotation

Conservation analysis was performed using phyloCSF scores using the same file used for the LOFTEE plugin, available publicly in gs://gnomad-public/papers/2019-tx-annotation/data/other_data/phylocsf_data.tsv.bgz . We denoted exons with a phyloCSF max open-reading frame score >1,000 as highly conserved and those with phyloCSF max open-reading frame score <−100 as lowly conserved (Supplementary Fig. 5a) and evaluated their average usage in GTEx.

Using the base-level pext values that are used in the gnomAD browser, we filtered to intervals with high or low conservation, and calculated the average pext value in the interval. To evaluate regions with low conservation but high expression, we identified genes harbouring unconserved regions with the pext value >0.9 for pathway enrichment analysis and used the web browser for FUMA GENE2FUNC feature^39^, which incorporates Reactome^40^, KEGG^41^, Gene Ontology^42^ (GO) as well as other ontologies. Default parameters were used for FUMA, with all protein coding genes as the background list. Results from FUMA pathway analysis are available in Supplementary Fig. 12, and full results are available in Supplementary Table 7.

Analysis of pext values for LOFTEE flags and the MAPS calculation were performed using the gnomAD v2.1.1 exome dataset. Calculation of MAPS scores was previously described^21^ and is implemented as a Hail module, as also described previously^1^. MAPS is a relative metric, and cannot be compared across datasets, but is a useful summary metric for the frequency spectrum, indicating deleteriousness as inferred from rarity of variation (high values of MAPS correspond to lower frequency, suggesting the action of negative selection at more deleterious sites). The MAPS scores were calculated on the gnomAD v.2.1.1 dataset partitioning upon the LOEUF score and expression bin. The script for generating MAPs scores is available in the tx-annotation Github repository under /analyses/maps/maps_submit_per_class.py

### Manual evaluation of unexpressed regions in haploinsufficient developmental delay genes using the GENCODE workflow

As an orthogonal evaluation of regions flagged as unexpressed with the pext metric, we identified any region in 61 haploinsufficient disease genes with a mean pext value <0.1 in all GTEx tissues and in GTEx brain samples, owing to the relevance of brain tissues for these disorders, regardless of mutational burden in gnomAD. The resulting list of 128 regions was evaluated by the HAVANA manual annotation group of the GENCODE project^20^.

The manual evaluation first established whether the transcript model corresponding to the region in question was correct in terms of structure, comparing exon–intron combinations, and the accuracy of splice sites against the RNA evidence supporting the model. Second, the functional biotype of each model was reassessed; in particular, whether the decision to annotate the model as protein-coding in GENCODE v19 was appropriate. Note that GENCODE models that incorporate alternative exons or exon combinations in comparison to the ‘canonical’ isoform are likely to be annotated as coding if they contain a prospective CDS that is considered biologically plausible, based on a mechanistic view of translation. These re-annotations are summarized in Supplementary Table 5.

We binned cases into three main categories, according to confidence in both the accuracy and potential functional relevance of the overlapping models: (1) ‘error’, in which the model was seen to have an incorrect transcript structure and/or a CDS that conflicted with updated GENCODE annotation criteria (these annotations had been or will be changed in future GENCODE releases based on this evaluation); (2) ‘putative’, in which the model structure and CDS satisfied our current annotation criteria, although we judged the potential of the transcript represented to encode a protein with a functional role in cellular physiology to be nonetheless speculative (these have been maintained as putative protein-coding transcripts in GENCODE); (3) ‘validated’, in which we believe it is highly probable that the model represents a true protein-coding isoform. High confidence in the validity of the CDS was based on comparative annotation, that is, the observation of CDS conservation and also the existence of equivalent transcript models in other species. GENCODE also annotates transcript models as ‘nonsense-mediated decay’ and ‘non-stop decay’, in which a translation is found that is predicted to direct the RNA molecule into cellular degradation programs. Although it has been established that such ‘non-productive’ transcription events can have a role in gene regulation and thus disease, the interpretation of variants within nonsense-mediated decay and non-stop decay CDS regions remains challenging^43^. These models were therefore classed in a separate category.

### Gene list comparisons

To evaluate the filtering power of the pext metric for Mendelian variants, we evaluated the number of variants that would be filtered with an average GTEx pext cutoff of 0.1 (low expression) in the ClinVar and gnomAD datasets. We downloaded the ClinVar VCF from the ClinVar FTP (version dated 10/28/2018), imported it into Hail, annotated it with VEP v85 against Gencode v19, and added pext annotations with the tx_annotate() function. All evaluated variants were annotated as HC by LOFTEE v1.0, and ClinVar variants were filtered to those marked as pathogenic, with no conflicts, and reviewed with at least one star status.

For variants in 61 haploinsufficient genes, we identified any variant identified in at least one individual with any zygosity in both datasets. For variants identified in autosomal recessive disease genes, we used a list of 1,183 OMIM disease genes deemed to follow a recessive inheritance pattern by Blekhman et al.^44^ and Berg et al.^45^ (available as https://github.com/macarthur-lab/gene_lists/blob/master/lists/all_ar.tsv). We compared the pext value for all pLoF variants identified in ClinVar versus any variant in a homozygous state in at least one individual in the gnomAD exome or genome datasets. Finally, we used a LOEUF cutoff of 0.35 to denote constrained genes, and compared any synonymous or pLoF variant in these genes in the gnomAD exome or genome datasets.

### De novo and rare variant analysis

De novo variants were collated from previously published studies. We collected de novo variants identified in 5,305 probands from trio studies of intellectual disability/developmental disorders (Hamdam et al.^27^: *n* = 41, de Ligt et al.^28^: *N* = 100, Rauch et al.^29^: *N* = 51, DDD^24^: *n* = 4,293, Lelieveld et al.^26^: *n* = 820), 1,073 probands with congenital heart disease with co-morbid developmental delay (Sifrim et al.^46^: *n* = 512, Chih Jin et al.^47^: 561), 6,430 ASD probands, and 2,179 unaffected controls from the Autism Sequencing Consortium^25^. We also used a previously published dataset of variants in 8,437 cases with ASD and/or attention-deficit/hyperactivity disorder and 5,214 controls from the Danish Neonatal Screening Biobank^48^. In this analysis, we analysed pLoF variants identified in highly constrained genes (first LOEUF decile) with a combined total allele count of ≤ 10 in cases and controls.

We annotated both de novo and rare variants with VEP v85 against Gencode v19 and added pext annotations with the tx_annotate() function. We then calculated the average pext metric across 11 GTEx brain samples and binned them as low (pext < 0.1), medium (0.1 ≤ pext ≤ 0.9) or high (pext > 0.9) expression. We then calculated the number of pLoF, missense, and synonymous variants per pext expression bin. To obtain case-control rate ratios and the 95% confidence intervals for de novo variant analyses, we used a two-sided Poisson exact test on counts. To obtain the odds ratio for the rare variant analysis in ASD/ADHD, we used the Fisher’s exact test for count data.

### Isoform quantifications via salmon

To evaluate whether use of a different isoform quantification tool would affect results, we compared results of *TCF4* base-level expression (shown in Fig. 2b), MAPS (Fig. 3c) and comparison of the number of variants filtered in haploinsufficient developmental disease genes in ClinVar vs gnomAD (Fig. 4a) using RSEM quantifications used in this study with quantifications using salmon v.0.12^17^. Due to the intractability of re-quantifying the entire GTEx dataset, we downloaded and requantified 151 GTEx brain cortex CRAM files from the V7 dataset. We first converted CRAMs to fastq files using Picard 2.18.20 and ran salmon with the ‘salmon quant –i index -fastq1 – fastq2 –minAssignedFrag1 –validateMappings’ command. The index was created with the ‘salmon index –t transcript.fa –type quasi –k 31’ command using the GENCODE v19 protein-coding and lncRNA transcripts FASTA files. The existing GTEx RSEM isoform quantifications were filtered to the same GTEx brain cortex samples. For the analyses to remain consistent with the remainder of the manuscript, we calculated the maximum brain cortex pext score for all variants across all protein-coding genes for both the RSEM and salmon quantifications, and removed any gene in which the maximum pext score was below 0.2. This resulted in filtering 325 genes from the salmon quantification of the brain cortex samples and 691 genes from the RSEM quantification, corresponding to 3.4 and 1.6% of quantified genes, respectively. We filtered these genes in both the MAPs and gene list comparison analysis seen in Supplementary Fig. 11. The WDL script for the quantification pipeline is available at: gs://gnomad-public/papers/2019-tx-annotation/results/salmon_rsem/salmon.wdl and the commands to obtain results for each individual analysis in the tx-annotation Github repository under /analyses/rsem_salmon/.

### Transcript expression aware annotation with fetal isoform expression dataset

Although our analyses were based on transcript expression aware annotation from the GTEx v7 dataset, we provide necessary files for pext annotation with the Human Brain Development Resource (HBDR) fetal brain dataset^49^ in gs://gnomad-public/papers/2019-tx-annotation/data/HBDR_fetal_RNaseq. HBDR includes 558 samples from varying brain subregions across developmental time points. We downloaded HDBR sample fastq files from European Nucleotide Archive (study accession PRJEB14594) and obtained RSEM isoform quantification on HBDR fastqs using the GTEx v7 quantification pipeline, publicly available at https://github.com/broadinstitute/gtex-pipeline/) which briefly involves two-pass alignment with STAR v2.4.2a^50^ and isoform quantification with RSEM v1.2.22. Here, we also removed genes where the average pext across HBDR was below 0.2, resulting in the removal of 712 genes (3.5% of all analysed genes). The dataset was also used for the analysis of baselevel expression values in *SCN2A* shown in Supplementary Fig. 7d.

### Reporting summary

Further information on research design is available in the Nature Research Reporting Summary linked to this paper.

## Online content

Any methods, additional references, Nature Research reporting summaries, source data, extended data, supplementary information, acknowledgements, peer review information; details of author contributions and competing interests; and statements of data and code availability are available at 10.1038/s41586-020-2329-2.

## Supplementary information

Supplementary Information This file contains Supplementary Figures 1-3 and 5-12, and Supplementary Tables 1, 2, 5 and 6.

Reporting Summary

Supplementary Figure This file contains Supplementary Figure 4: Baselevel TCF4 expression per GTEx tissue.

Supplementary Table This file contains Supplementary Table 3: Manual curation results of 401 pLoFs in 61 HI developmental disease genes identified in gnomAD.

Supplementary Table This file contains Supplementary Table 4: GENCODE curation results of 128 regions flagged as unexpressed by pext.

Supplementary Table This file contains Supplementary Table 7: FUMA GENE2FUNC analysis results and run information. All statistics were generated by FUMA, described in Ref 40.

Peer Review File Reviewer reports and authors' response from the peer review of this Article at Nature.

## Data Availability

We used the gnomAD v.2.1.1 sites Hail 0.2 (https://hail.is) table that is accessible publicly at gs://gnomad-public/release/2.1.1 and at https://gnomad.broadinstitute.org. The GTEx v7 gene and isoform expression data were downloaded from the GTEx portal (gtexportal.org). The LOEUF constraint file was downloaded from gs://gnomad-resources/lof_paper/. All files used in the analyses in the manuscript are available in gs://gnomad-public/papers/2019-tx-annotation/.

## Code availability

The GTEx pipeline for isoform quantification is available publicly (https://github.com/broadinstitute/gtex-pipeline/) and briefly involves two-pass alignment with STAR v2.4.2a^50^, gene expression quantification with RNA-SeQC v1.1.8^51^, and isoform quantification with RSEM v1.2.22. Variants used in all gnomAD analyses in the manuscript passed random forest filtering, and all pLoF variants were annotated as high-confidence by LOFTEE v.1.0, which is described in an accompanying manuscript^1^. Scripts to quality control the gnomAD dataset are available at https://github.com/macarthur-lab/gnomad_qc and the scripts to generate files for the analyses are available at https://github.com/macarthur-lab/tx_annotation.
