# Author Correction: Transcript expression-aware annotation improves rare variant interpretation

**DOI:** 10.1038/s41586-020-03175-7

**Published:** 2021-02-03

**Authors:** Beryl B. Cummings, Konrad J. Karczewski, Jack A. Kosmicki, Eleanor G. Seaby, Nicholas A. Watts, Moriel Singer-Berk, Jonathan M. Mudge, Juha Karjalainen, F. Kyle Satterstrom, Anne H. O’Donnell-Luria, Timothy Poterba, Cotton Seed, Matthew Solomonson, Jessica Alföldi, Genome Aggregation Database Production Team, Jessica Alföldi, Irina M. Armean, Eric Banks, Louis Bergelson, Kristian Cibulskis, Ryan L. Collins, Kristen M. Connolly, Miguel Covarrubias, Beryl B. Cummings, Mark J. Daly, Stacey Donnelly, Yossi Farjoun, Steven Ferriera, Laurent Francioli, Stacey Gabriel, Laura D. Gauthier, Jeff Gentry, Namrata Gupta, Thibault Jeandet, Diane Kaplan, Konrad J. Karczewski, Kristen M. Laricchia, Christopher Llanwarne, Eric V. Minikel, Ruchi Munshi, Benjamin M. Neale, Sam Novod, Anne H. O’Donnell-Luria, Nikelle Petrillo, Timothy Poterba, David Roazen, Valentin Ruano-Rubio, Andrea Saltzman, Kaitlin E. Samocha, Molly Schleicher, Cotton Seed, Matthew Solomonson, Jose Soto, Grace Tiao, Kathleen Tibbetts, Charlotte Tolonen, Christopher Vittal, Gordon Wade, Arcturus Wang, Qingbo Wang, James S. Ware, Nicholas A. Watts, Ben Weisburd, Nicola Whiffin, Genome Aggregation Database Consortium, Carlos A. Aguilar Salinas, Tariq Ahmad, Christine M. Albert, Diego Ardissino, Gil Atzmon, John Barnard, Laurent Beaugerie, Emelia J. Benjamin, Michael Boehnke, Lori L. Bonnycastle, Erwin P. Bottinger, Donald W. Bowden, Matthew J. Bown, John C. Chambers, Juliana C. Chan, Daniel Chasman, Judy Cho, Mina K. Chung, Bruce Cohen, Adolfo Correa, Dana Dabelea, Mark J. Daly, Dawood Darbar, Ravindranath Duggirala, Josée Dupuis, Patrick T. Ellinor, Roberto Elosua, Jeanette Erdmann, Tõnu Esko, Martti Färkkilä, Jose Florez, Andre Franke, Gad Getz, Benjamin Glaser, Stephen J. Glatt, David Goldstein, Clicerio Gonzalez, Leif Groop, Christopher Haiman, Craig Hanis, Matthew Harms, Mikko Hiltunen, Matti M. Holi, Christina M. Hultman, Mikko Kallela, Jaakko Kaprio, Sekar Kathiresan, Bong-Jo Kim, Young Jin Kim, George Kirov, Jaspal Kooner, Seppo Koskinen, Harlan M. Krumholz, Subra Kugathasan, Soo Heon Kwak, Markku Laakso, Terho Lehtimäki, Ruth J. F. Loos, Steven A. Lubitz, Ronald C. W. Ma, Daniel G. MacArthur, Jaume Marrugat, Kari M. Mattila, Steven McCarroll, Mark I. McCarthy, Dermot McGovern, Ruth McPherson, James B. Meigs, Olle Melander, Andres Metspalu, Benjamin M. Neale, Peter M. Nilsson, Michael C. O’Donovan, Dost Ongur, Lorena Orozco, Michael J. Owen, Colin N. A. Palmer, Aarno Palotie, Kyong Soo Park, Carlos Pato, Ann E. Pulver, Nazneen Rahman, Anne M. Remes, John D. Rioux, Samuli Ripatti, Dan M. Roden, Danish Saleheen, Veikko Salomaa, Nilesh J. Samani, Jeremiah Scharf, Heribert Schunkert, Moore B. Shoemaker, Pamela Sklar, Hilkka Soininen, Harry Sokol, Tim Spector, Patrick F. Sullivan, Jaana Suvisaari, E. Shyong Tai, Yik Ying Teo, Tuomi Tiinamaija, Ming Tsuang, Dan Turner, Teresa Tusie-Luna, Erkki Vartiainen, Marquis P. Vawter, James S. Ware, Hugh Watkins, Rinse K. Weersma, Maija Wessman, James G. Wilson, Ramnik J. Xavier, Mark J. Daly, Daniel G. MacArthur

**Affiliations:** 1 Program in Medical and Population Genetics, Broad Institute of MIT and Harvard, Cambridge, MA USA; 2 Analytical and Translational Genetics Unit, Massachusetts General Hospital, Boston, MA USA; 3 Program in Biological and Biomedical Sciences, Harvard Medical School, Boston, MA USA; 4 Program in Bioinformatics and Integrative Genomics, Harvard Medical School, Boston, MA USA; 5 Genomic Informatics Group, University Hospital Southampton, Southampton, UK; 6 European Molecular Biology Laboratory, European Bioinformatics Institute, Wellcome Genome Campus, Hinxton, Cambridge UK; 7 Stanley Center for Psychiatric Research, Broad Institute of MIT and Harvard, Cambridge, MA USA; 8 Division of Genetics and Genomics, Boston Children’s Hospital, Boston, MA USA; 9 Department of Pediatrics, Harvard Medical School, Boston, MA USA; 146 Present Address: Centre for Population Genomics, Garvan Institute of Medical Research, and UNSW Sydney, Syndney, Australia; 147 Present Address: Centre for Population Genomics, Murdoch Children’s Research Institute, Melbourne, Australia; 10 Data Sciences Platform, Broad Institute of MIT and Harvard, Cambridge, MA USA; 11 Center for Genomic Medicine, Massachusetts General Hospital, Boston, MA USA; 12 Genomics Platform, Broad Institute of MIT and Harvard, Cambridge, MA USA; 13 Broad Genomics, Broad Institute of MIT and Harvard, Cambridge, MA USA; 14 Wellcome Sanger Institute, Hinxton, Cambridgeshire UK; 15 National Heart & Lung Institute and MRC London Institute of Medical Sciences, Imperial College London, London, UK; 16 Cardiovascular Research Centre, Royal Brompton & Harefield Hospitals NHS Trust, London, UK; 17 Unidad de Investigacion de Enfermedades Metabolicas, Instituto Nacional de Ciencias Medicas y Nutricion, Mexico City, Mexico; 18 Peninsula College of Medicine and Dentistry, Exeter, UK; 19 Division of Preventive Medicine, Brigham and Women’s Hospital and Harvard Medical School, Boston, MA USA; 20 Division of Cardiovascular Medicine, Brigham and Women’s Hospital and Harvard Medical School, Boston, MA USA; 21 Department of Cardiology, University Hospital, Parma, Italy; 22 Department of Biology, Faculty of Natural Sciences, University of Haifa, Haifa, Israel; 23 Department of Medicine, Albert Einstein College of Medicine, Bronx, NY USA; 24 Department of Genetics, Albert Einstein College of Medicine, Bronx, NY USA; 25 Department of Quantitative Health Sciences, Lerner Research Institute, Cleveland Clinic, Cleveland, OH USA; 26 Sorbonne Université, APHP, Gastroenterology Department, Saint Antoine Hospital, Paris, France; 27 Framingham Heart Study, National Heart, Lung, & Blood Institute and Boston University, Framingham, MA USA; 28 Department of Medicine, Boston University School of Medicine, Boston, MA USA; 29 Department of Epidemiology, Boston University School of Public Health, Boston, MA USA; 30 Department of Biostatistics, Center for Statistical Genetics, University of Michigan, Ann Arbor, MI USA; 31 National Human Genome Research Institute, National Institutes of Health, Bethesda, MD USA; 32 The Charles Bronfman Institute for Personalized Medicine, Icahn School of Medicine at Mount Sinai, New York, NY USA; 33 Department of Biochemistry, Wake Forest School of Medicine, Winston-Salem, NC USA; 34 Center for Genomics and Personalized Medicine Research, Wake Forest School of Medicine, Winston-Salem, NC USA; 35 Center for Diabetes Research, Wake Forest School of Medicine, Winston-Salem, NC USA; 36 Department of Cardiovascular Sciences University of Leicester, Leicester, UK; 37 NIHR Leicester Biomedical Research Centre, Glenfield Hospital, Leicester, UK; 38 Department of Epidemiology and Biostatistics, Imperial College London, London, UK; 39 Department of Cardiology, Ealing Hospital NHS Trust, Southall, UK; 40 Imperial College Healthcare NHS Trust, Imperial College London, London, UK; 41 Department of Medicine and Therapeutics, The Chinese University of Hong Kong, Hong Kong, Hong Kong; 42 Program for Neuropsychiatric Research, McLean Hospital, Belmont, MA USA; 43 Department of Psychiatry, Harvard Medical School, Boston, MA USA; 44 Department of Medicine, University of Mississippi Medical Center, Jackson, MI USA; 45 Department of Epidemiology, Colorado School of Public Health, Aurora, CP USA; 46 Department of Medicine and Pharmacology, University of Illinois at Chicago, Chicago, IL USA; 47 Department of Genetics, Texas Biomedical Research Institute, San Antonio, TX USA; 48 Department of Biostatistics, Boston University School of Public Health, Boston, MA USA; 49 Cardiac Arrhythmia Service and Cardiovascular Research Center, Massachusetts General Hospital, Boston, MA USA; 50 Cardiovascular Epidemiology and Genetics, Hospital del Mar Medical Research Institute (IMIM), Barcelona, Spain; 51 Centro de Investigación Biomédica en Red Enfermedades Cardiovaculares (CIBERCV), Barcelona, Spain; 52 Department of Medicine, Medical School, University of Vic-Central University of Catalonia, Vic, Spain; 53 Institute for Cardiogenetics, University of Lübeck, Lübeck, Germany; 54 DZHK (German Research Centre for Cardiovascular Research), partner site Hamburg/Lübeck/Kiel, Lübeck, Germany; 55 University Heart Center Lübeck, Lübeck, Germany; 56 Estonian Genome Center, Institute of Genomics, University of Tartu, Tartu, Estonia; 57 Clinic of Gastroenterology, Helsinki University and Helsinki University Hospital, Helsinki, Finland; 58 Institute of Clinical Molecular Biology (IKMB), Christian-Albrechts-University of Kiel, Kiel, Germany; 59 Cancer Genome Computational Analysis Group, Broad Institute of MIT and Harvard, Cambridge, MA USA; 60 Endocrinology and Metabolism Department, Hadassah-Hebrew University Medical Center, Jerusalem, Israel; 61 Department of Psychiatry and Behavioral Sciences, SUNY Upstate Medical University, Syracuse, NY USA; 62 Institute for Genomic Medicine, Columbia University Medical Center, Hammer Health Sciences, New York, NY USA; 63 Department of Genetics & Development, Columbia University Medical Center, Hammer Health Sciences, New York, NY USA; 64 Centro de Investigacion en Salud Poblacional, Instituto Nacional de Salud Publica, Cuernavaca, Mexico; 65 Genomics, Diabetes and Endocrinology, Lund University, Lund, Sweden; 66 Lund University Diabetes Centre, Malmö, Sweden; 67 Human Genetics Center, University of Texas Health Science Center at Houston, Houston, TX USA; 68 Department of Neurology, Columbia University, New York, NY USA; 69 Institute of Genomic Medicine, Columbia University, New York, NY USA; 70 Institute of Biomedicine, University of Eastern Finland, Kuopio, Finland; 71 Department of Psychiatry, Helsinki University Central Hospital, Lapinlahdentie, Helsinki, Finland; 72 Department of Medical Epidemiology and Biostatistics, Karolinska Institutet, Stockholm, Sweden; 73 Department of Neurology, Helsinki University Central Hospital, Helsinki, Finland; 74 Institute for Molecular Medicine FIMM, University of Helsinki, Helsinki, Finland; 75 Department of Public Health, University of Helsinki, Helsinki, Finland; 76 Department of Medicine, Harvard Medical School, Boston, MA USA; 77 Center for Genome Science, Korea National Institute of Health, Chungcheongbuk-do, South Korea; 78 MRC Centre for Neuropsychiatric Genetics & Genomics, Cardiff University School of Medicine, Cardiff, UK; 79 National Heart and Lung Institute and MRC London Institute of Medical Sciences, Imperial College London, London, UK; 80 Department of Health, THL-National Institute for Health and Welfare, Helsinki, Finland; 81 Section of Cardiovascular Medicine, Department of Internal Medicine, Yale School of Medicine, New Haven, CT USA; 82 Division of Pediatric Gastroenterology, Emory University School of Medicine, Atlanta, Georgia USA; 83 Department of Internal Medicine, Seoul National University Hospital, Seoul, South Korea; 84 The University of Eastern Finland, Institute of Clinical Medicine, Kuopio, Finland; 85 Kuopio University Hospital, Kuopio, Finland; 86 Department of Clinical Chemistry, Fimlab Laboratories and Finnish Cardiovascular Research Center-Tampere, Faculty of Medicine and Health Technology, Tampere University, Tampere, Finland; 87 The Mindich Child Health and Development Institute, Icahn School of Medicine at Mount Sinai, New York, NY USA; 88 Cardiac Arrhythmia Service, Massachusetts General Hospital, Boston, MA USA; 89 Department of Medicine and Therapeutics, The Chinese University of Hong Kong, Hong Kong, China; 90 Li Ka Shing Institute of Health Sciences, The Chinese University of Hong Kong, Hong Kong, China; 91 Hong Kong Institute of Diabetes and Obesity, The Chinese University of Hong Kong, Hong Kong, China; 92 Cardiovascular Research REGICOR Group, Hospital del Mar Medical Research Institute (IMIM), Barcelona, Spain; 93 Department of Genetics, Harvard Medical School, Boston, MA USA; 94 Oxford Centre for Diabetes, Endocrinology and Metabolism, University of Oxford, Churchill Hospital, Oxford, UK; 95 Wellcome Centre for Human Genetics, University of Oxford, Oxford, UK; 96 Oxford NIHR Biomedical Research Centre, Oxford University Hospitals NHS Foundation Trust, John Radcliffe Hospital, Oxford, UK; 97 F Widjaja Foundation Inflammatory Bowel and Immunobiology Research Institute, Cedars-Sinai Medical Center, Los Angeles, CA USA; 98 Atherogenomics Laboratory, University of Ottawa Heart Institute, Ottawa, Canada; 99 Division of General Internal Medicine, Massachusetts General Hospital, Boston, MA USA; 100 Department of Clinical Sciences, University Hospital Malmo Clinical Research Center, Lund University, Malmo, Sweden; 101 Lund University, Dept. Clinical Sciences, Skane University Hospital, Malmo, Sweden; 102 Instituto Nacional de Medicina Genómica (INMEGEN), Mexico City, Mexico; 103 Medical Research Institute, Ninewells Hospital and Medical School, University of Dundee, Dundee, UK; 104 Department of Molecular Medicine and Biopharmaceutical Sciences, Graduate School of Convergence Science and Technology, Seoul National University, Seoul, South Korea; 105 Department of Psychiatry, Keck School of Medicine at the University of Southern California, Los Angeles, CA USA; 106 Department of Psychiatry and Behavioral Sciences, Johns Hopkins University School of Medicine, Baltimore, MD USA; 107 Division of Genetics and Epidemiology, Institute of Cancer Research, London, UK; 108 Research Unit of Clinical Neuroscience, University of Oulu, Oulu, Finland; 109 Research Center, Montreal Heart Institute, Montreal, Quebec Canada; 110 Department of Medicine, Faculty of Medicine, Université de Montréal, Montreal, Quebec Canada; 111 Department of Public Health, Faculty of Medicine, University of Helsinki, Helsinki, Finland; 112 Department of Biomedical Informatics, Vanderbilt University Medical Center, Nashville, TN USA; 113 Department of Medicine, Vanderbilt University Medical Center, Nashville, TN USA; 114 Department of Biostatistics and Epidemiology, Perelman School of Medicine at the University of Pennsylvania, Philadelphia, PA USA; 115 Department of Medicine, Perelman School of Medicine at the University of Pennsylvania, Philadelphia, PA USA; 116 Center for Non-Communicable Diseases, Karachi, Pakistan; 117 National Institute for Health and Welfare, Helsinki, Finland; 118 Deutsches Herzzentrum München, Munich, Germany; 119 Technische Universität München, Munich, Germany; 120 Division of Cardiovascular Medicine, Nashville VA Medical Center and Vanderbilt University, School of Medicine, Nashville, TN USA; 121 Department of Psychiatry, Icahn School of Medicine at Mount Sinai, New York, NY USA; 122 Department of Genetics and Genomic Sciences, Icahn School of Medicine at Mount Sinai, New York, NY USA; 123 Institute for Genomics and Multiscale Biology, Icahn School of Medicine at Mount Sinai, New York, NY USA; 124 Institute of Clinical Medicine Neurology, University of Eastern Finland, Kuopio, Finland; 125 Department of Twin Research and Genetic Epidemiology, King’s College London, London, UK; 126 Departments of Genetics and Psychiatry, University of North Carolina, Chapel Hill, NC USA; 127 Saw Swee Hock School of Public Health, National University of Singapore, National University Health System, Singapore, Singapore; 128 Department of Medicine, Yong Loo Lin School of Medicine, National University of Singapore, Singapore, Singapore; 129 Duke-NUS Graduate Medical School, Singapore, Singapore; 130 Life Sciences Institute, National University of Singapore, Singapore, Singapore; 131 Department of Statistics and Applied Probability, National University of Singapore, Singapore, Singapore; 132 Folkhälsan Institute of Genetics, Folkhälsan Research Center, Helsinki, Finland; 133 HUCH Abdominal Center, Helsinki University Hospital, Helsinki, Finland; 134 Center for Behavioral Genomics, Department of Psychiatry, University of California, San Diego, CA USA; 135 Institute of Genomic Medicine, University of California, San Diego, CA USA; 136 Juliet Keidan Institute of Pediatric Gastroenterology, Shaare Zedek Medical Center, The Hebrew University of Jerusalem, Jerusalem, Israel; 137 Instituto de Investigaciones Biomédicas UNAM, Mexico City, Mexico; 138 Instituto Nacional de Ciencias Médicas y Nutrición Salvador Zubirán, Mexico City, Mexico; 139 Department of Public Health Solutions, National Institute for Health and Welfare, Helsinki, Finland; 140 Department of Psychiatry & Human Behavior, University of California Irvine, Irvine, CA USA; 141 Radcliffe Department of Medicine, University of Oxford, Oxford, UK; 142 Department of Gastroenterology and Hepatology, University of Groningen and University Medical Center Groningen, Groningen, The Netherlands; 143 Department of Physiology and Biophysics, University of Mississippi Medical Center, Jackson, MS USA; 144 Program in Infectious Disease and Microbiome, Broad Institute of MIT and Harvard, Cambridge, MA USA; 145 Center for Computational and Integrative Biology, Massachusetts General Hospital, Boston, MA USA

**Keywords:** Disease genetics, Medical genomics, Transcriptomics

Correction to: *Nature* 10.1038/s41586-020-2329-2Published online 27 May 2020

In this Article, author Marquis P. Vawter was missing from the Genome Aggregation Database Consortium list. They are associated with the affiliation: ‘Department of Psychiatry & Human Behavior, University of California Irvine, Irvine, CA, USA’, and contributed to the generation of the primary data incorporated into the gnomAD resource. The original Article has been corrected online.

