# Supplementary material for: Transcript expression-aware annotation improves rare variant interpretation
Source: Nature. 2020 May 27;581(7809):452–8. doi: 10.1038/s41586-020-2329-2 (PMC7334198; doi:10.1038/s41586-020-2329-2)
Supplement: Supplementary file 3 — This file contains Supplementary Figure 4: Baselevel TCF4 expression per GTEx tissue. [file 41586_2020_2329_MOESM3_ESM.pdf]

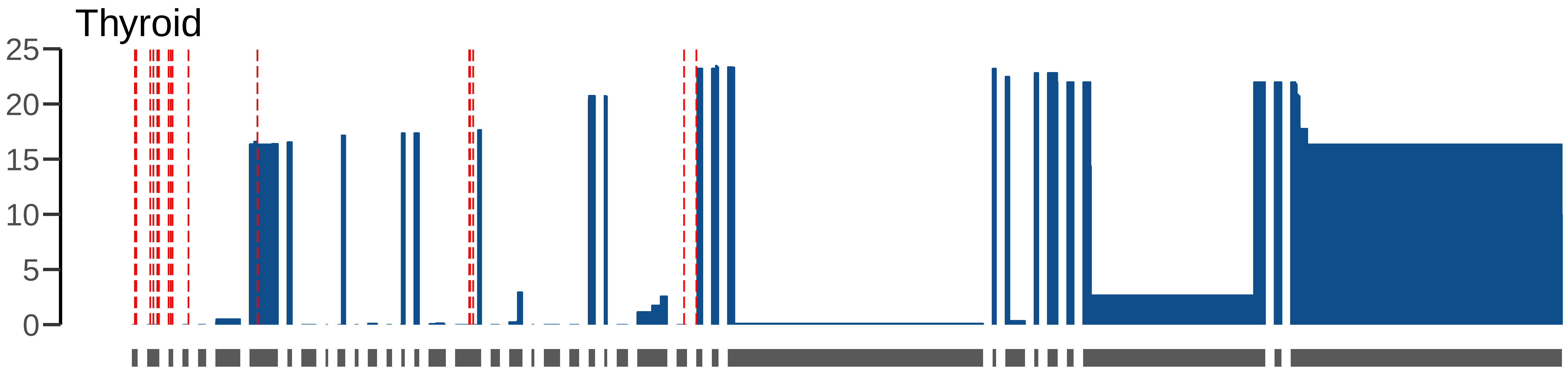

Thyroid
25
20
15
10
5
0

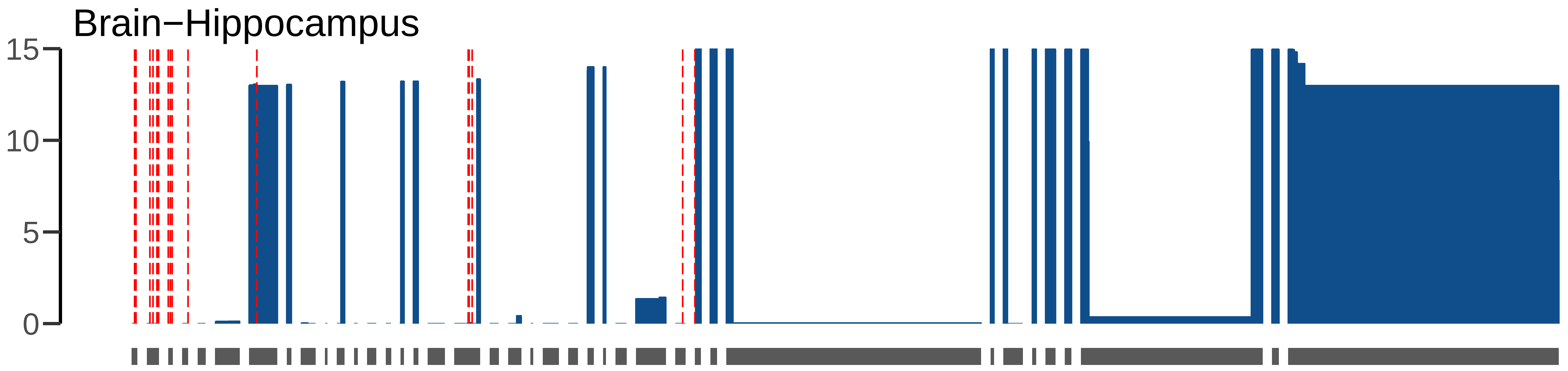
Brain−Hippocampus
15
10
5
0

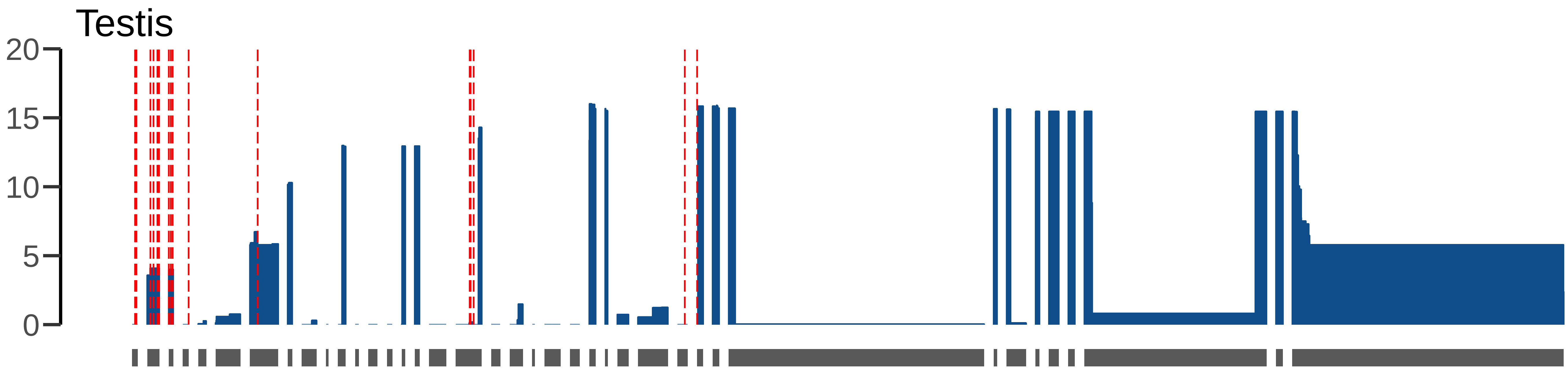
Testis
20
15
10
5
0

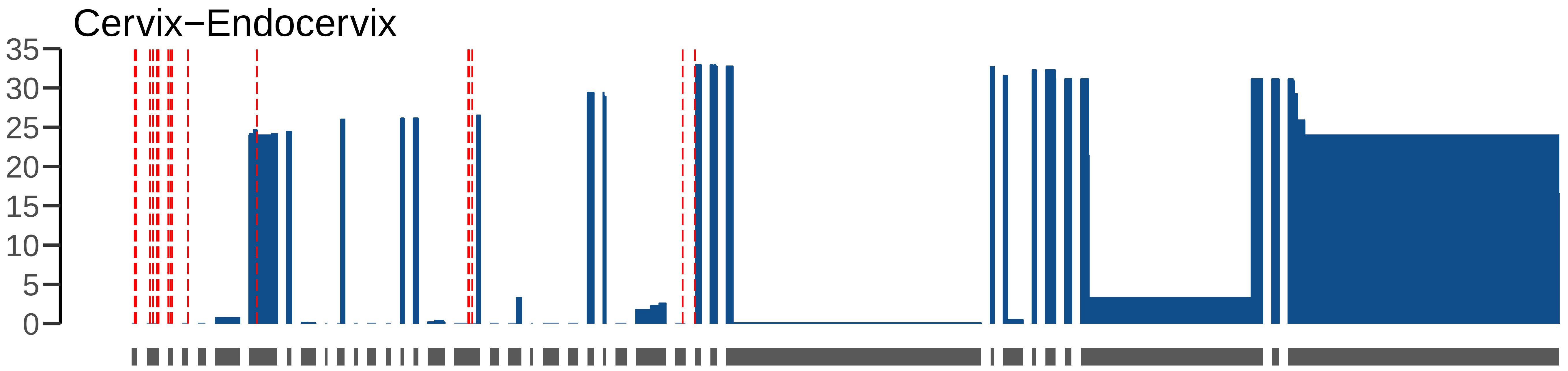
Cervix−Endocervix
35
30
25
20
15
10
5
0

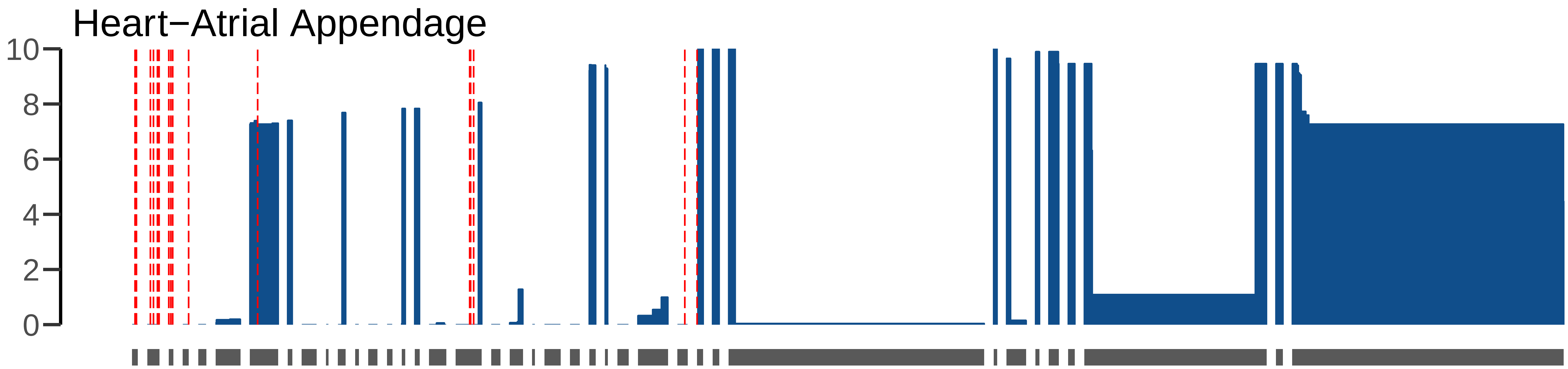
Heart−Atrial Appendage
10
8
6
4
2
0

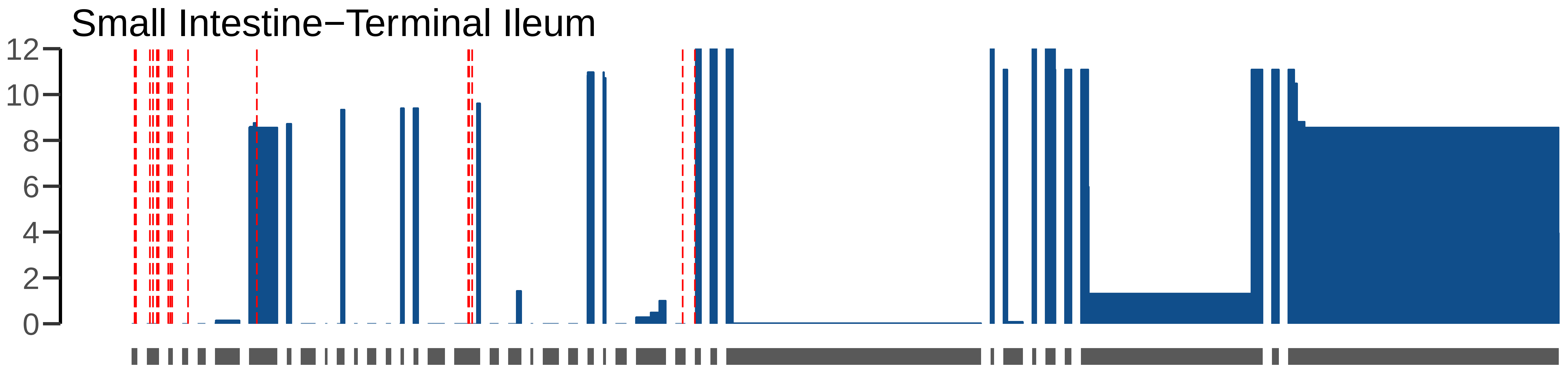
Small Intestine−Terminal Ileum
12
10
8
6
4
2
0

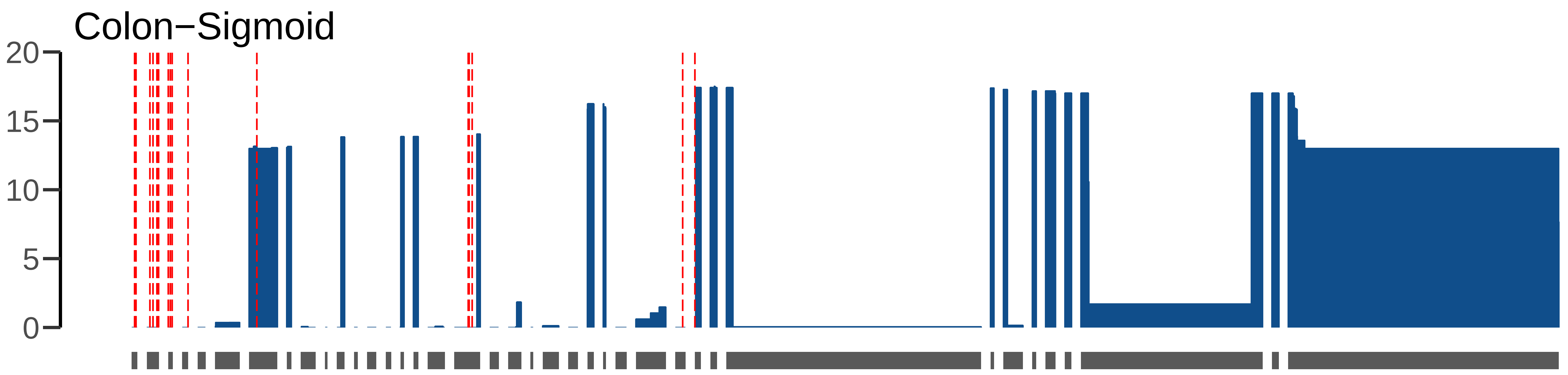

Colon−Sigmoid
20
15
10
5
0

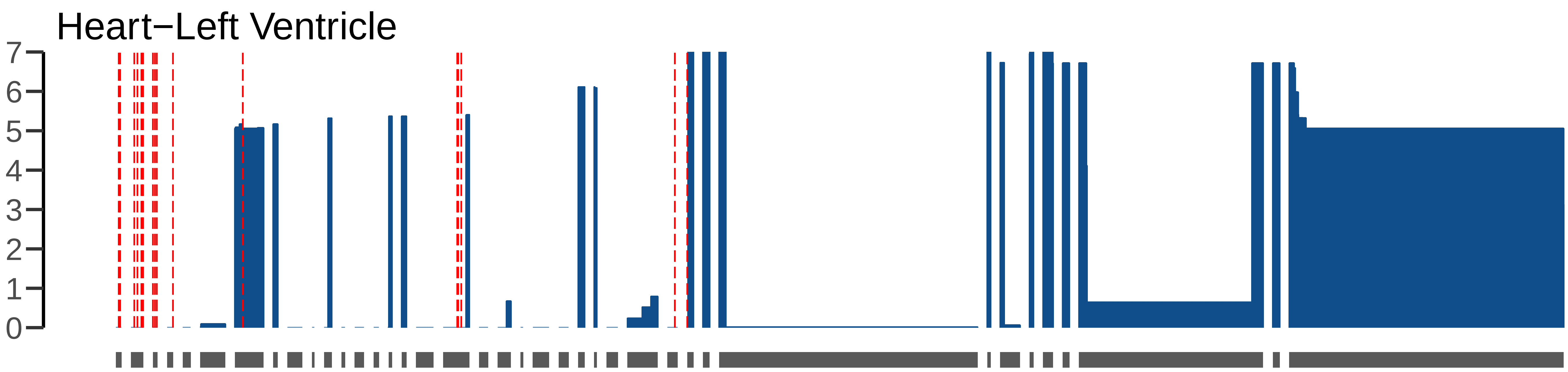
Heart−Left Ventricle
7
6
5
4
3
2
1
0

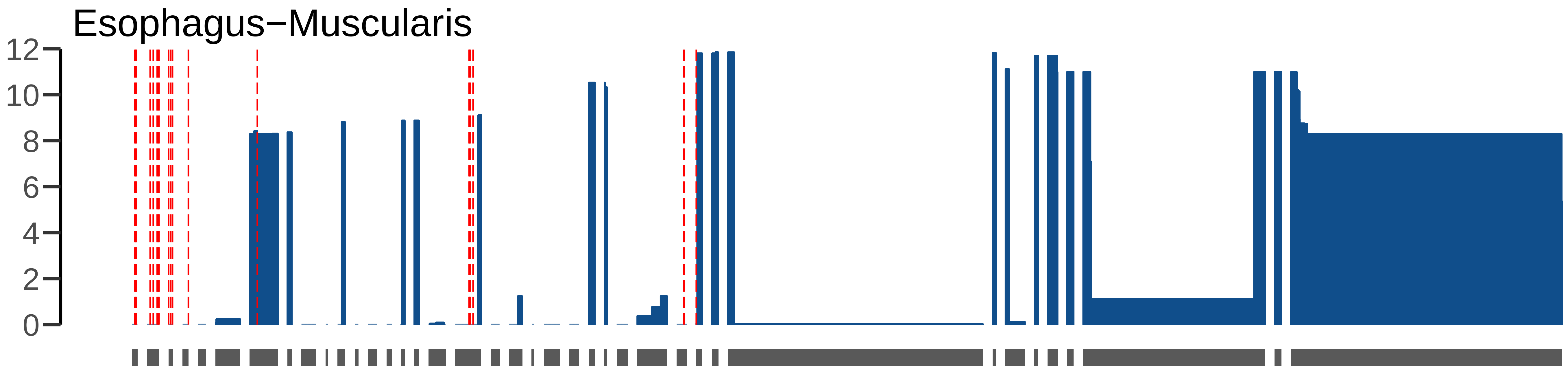

Esophagus−Muscularis
12
10
8
6
4
2
0

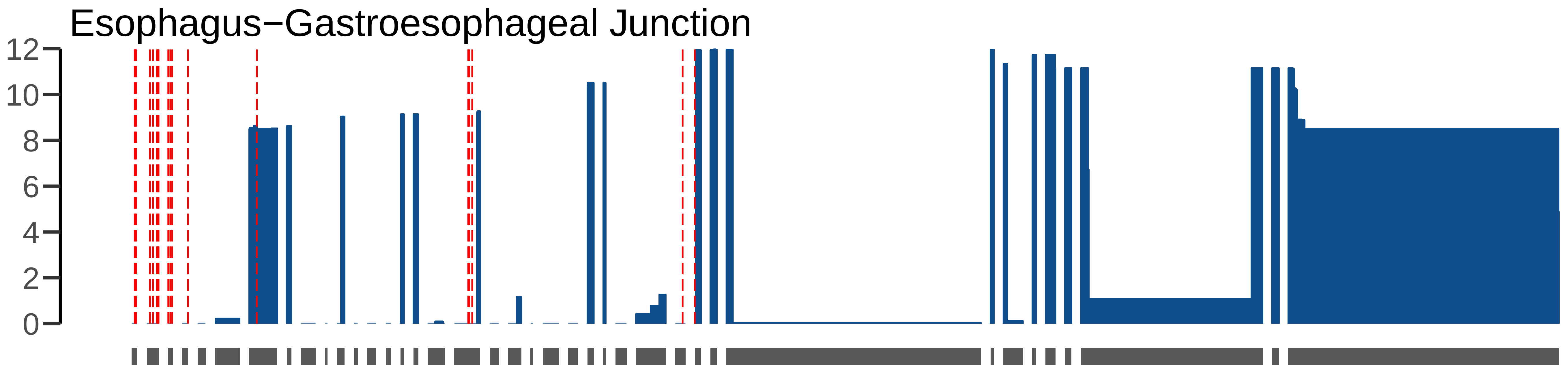
Esophagus−Gastroesophageal Junction
12
10
8
6
4
2
0

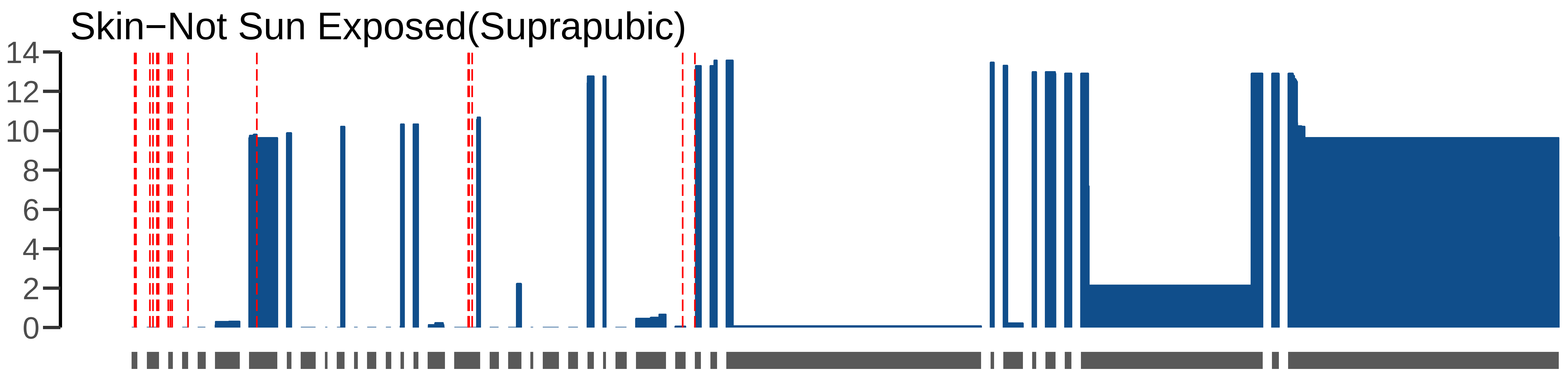
Skin−Not Sun Exposed(Suprapubic)
14
12
10
8
6
4
2
0

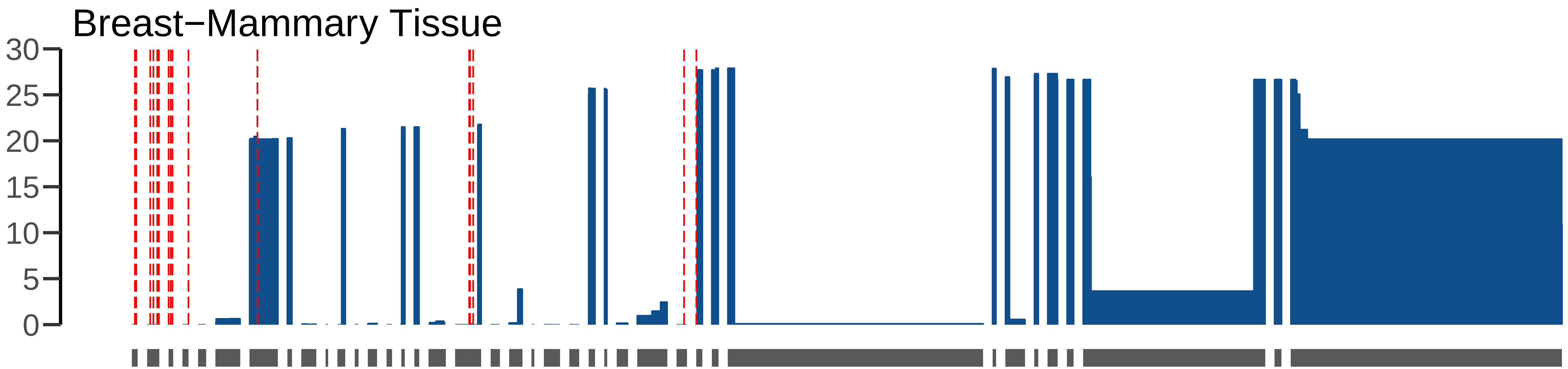
Breast−Mammary Tissue
30
25
20
15
10
5
0

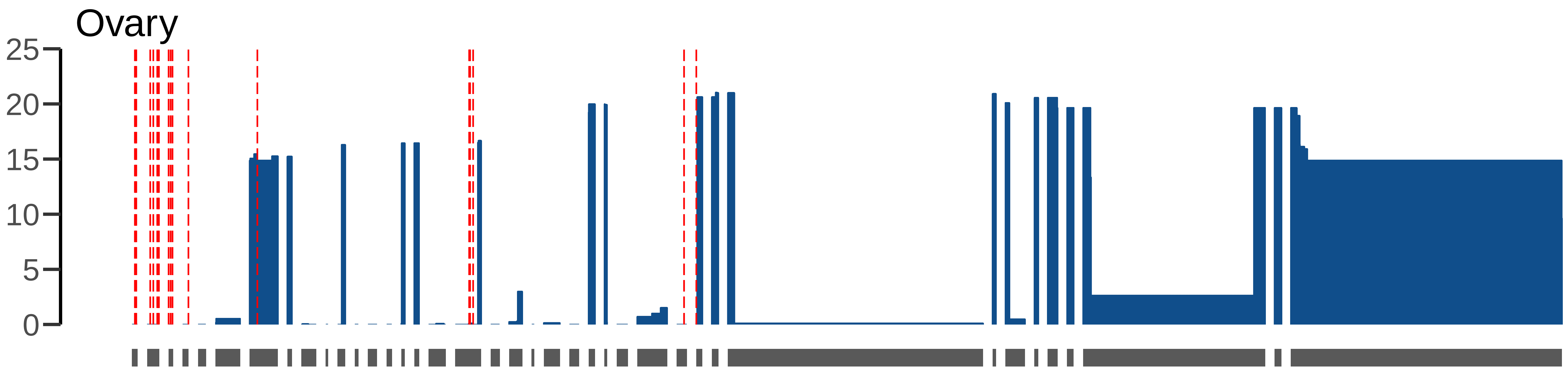

Ovary
25
20
15
10
5
0

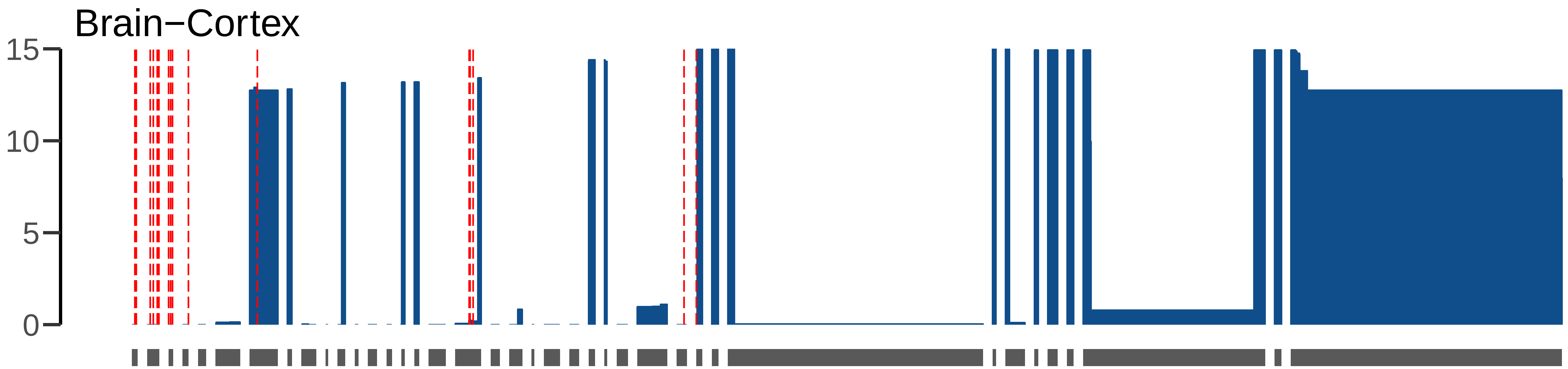

Brain−Cortex
15
10
5
0

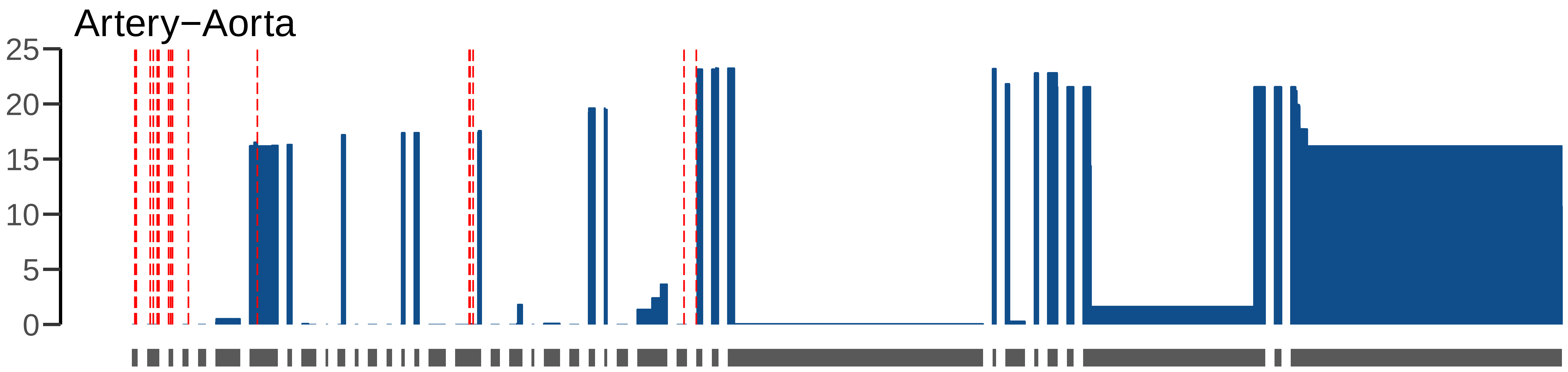
Artery−Aorta
25
20
15
10
5
0

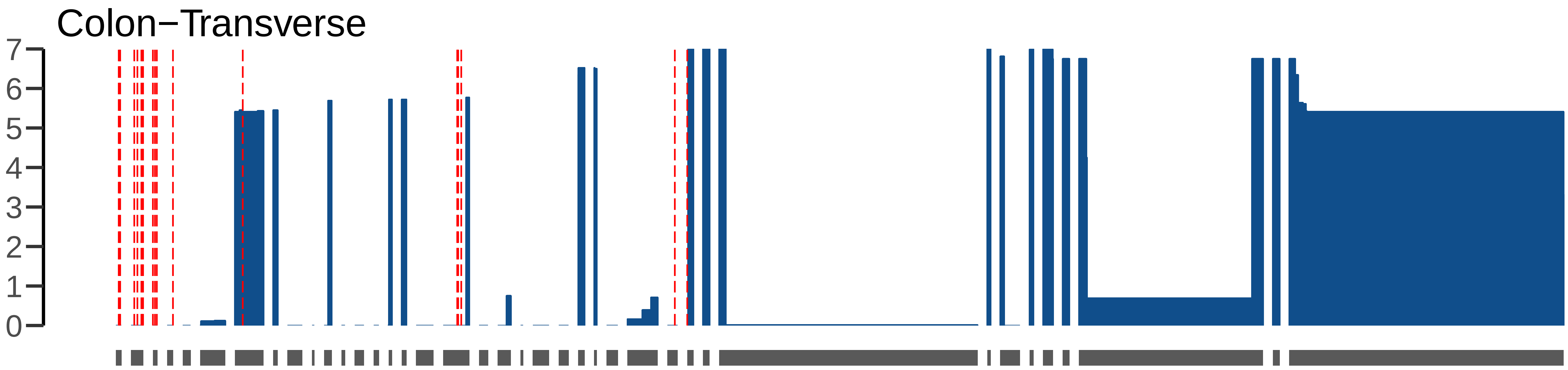

Colon−Transverse
7
6
5
4
3
2
1
0

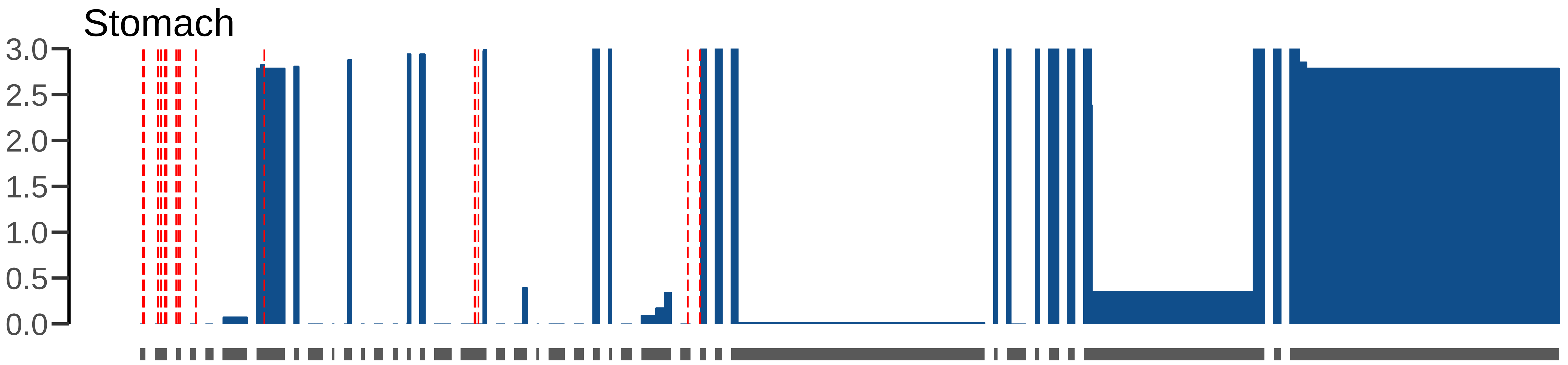
Stomach
3.0
2.5
2.0
1.5
1.0
0.5
0.0

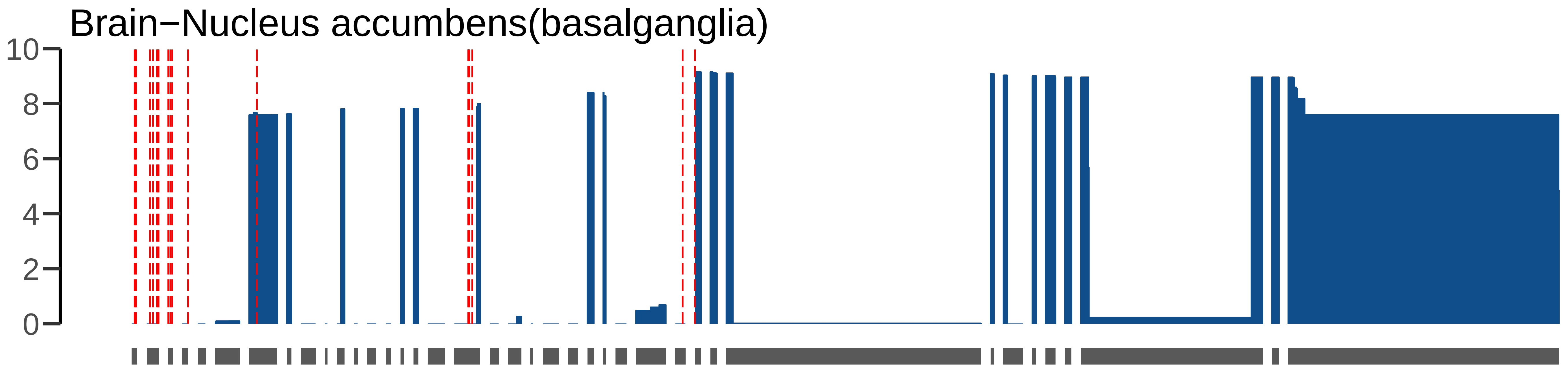

Brain−Nucleus accumbens(basalganglia)
10
8
6
4
2
0

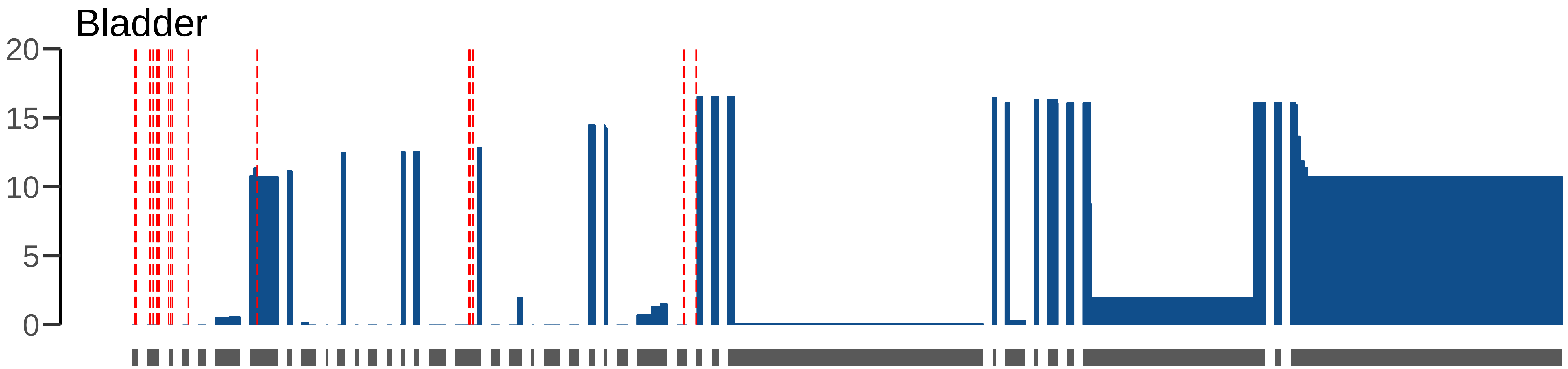
Bladder
20
15
10
5
0

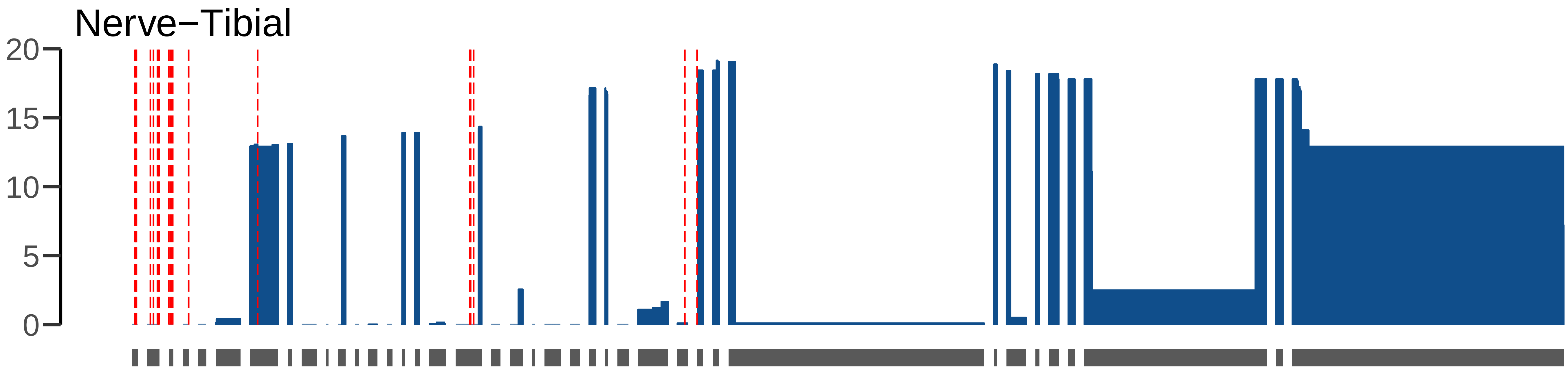
Nerve−Tibial
20
15
10
5
0

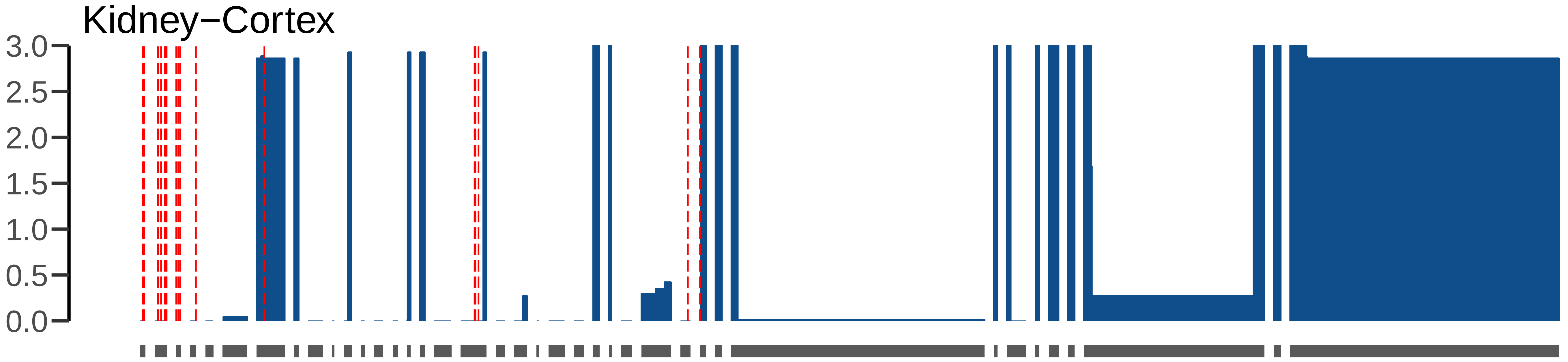

Kidney−Cortex
3.0
2.5
2.0
1.5
1.0
0.5
0.0

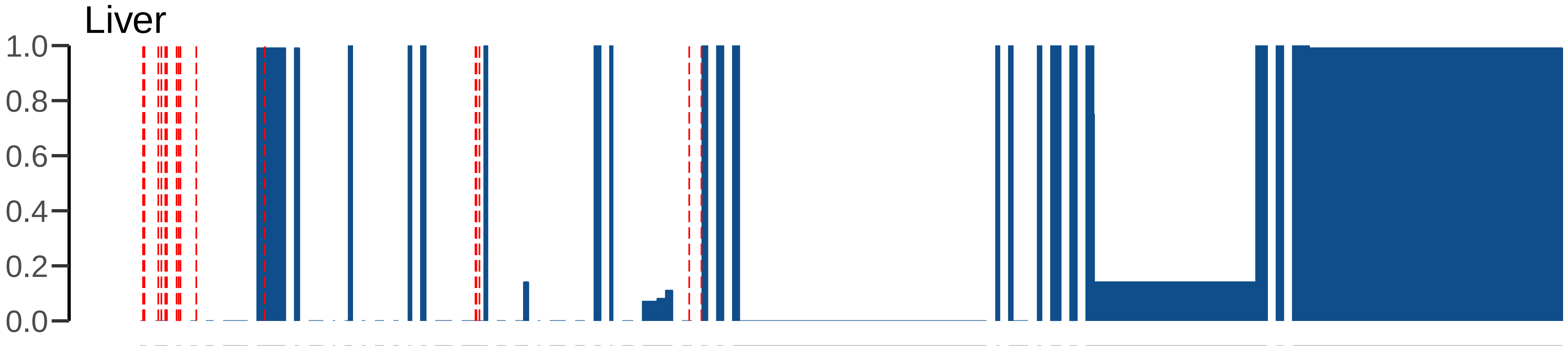
Liver
1.0
0.8
0.6
0.4
0.2
0.0

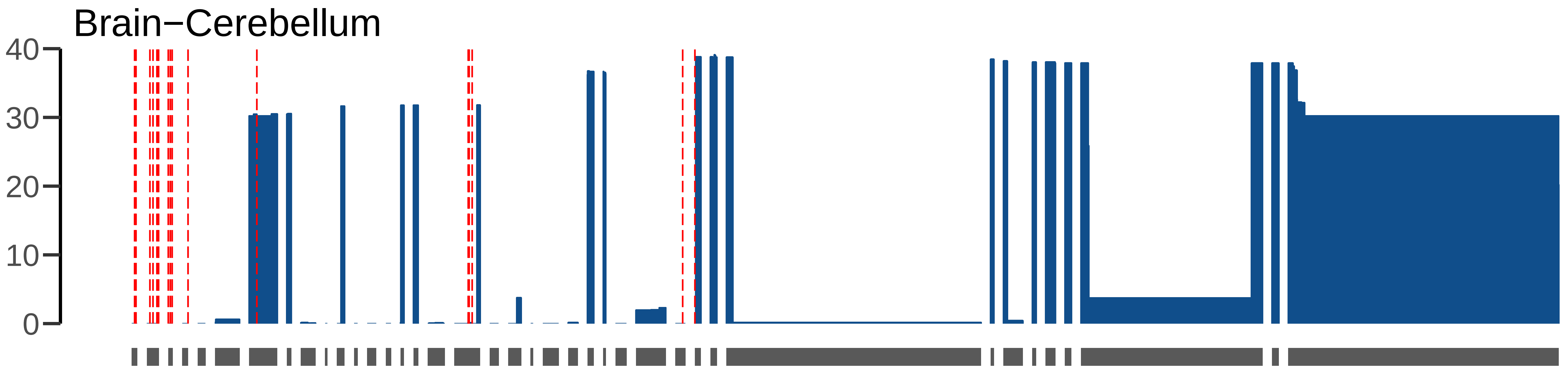
Brain−Cerebellum
40
30
20
10
0

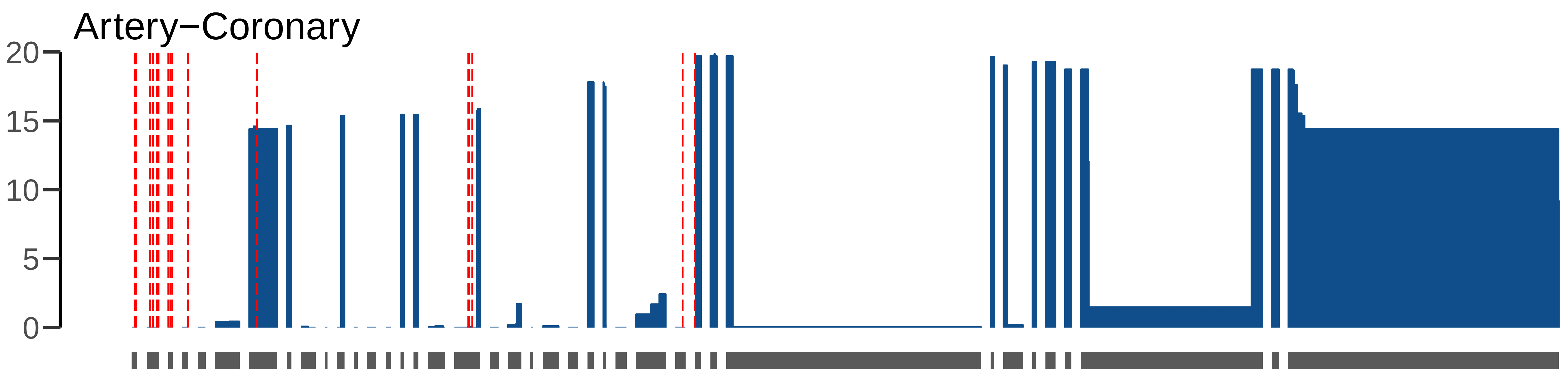
Artery−Coronary
20
15
10
5
0

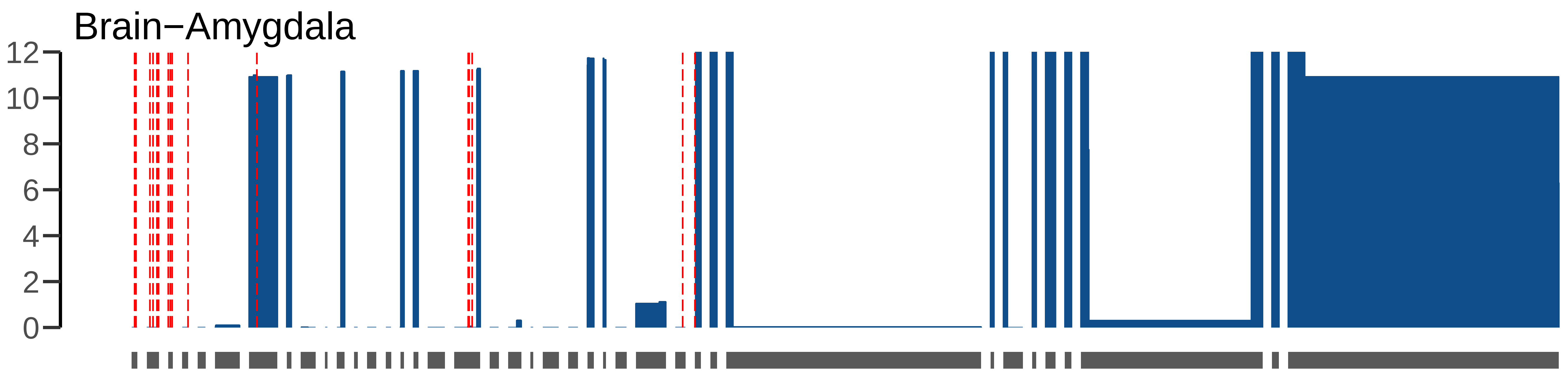
Brain−Amygdala
12
10
8
6
4
2
0

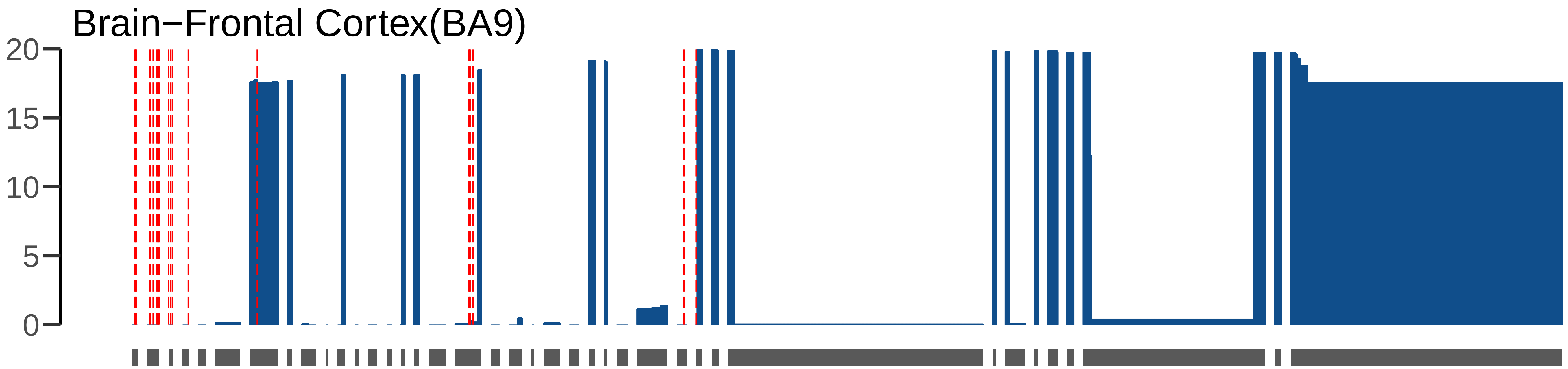

Brain−Frontal Cortex(BA9)
20
15
10
5
0

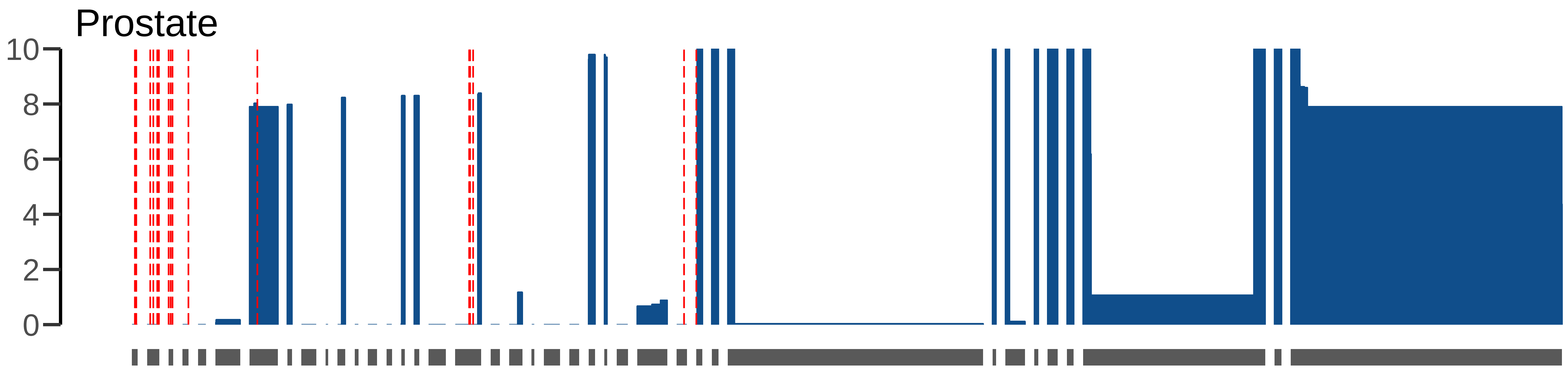
Prostate
10
8
6
4
2
0

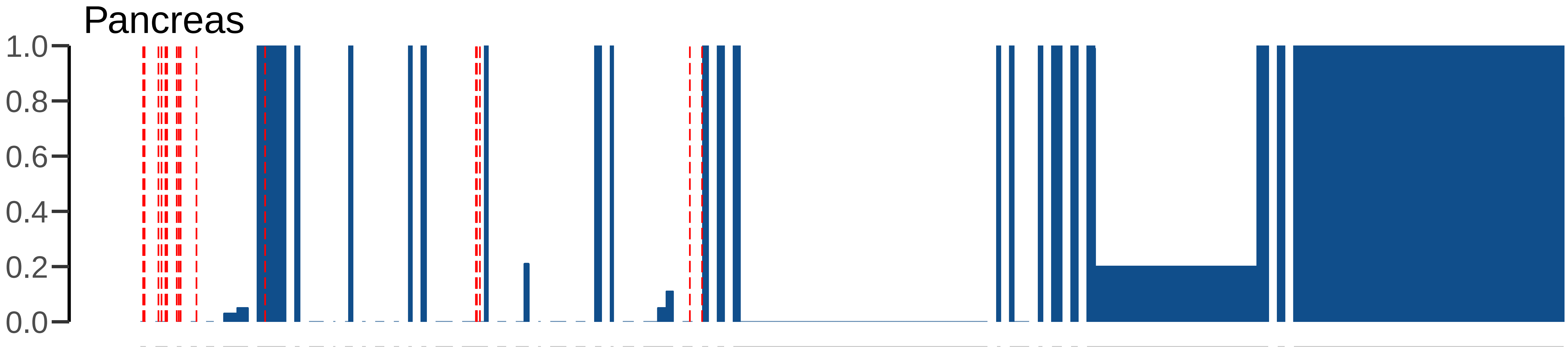
Pancreas
1.0
0.8
0.6
0.4
0.2
0.0

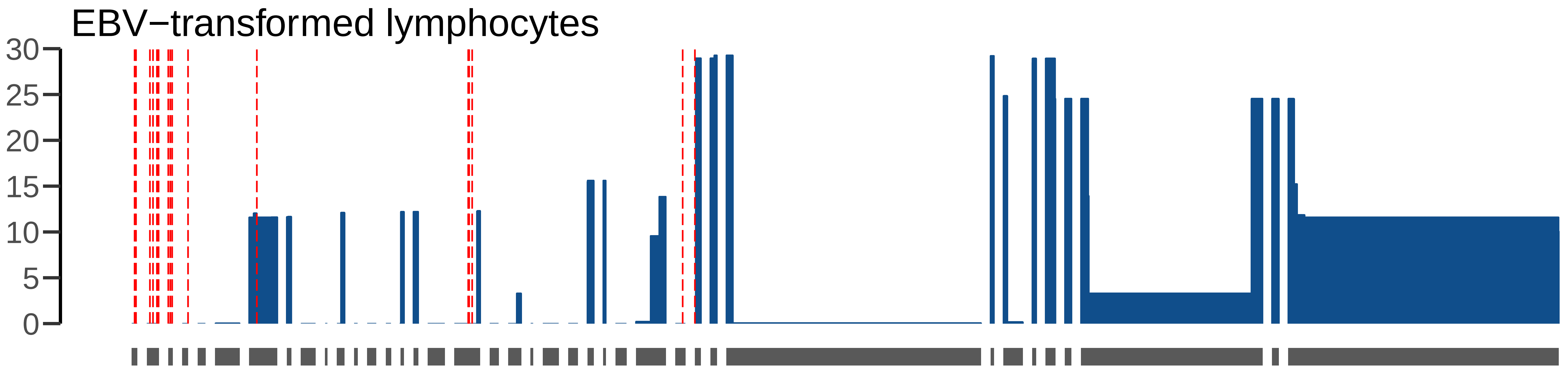
EBV−transformed lymphocytes
30
25
20
15
10
5
0

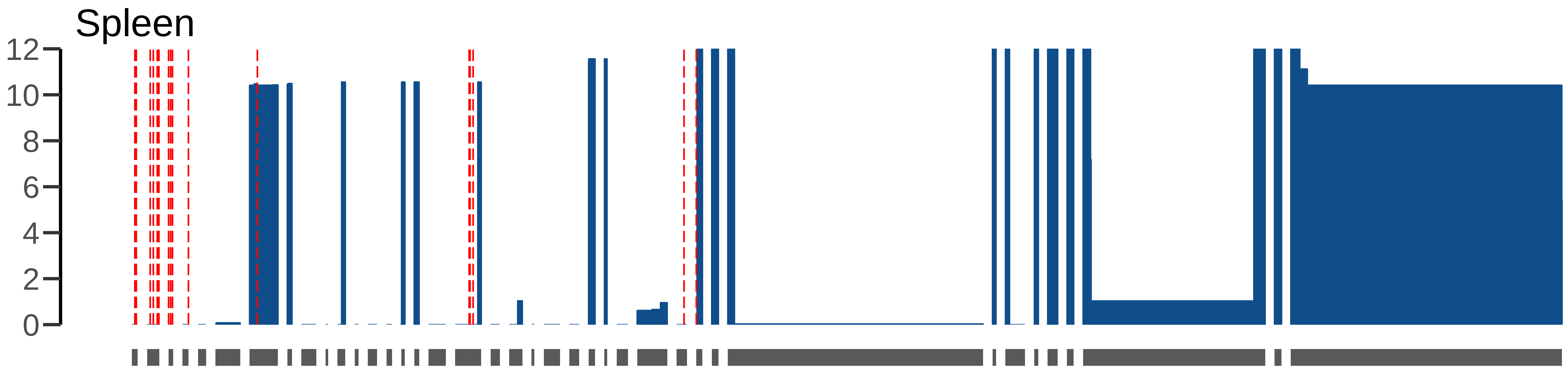
Spleen
12
10
8
6
4
2
0

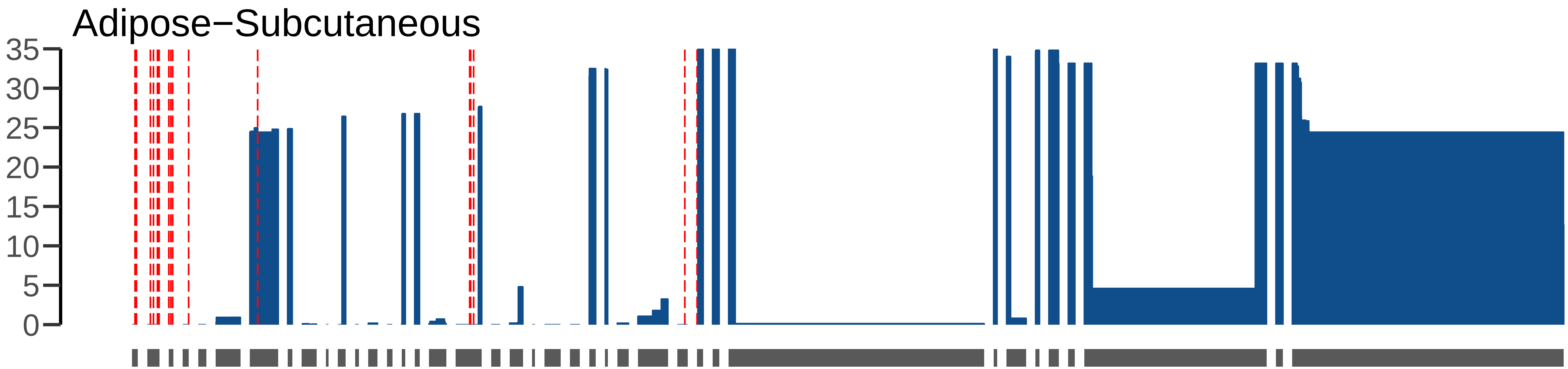

Adipose−Subcutaneous
35
30
25
20
15
10
5
0

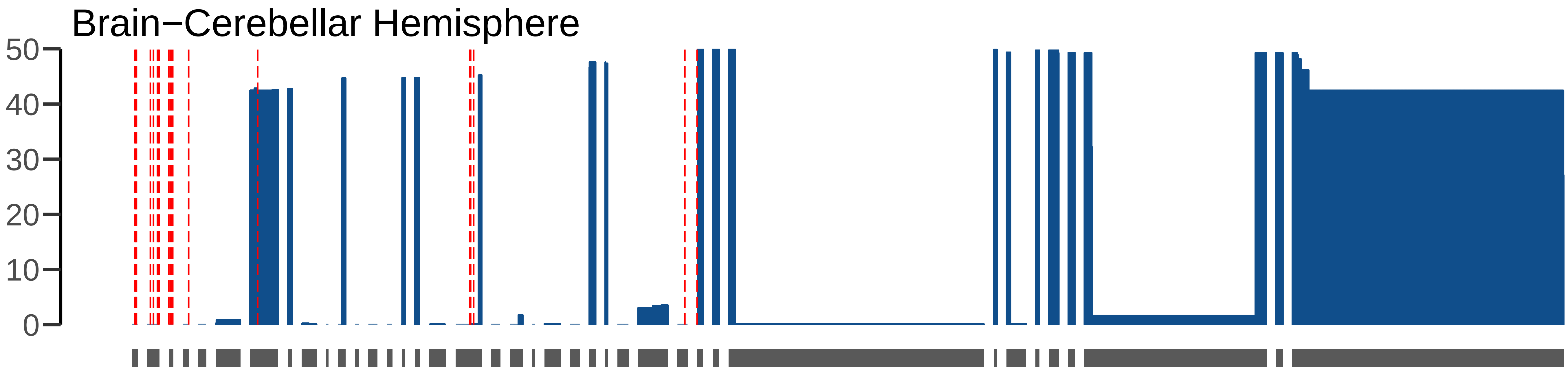

Brain−Cerebellar Hemisphere
50
40
30
20
10
0

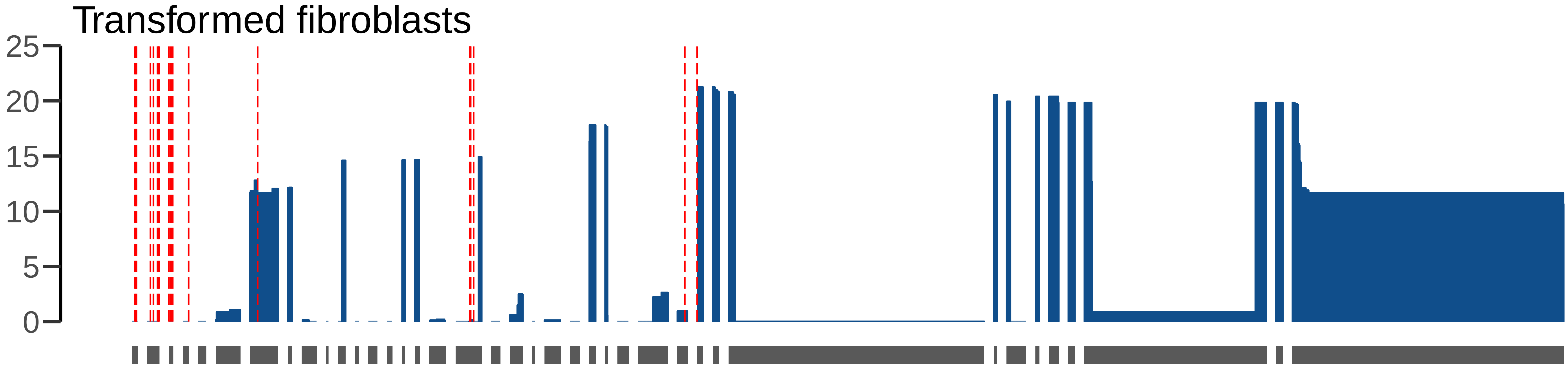

Transformed fibroblasts
25
20
15
10
5
0

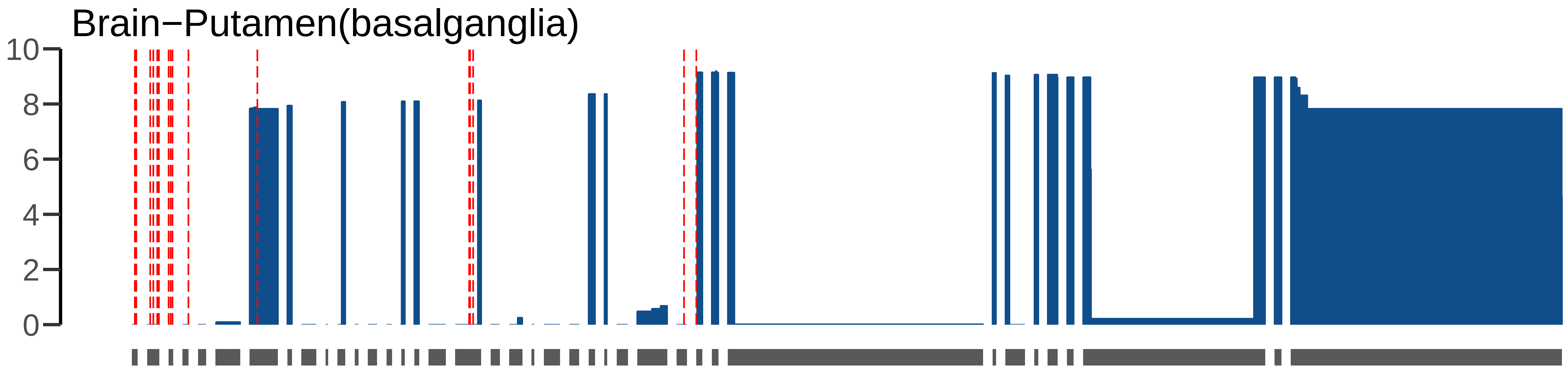
Brain−Putamen(basalganglia)
10
8
6
4
2
0

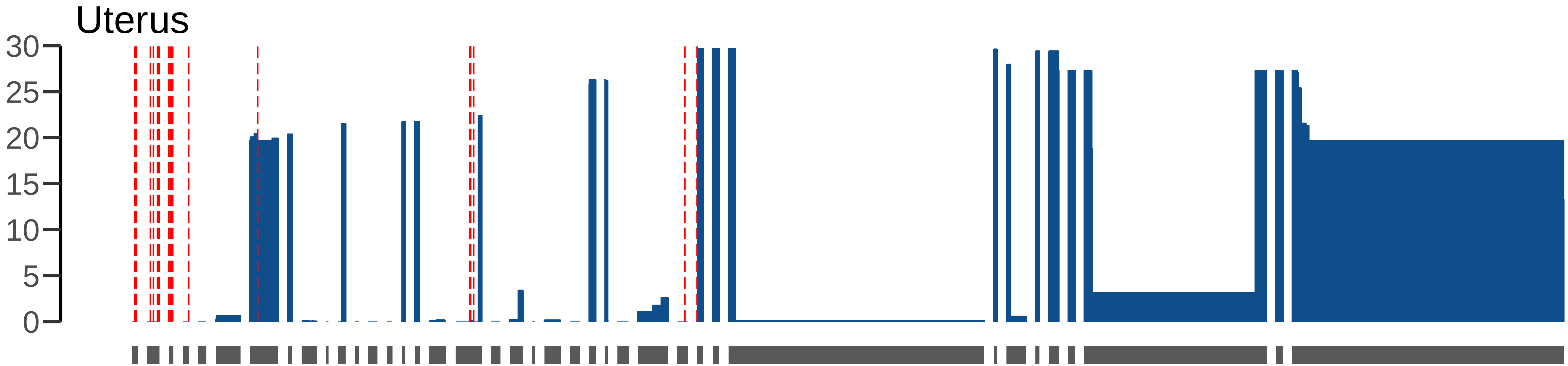
Uterus
30
25
20
15
10
5
0

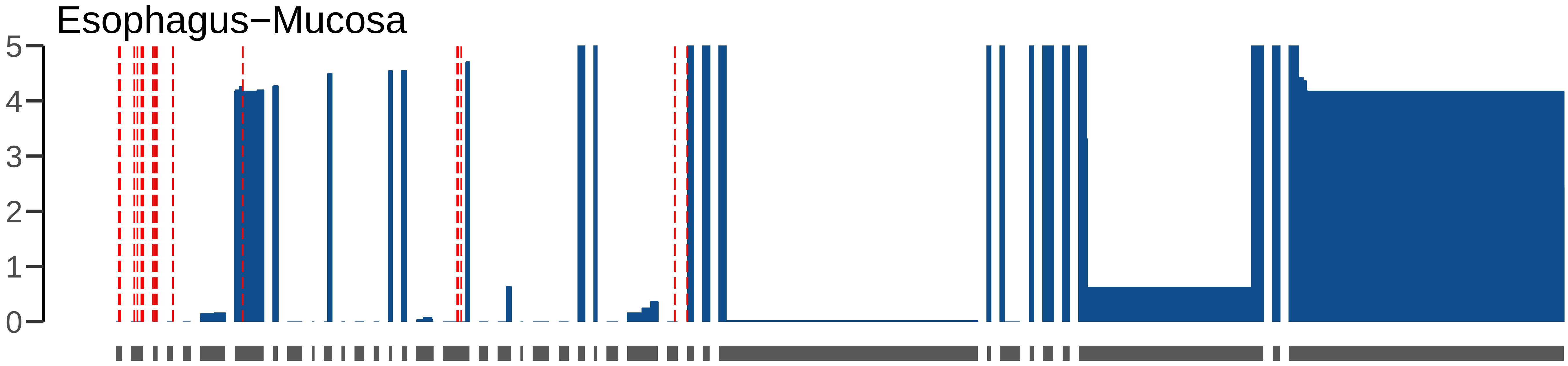
Esophagus−Mucosa
5
4
3
2
1
0

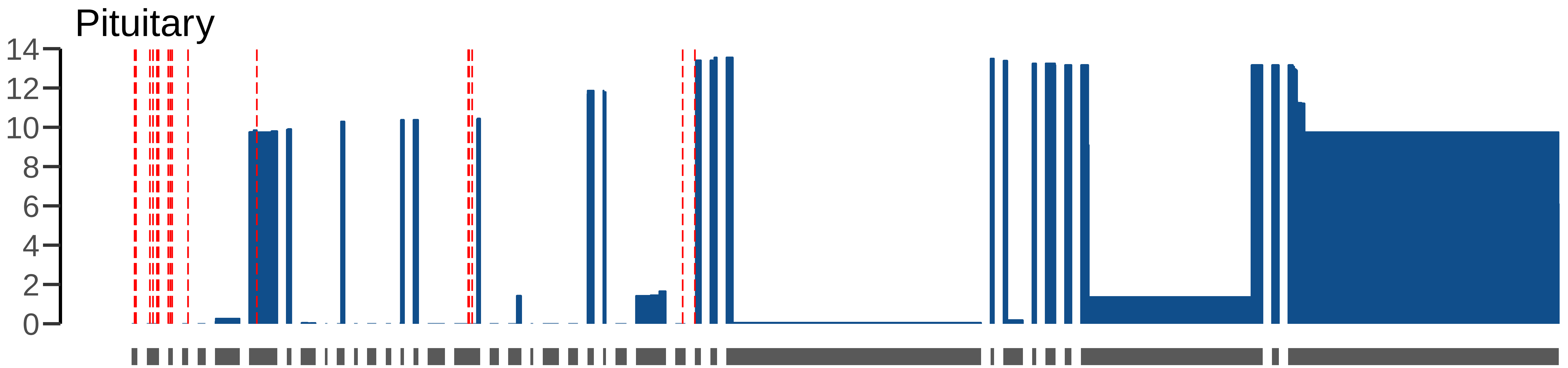
Pituitary
14
12
10
8
6
4
2
0

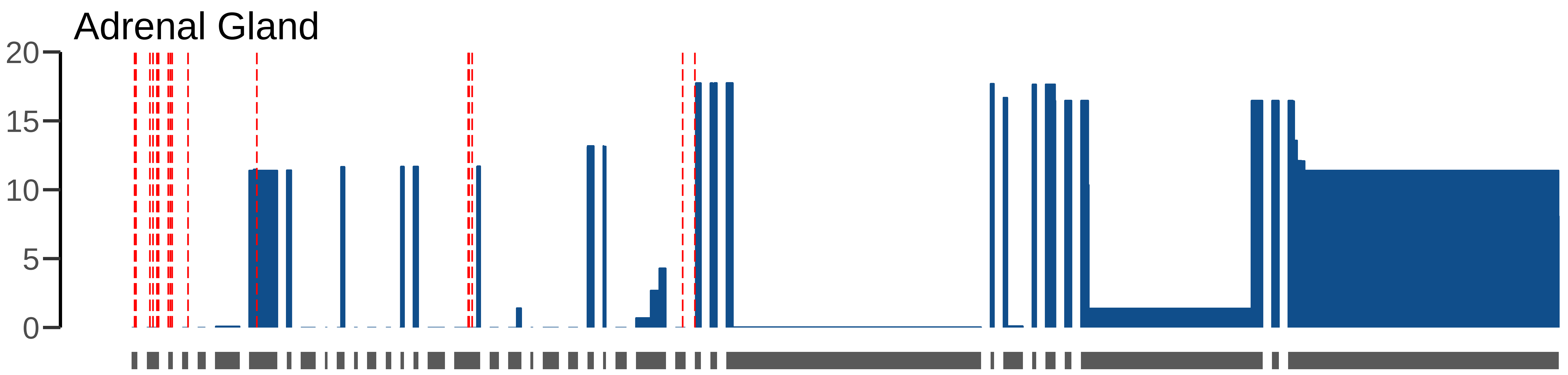
Adrenal Gland
20
15
10
5
0

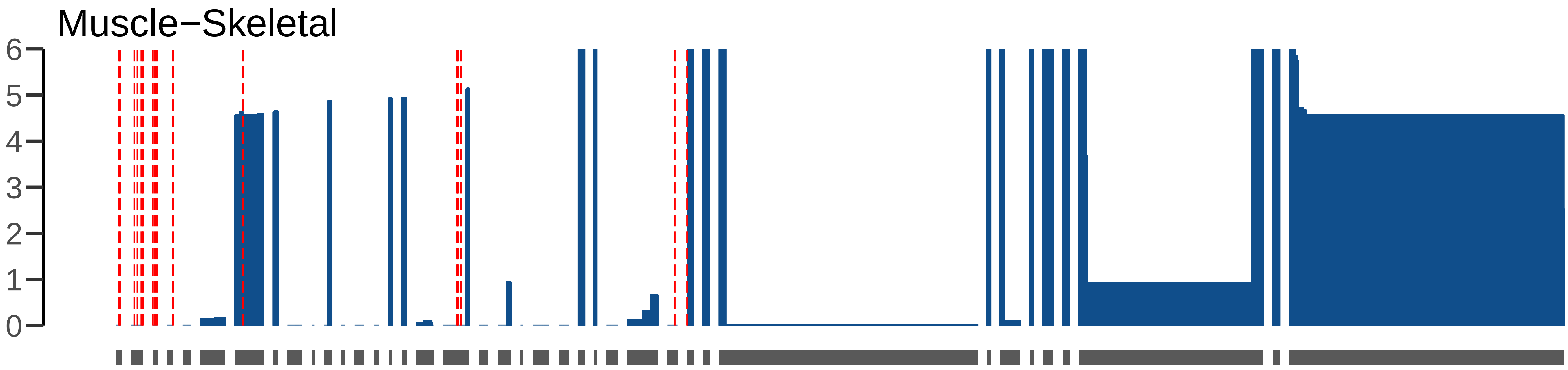
Muscle−Skeletal
6
5
4
3
2
1
0

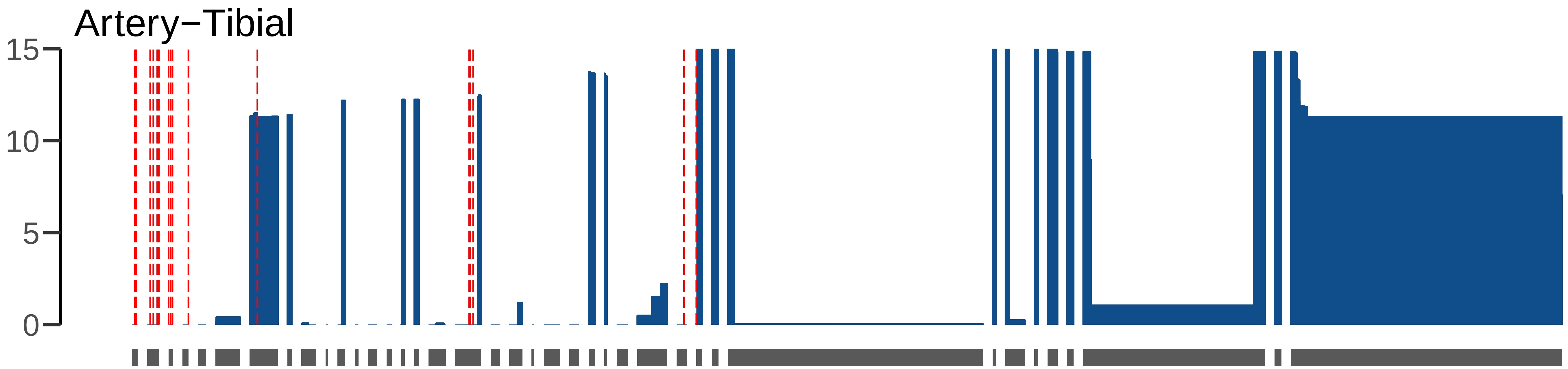

Artery−Tibial
15
10
5
0

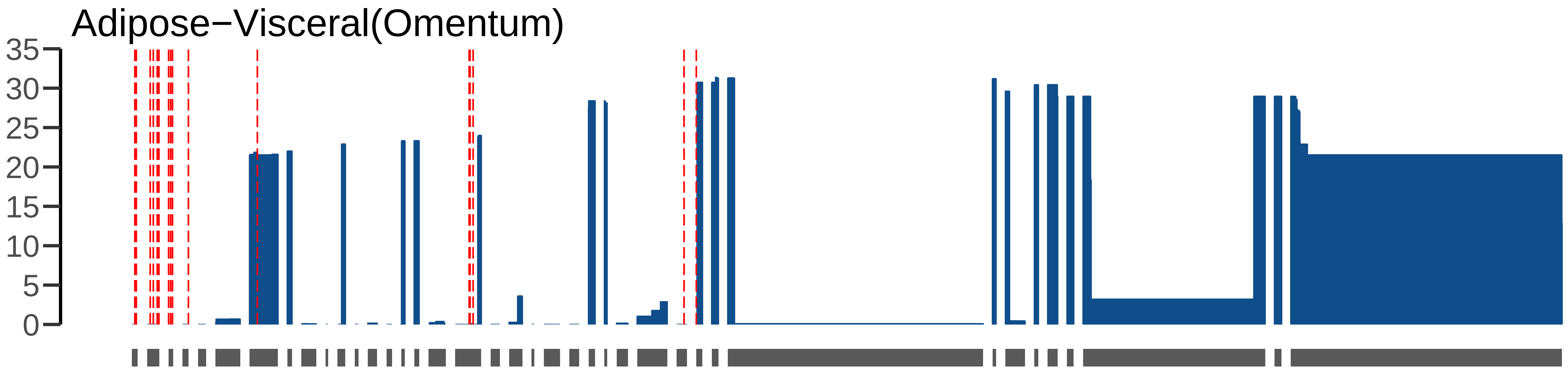
Adipose−Visceral(Omentum)
35
30
25
20
15
10
5
0

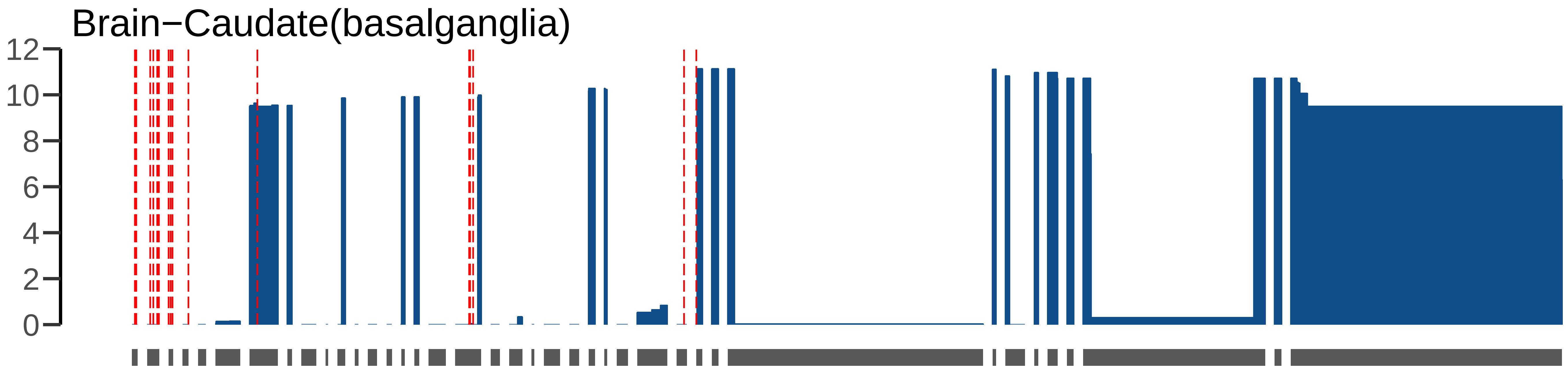

Brain−Caudate(basalganglia)
12
10
8
6
4
2
0

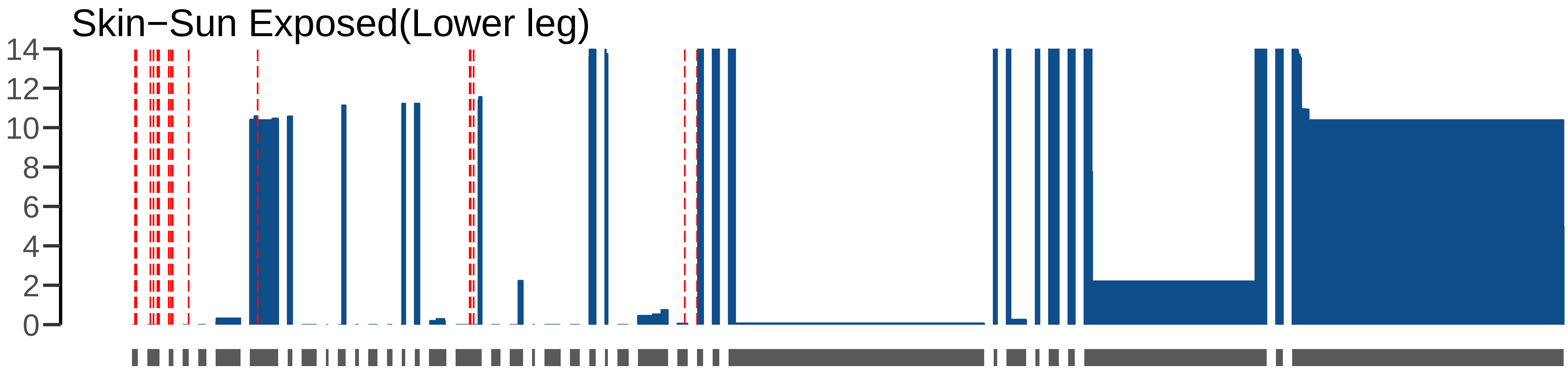
Skin−Sun Exposed(Lower leg)
14
12
10
8
6
4
2
0

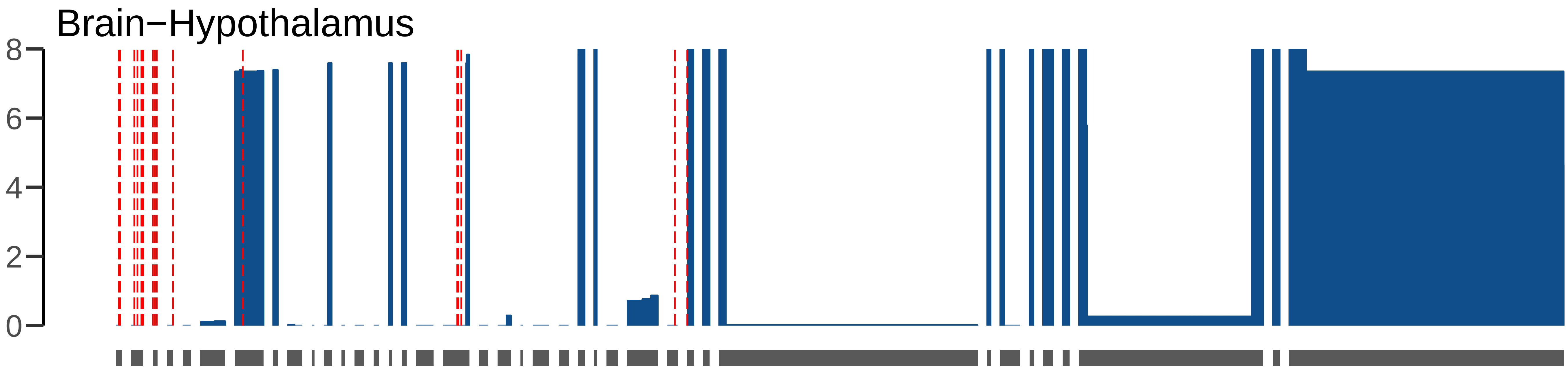

Brain−Hypothalamus
8
6
4
2
0

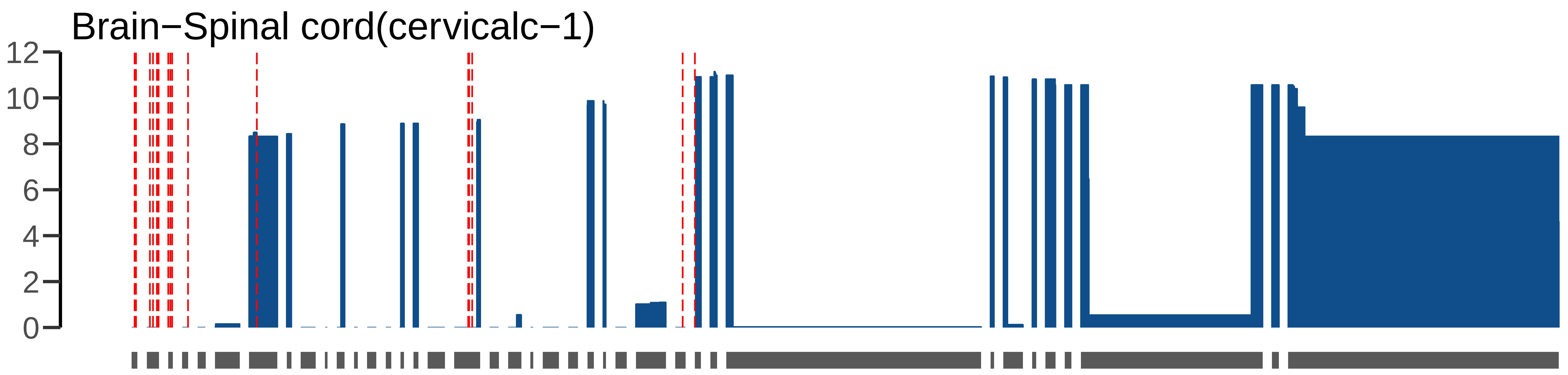
Brain−Spinal cord(cervicalc−1)
12
10
8
6
4
2
0

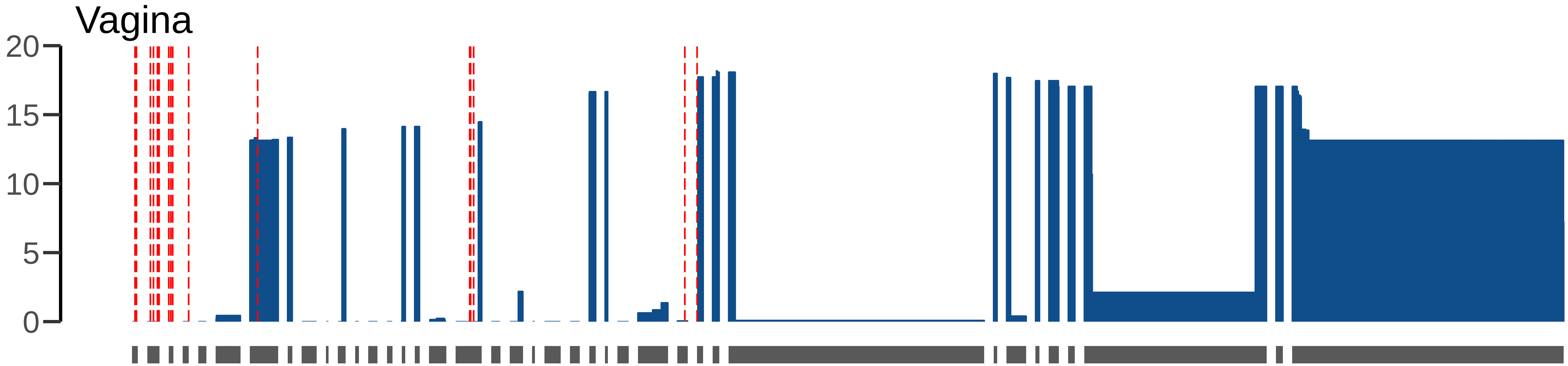
Vagina
20
15
10
5
0

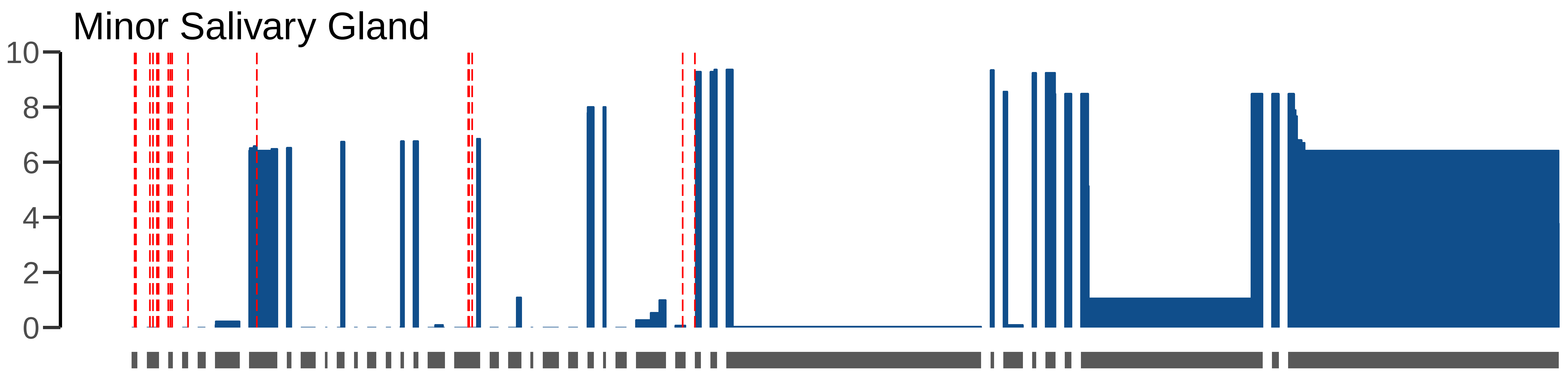
Minor Salivary Gland
10
8
6
4
2
0

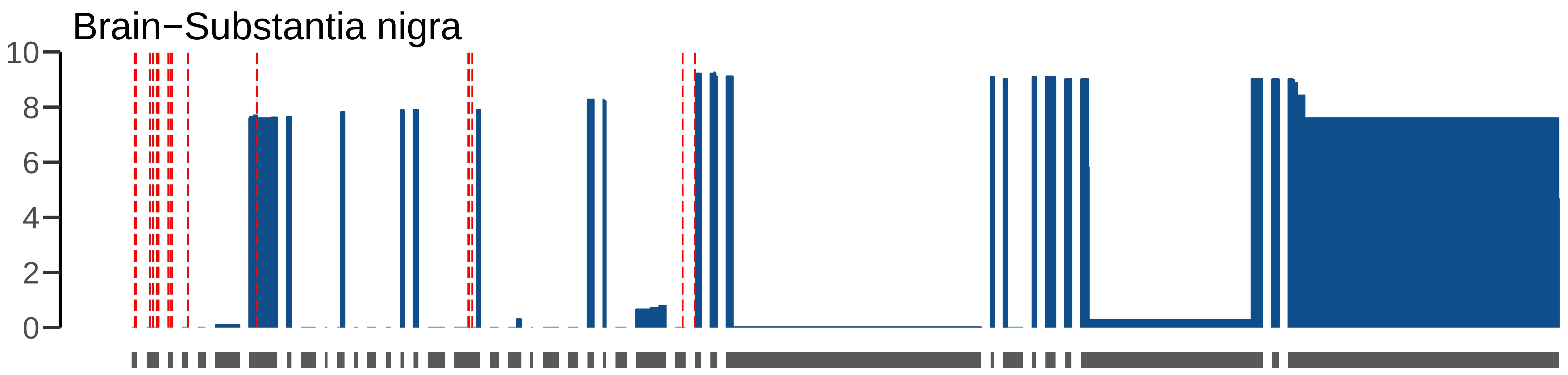

Brain−Substantia nigra
10
8
6
4
2
0

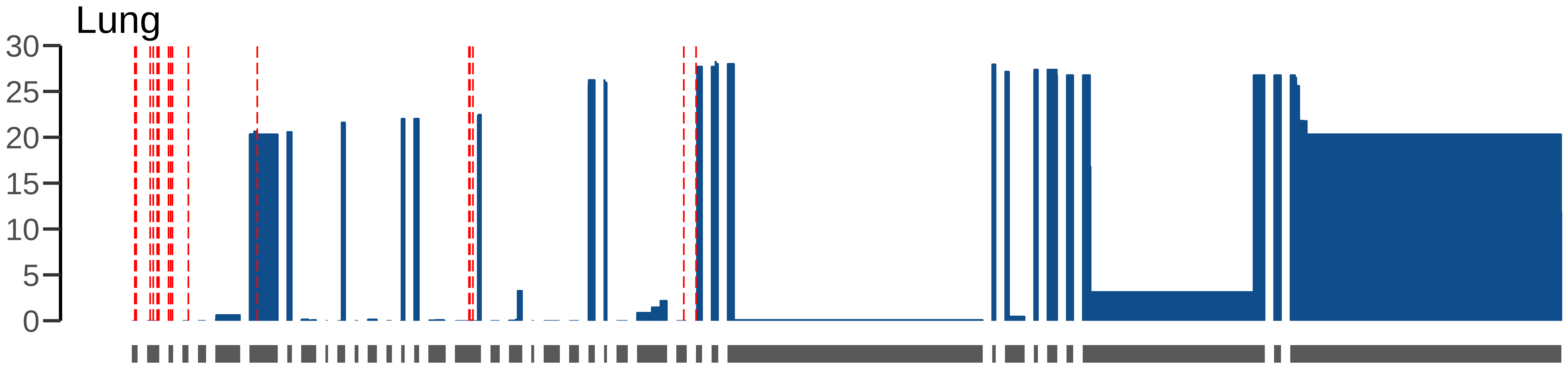
Lung
30
25
20
15
10
5
0

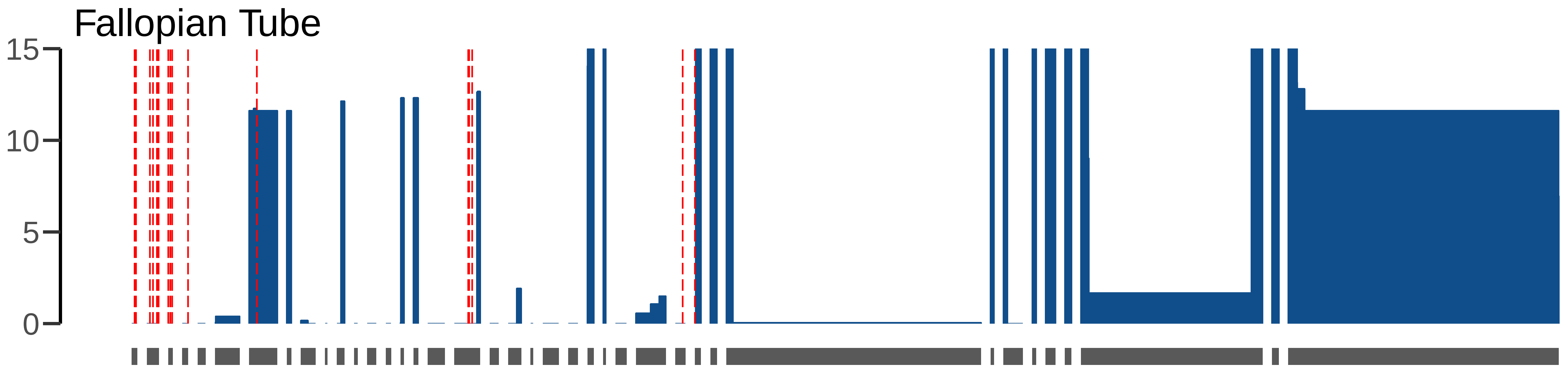

Fallopian Tube
15
10
5
0

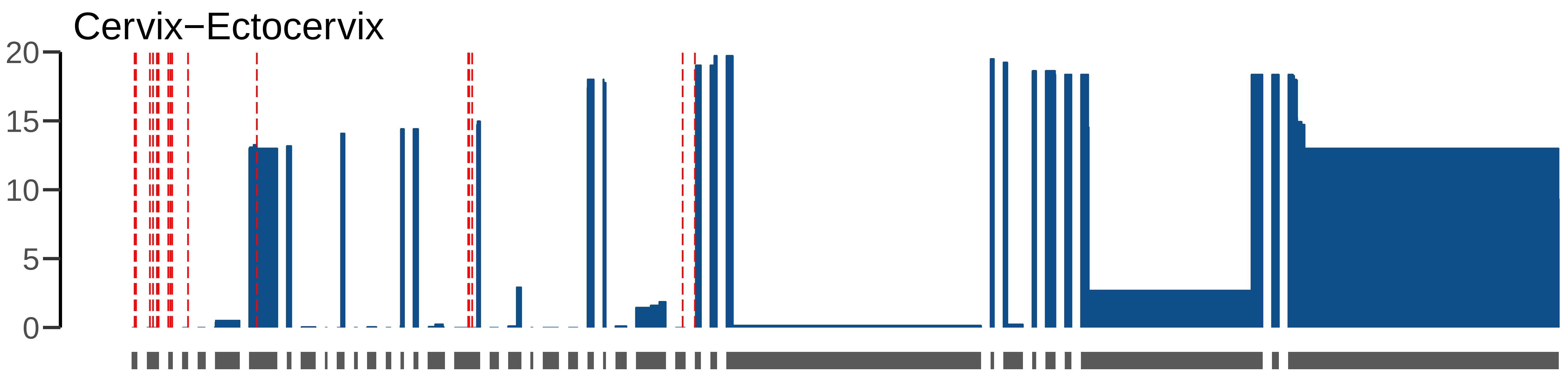
Cervix−Ectocervix
20
15
10
5
0

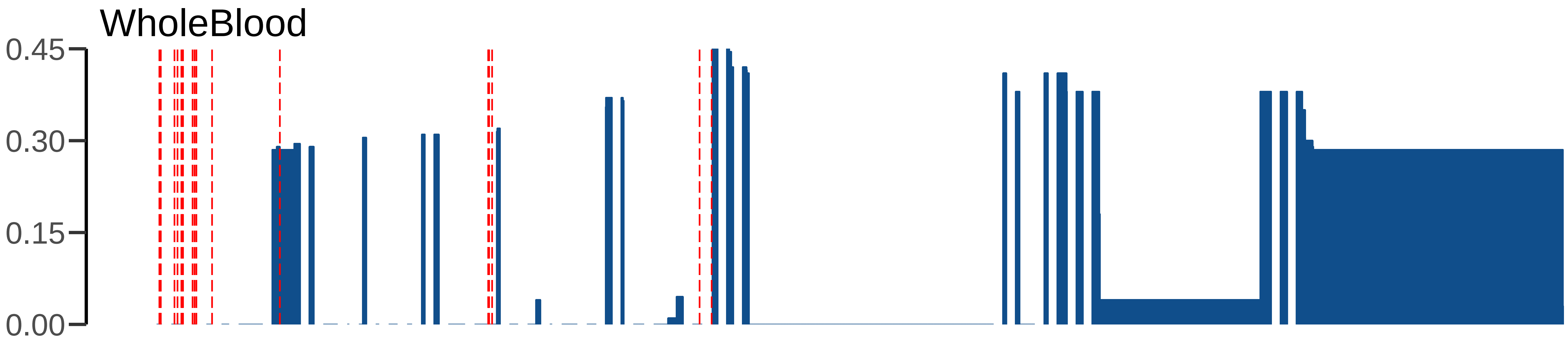

WholeBlood
0.45
0.30
0.15
0.00

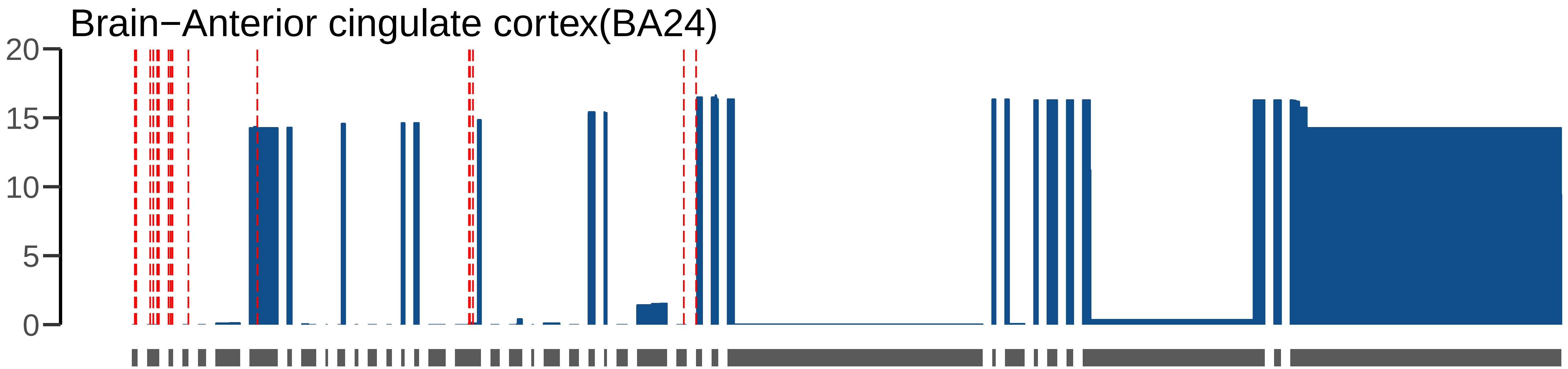
Brain−Anterior cingulate cortex(BA24)
20
15
10
5
0
