# Supplementary material for: Transcript expression-aware annotation improves rare variant interpretation
Source: Nature. 2020 May 27;581(7809):452–8. doi: 10.1038/s41586-020-2329-2 (PMC7334198; doi:10.1038/s41586-020-2329-2)
Supplement: Supplementary file 7 — Reviewer reports and authors' response from the peer review of this Article at Nature. [file 41586_2020_2329_MOESM7_ESM.pdf]

natureresearch

Peer Review File

Manuscript Title: Transcript expression aware annotation improves rare variant interpretation

Editorial Notes:

Reviewer Comments & Author Rebuttals

Reviewer Reports on the Initial Version:

Referee # 1 In this work, Cummings and colleagues presented a new metric, pext, to quantify the variation of variant effects in the same gene due to alternative splicing. They showed that regions with lower pext scores are less likely to be conserved across species, are depleted with known disease variants, and are depleted with de novo mutations identified in probands with severe neurodevelopmental disorders. This strategy is interesting and has the potential to aid variant interpretation in medical genetic studies. Also, this manuscript is very well written. However, I feel this work would be a better fit for a more specialized journal. Also, I am concerned about how much additional information for variant prioritization we could gain from pext after controlling for other well-known predictive features. I will elaborate on my concerns below.

Major comments:

1) Figure 2B clearly shows that LOFTEE prediction and mean pext score are not perfectly correlated. However, I don't think this figure is direct evidence to support the authors' argument that "pext provides orthogonal information to conservation for variant interpretation" (see line 145 in the main text). Indeed, another possibility is that the predictive power of pext is merely due to its correlation with sequence conservation. To rule out this explanation, the authors should check whether the high pext bin is still enriched with known ClinVar pathogenic variants after controlling for LOFTEE flag and other conservation scores, such as phyloP and phastCons scores. If pext still has significant predictive power after controlling for these confounding factors, then we can probably say that pext provides orthogonal information to sequence conservation for variant interpretation.

2) Similarly, the authors may investigate, after controlling for LOFTEE flag and other conservation scores as confounding factors, whether the high pext bin is still enriched with proband variants (Figure 5).

3) Many machine learning models, such as CADD and PolyPhen-2, have been developed to prioritize disease variants by integrating a large number of predictive features. If pext indeed provides orthogonal information for variant prioritization, the inclusion of pext as a new feature should significantly improve these machine learning models in the prediction of known disease variants. To test if this is the case, one could download the training data of a machine learning model, say CADD, add pext score as a new feature, and retrain the machine learning model. Then, the new model could be compared with the original model using standard ROC or precision-recall curves.

4) It is well known that alternatively spliced exons are more conserved than constitutively spliced exons (PMID: 12730695, 16157889, 16368777, and 16620375). These papers should be cited and discussed.

5) What's the recommended pext cutoff for variant prioritization? If we use a cutoff of 0.1, only a small fraction of putatively benign mutations will be filtered out (Figure 5). Can we do a better job by tweaking the cutoff? Is there any principled way to choose it?

Minor comments:

1) In Figure 3 and 5, are the error bars standard errors or confidence intervals?

2) Line 173: tends be -> tends to be

3) Line 217: fisher's -> Fisher's

4) Line 526: The commands to obtain the results is -> are

Referee # 3 The paper by Cummings et al. describe a method that uses transcriptomic data to help in assessing the functional impact of genetic variants. In short, the authors propose a measure, pext, which roughly corresponds, if I understood correctly, to the sum of the expression of all transcript isoforms including that variant over the total expression of the gene. The assumption is that variants with high pext score (i.e. in highly expressed transcripts) are likely to have larger impact than those with low score (i.e. in lowly expressed transcripts). pext would be ideally computed on large number of samples across multiple tissues, but in principle can computed using any number of samples. The assess the pext metric, the authors use the GTEx data set to compute it for a set of putative LoF variants found in 61 manually curated haploinsuficient developmental genes. The authors show that for low pext scores, a larger proportion of likely incorrect LoF variants are filtered out than of high quality pathogenic variants. Finally, they show that the pext score can be efficiently used to prioritize causative variants in a number of conditions.

It looks to me that the approach developed by the authors is sensible. The method, while straightforward, appears to be effective, and it has been overall well benchmarked. There is a lot of manual curation involved through the entire paper, which adds interest to the paper as resource. Also important as a resource is the precomputed values for every variant in the human genome. The paper is, thus, mostly methodological and does not bring many novel biological insights. I believe, for instance, that the relation between levels expression and sequence conservation has been quite well documented, and previous work should actually be referred.

The paper is overall clear and logically structured, and I do not have serious issues with it. There are a few things, however, for which I would like some. I also have a few more general comments and recommendations.

1. One of the issues that is less clear to me is that the 61 disease genes selected for evaluation are likely to be lethal during development (actually, this is the reason why they are selected). However, their expression is calculated in GTEx tissues that are all adults. That a transcript containing a pathogenic variant is not expressed in adult tissues, it does not mean that it is not expressed during development. Therefore, in my opinion, it would be much more informative to compute the pext score on developmental transcriptomic samples than in adult ones. Actually the authors allude to this in Figure 6D. Is there a reason why they do not employ transcriptomic developmental data (maybe combined with GTEx) trhough all the analysis?
2. The rationale for the authors to use transcript isoform quantification tools, rather than simply read coverage of variants, is that the tools would hopefully deal with the technical biases implicit in short read RNASeq (such as 3’ bias). This may be the case, but also, as the authors acknowledge, there are also many issues involved in the deconvolution of RNASeq reads to produce transcript quantifications. It is not at all clear that one compensates for the other. It maybe useful to compute the pext score based on read coverage and compare the results when computed based on transcript quantifications. If the results are comparable, there maybe a rationale to prefer read coverage, since it implies less data processing.
3. Related with the point above, the authors used an arbitrary metric, the average transcript expression across tissue samples, but, as they also acknowledge, other metrics could be used.

They explicitly mention the minimum expression (but it could also be the maximum). Since the computation of the pext score is quite efficient and inexpensive, it would really be useful to provide a comparative evaluation of the different such metrics. In general, the three comments above all refer to the investigation of the impact on the calculation of pext score of a number of factors: transcriptome dataset, method to compute the score and statistic to aggregate over samples. I think these analyses would provide a more general framework to understand the relevance of the method proposed.

4. There has been recently a quite intense debate on the relevance of alternative splicing in generating protein diversity, with claims that this relevance has been vastly overestimated (DOI:10.1016/j.tibs.2016.08.008) subsequently disputed (DOI:https://doi.org/10.1016/j.tibs.2017.04.001). It looks to me that the paper by Cummings et al. does support the relevance of alternative splicing in establishing phenotypes. Maybe the authors want to discuss on this.

Minor comments

1. Line 206. pLI is undefined. I believe it means PLoF intolerant.
2. Line 224. DD/ID should probably be introduced in line 221
3. Line 389. I believe is “a given variant”.
4. To me the only difference between Figure 3C and 5D is the inclusion of the missense variants in the latter, but other than that they are identical. I am not sure that the two figures are needed.

Referee # 4 Cummings et al. develop a novel annotation metric (i.e., pext) that summarizes exon expression information across transcripts and describe a new annotation tool that uses this information to facilitate variant interpretation. They demonstrate that development of an expression-based annotation metric addresses a common confounder (i.e., transcript errors) of variant annotation and that the tools is fairly effective at flagging falsely annotated pLOF variants. More importantly, they show that expression-based annotation identifies functionally important regions of the genome, provides orthogonal information to conservation estimates, and that it can be used to facilitate interpretation of variant with large, deleterious effects that underlie Mendelian conditions. The manuscript is clear and well-written, the conclusions well-supported, and the applicability nicely illustrated. I have no major concerns about the strategy used to develop the annotation metric, the use cases presented, or it’s potential usefulness to the field. All of this withstanding, it’s not clear to me that this metric or the tool developed represents such a major advance as to warrant publication in Nature. Indeed, the manuscript seems like other reports of new annotators, tools, etc. that are commonly published in discipline-specific human genetics journals. It's solid but not exceptional.

Author Rebuttals to Initial Comments:

We thank the reviewers for their comments. We have gone through each comment and updated the manuscript to reflect improvements in the main text and supplementary information sections as suggested.

We note that the reviewers raised few technical concerns about the analyses and conclusions presented in the manuscript. However, the reviewers suggested the scientific conclusions in the manuscript may not be of broad interest. We would like to address this thematic concern by emphasizing the broad implications of our work in human genetics, both as a framework for variant interpretation and as a resource for the wider community.

Since their inception, the ExAC and gnomAD projects have highlighted the utility of large databases of human genetic variation for genetic variant interpretation in the context of mutational processes, human disease and drug discovery. Despite the tremendous positive impact of such large genetic reference databases, they also pose new variant interpretation

challenges. Any predicted disease-causing variants in healthy individuals that are not correctly filtered out, for instance, can have confounding effects on genetics analyses, including skewing estimates of incomplete penetrance.

Our manuscript integrates the most comprehensive human tissue functional genomics dataset, the Genotype Tissue Expression project (GTEx), with the largest publicly available database of genetic variation, to provide a novel framework for variant interpretation that leverages the expression state of specific regions across tissues. We develop a metric, pext, that can be used for variant interpretation, functionally validate it using orthogonal measures, and show its use in both common and rare diseases. This represents an entirely novel use of the GTEx dataset for coding variant interpretation, and helps address the paradox of variants identified in public databases that should not be present in the general population.

We make the resulting combination of GTEx and gnomAD data publicly available via the gnomAD browser, thus making a key output of the GTEx dataset (regional expression) available for analysts and clinicians in the way that is most useful to them. We believe that the combination of these two powerful datasets itself provides insights into variant interpretation for both common and rare diseases, which will only be further amplified by pext being used by the research community to integrate the vast amount of functional genomic data into analyses of human genetic variation. We note that gnomAD is only one such database, and we expect our scores to be useful in the context of other large-scale sequencing projects, such as exom and whole genome sequencing in UK Biobank, TOPMED, discovEHR and others.

Technical note to the editor and reviewers

During the publication process, we independently identified a technical issue affecting a small proportion of genes. In the calculation of the pext score, when a gene is assigned low relative expression for coding transcripts, and high expression for noncoding transcripts, all variants in the gene can be assigned low pext scores, which can result in filtering all variants in the gene. Our analysis shows that a majority of non-coding isoforms that are assigned high expression values shows are retained intron transcripts, which we believe arises from misassignment of expression to these transcripts due to presence of pre mRNA in RNA-seq data. This is additionally confounded by the fact that many non-coding retained intron transcript share exons with protein coding isoforms, exacerbating the misassignment of high expression to such transcripts. In other words, assignment of higher expression to noncoding vs protein coding transcripts most likely represents an artifact of isoform quantification for most genes and complicates the isoform quantification of true protein coding transcripts.

Since the issue originates from the assignment of high expression to noncoding transcripts, and represents a biologically unlikely phenomena that doesn’t aid coding variant interpretation and is inherent in the isoform quantifications, we have removed 668 genes that are affected by this, representing 3.3% of all protein coding genes. We have regenerated all of our main and supplementary figures, and see no visible differences compared to the non-filtered analysis. While there is no overlap with the 665 genes and the haploinsufficient gene

list, 97 of the filtered genes are present in OMIM (representing 1.5% of the OMIM gene list) and 41 filtered genes are considered constrained (representing 1.4% of LOEUF < 0.35, or constrained, genes). As such, this exclusion has minimal impact on the global analyses described in the paper. However, users of the resource will need to be aware that pext scores are unavailable for a small fraction of genes, and we have made sure this caveat is described clearly in the paper (Methods section).

Reviewer #1:

In this work, Cummings and colleagues presented a new metric, pext, to quantify the variation of variant effects in the same gene due to alternative splicing. They showed that regions with lower pext scores are less likely to be conserved across species, are depleted with known disease variants, and are depleted with de novo mutations identified in probands with severe neurodevelopmental disorders. This strategy is interesting and has the potential to aid variant interpretation in medical genetic studies. Also, this manuscript is very well written. However, I feel this work would be a better fit for a more specialized journal.

1) I am concerned about how much additional information for variant prioritization we could gain from pext after controlling for other well-known predictive features. Figure 2B clearly shows that LOFTEE prediction and mean pext score are not perfectly correlated. However, I don't think this figure is direct evidence to support the authors' argument that "pext provides orthogonal information to conservation for variant interpretation" (see line 145 in the main text). Indeed, another possibility is that the predictive power of pext is merely due to its correlation with sequence conservation. To rule out this explanation, the authors should check whether the high pext bin is still enriched with known ClinVar pathogenic variants after controlling for LOFTEE flag and other conservation scores, such as phyloP and phastCons scores. If pext still has significant predictive power after controlling for these confounding factors, then we can probably say that pext provides orthogonal information to sequence conservation for variant interpretation.

We would like to initially note that that variants on regions with low sequence conservation are more likely to be filtered by pext, but highlight that pext also filters variants in highly conserved regions, highlighting the additional information provided by our method (displayed in Figure 2B and Supplementary Figure 5B) and showing that it is not captured by conservation alone. We also note that a majority of analyses utilizing LOFTEE focus on the filters (HC, LC) and not flags, evidenced by the use of LOFTEE HC variants in the gnomAD main paper (no flag filtering used for constraint calculations, Karczewski et al 2019. **doi:** https://doi.org/10.1101/531210), and several burden analysis papers (example doi:10.1038/s41586-019-1231-2). We finally note that while information on low sequence conservation is directly available for pLoF variants via LOFTEE, it does not annotate non-pLoF variants, and to our knowledge conservation alone is not widely used as a broad filter in exome analyses in rare and common diseases.

To address the reviewer’s concerns, and show that pext offers additional information after accounting for the LOFTEE flags, we have followed the reviewer’s guidance and evaluated the number of variants filtered in ClinVar and gnomAD, stratified by LOFTEE flags. We provide this in a new Supplementary Figure 7A, which shows that an overwhelming majority of the variants filtered from ClinVar across all gene categories - including haploinsufficient developmental delay genes, autosomal recessive disease-associated genes, and constrained genes (LOEUF < 0.35) - are LOFTEE high-confidence (HC), with no flags. Proportionally, pext allows filtering a majority of LOFTEE HC - phyloCSF weak flags from gnomAD (36/41, 31/49 and 1,840 / 2,642 of pLoF variants in haploinsufficient, autosomal recessive, and LOEUF genes, respectively). However, we also filter 115, 465, and 3,689 pLoF variants from these genes that are LOFTEE HC with no flags, demonstrating that our results are not simply driven by factors that are already captured with LOFTEE flags.

2) Similarly, the authors may investigate, after controlling for LOFTEE flag and other conservation scores as confounding factors, whether the high pext bin is still enriched with proband variants (Figure 5).

We have updated the manuscript with Supplementary Figure 7B, which represents the *de novo* analysis using LOFTEE HC pLoF variants with no flags (removing variants with any of the available flags which are phyloCSF weak, phylocCSFunlikely ORF, single exon, NAGNAG site, non-canonical splice site). We see no substantial impact on the conclusions presented and have added a comment on line 204 acknowledging this observation.

We note that this lack of impact is predictable given that of 2,161 *de novo* LOFTEE-HC pLoF variants in the dataset, the breakdown of those with LOFTEE flags are: 9 for NAGNAG site, 4 for non-canonical splice site, 1 phyloCSF unlikely, 46 phyloCSF weak, and 38 single exon, representing a total of 99 filtered variants (4.6% of variants). The point estimate changes are the following: For DD/ID, the high expression category for pLoF changes from 4.64 to 4.82, and the low expression category changes from 1.00 to 1.18; for ASD the high expression category changes from 2.11 to 2.17 and the low expression category changes from 0.80 to 0.78. Overall, the addition of the supplementary figure shows that the signals seen in the ClinVar gene list comparisons and the *de novo* variant analysis via pext bin stratification is not driven by factors already captured by any LOFTEE flag.

3) Many machine learning models, such as CADD and PolyPhen-2, have been developed to prioritize disease variants by integrating a large number of predictive features. If pext indeed provides orthogonal information for variant prioritization, the inclusion of pext as a new feature should significantly improve these machine learning models in the prediction of known disease variants. To test if this is the case, one could download the training data of a machine learning model, say CADD, add pext score as a new feature, and retrain the machine learning model. Then, the new model could be compared with the original model using standard ROC or precision-recall curves.

Completely remodeling the Polyphen-2 and CADD scoring systems falls outside the scope of our manuscript, especially given that we expect pext to be used as a complimentary variant annotation tool in combination with these and other *in silico* variant prioritization tools. However, this concern can be addressed using the mutability adjusted proportion of singletons (MAPS) metric, which provides a measure of deleteriousness of a class of variants, and evaluating the MAPS score within a PolyPhen-2 category with pext. To address the reviewer's concerns, we combined PolyPhen-2 'probably damaging' categorization of missense variants with pext expression bins (high, medium and low) and calculated MAPS on each variant class. This analysis is included in the analysis as Supplementary Figure 5D. If pext simply recapitulates PolyPhen-2, we would expect no difference between the high and low pext bins for probably damaging variants. However, we see that even filtering to probably damaging missense variants, pext expression bins add a gradient of deleteriousness (point estimates for probably damaging variants are : 0.155, 0.154, 0.11 for high, medium and low pext expression bins for highly constrained genes) showing that pext does not simply recapitulate PolyPhen-2, which explicitly considers conservation, and that the scores can be used in addition to PolyPhen-2 categorizations.

4) It is well known that alternatively spliced exons are more conserved than constitutively spliced exons (PMID: 12730695, 16157889, 16368777, and 16620375). These papers should be cited and discussed.

These citations conclude that alternatively spliced exons are less evolutionarily conserved than ones that are constitutively kept in the transcript, so we are assuming that this is what the reviewer meant to say (not the inverse). PMID 12730695 is the "first large-scale evidence for a potentially useful hypothesis that alternative splicing can play a unique part in evolution by increasing the rate of evolutionary change in specific exons" implying increased rate of evolution (and less conservation) in alternatively spliced exons; PMID 16157889 concludes "[Our] finding suggests that alternative splicing-accelerated evolution has produced adaptive functions that have been selected for during recent evolution" which again associates alternative splicing with more diversity, and thus less evolutionary conservation; and PMID 16368777 states "results indicate that ASEs have lower synonymous rates but higher nonsynonymous rates than CSEs. The elevated K_A/K_S ratio in ASEs implies that ASEs may have contributed more to protein diversity than CSEs during mammalian evolution." Finally, PMID 16620375 concludes: "results provide evidence of weaker purifying selection and/or stronger positive selection in alternative regions and thus one more confirmation of accelerated evolution in alternative regions." Therefore the citations are in agreement that constitutive exons (exons that are more likely to be kept in the mRNA transcript) have higher conservation, and with our observation that more conserved regions should be enriched for higher pext scores.

We acknowledge the relationship with conservation and expression in the manuscript page 4, paragraph 3: "Exons with patterns of multi-species conservation consistent with coding

regions have higher phyloCSF scores, and should exhibit detectable expression patterns, whereas regions with lower scores will be enriched for incorrect exon annotations, which are expected to have little evidence of expression in a population transcriptome dataset.” and use this as a functional validation tool for our method. However, we agree with the reviewer that more context is helpful here, and we have added in the suggested citations and the consideration that alternative splicing, which exhibits weaker conservation, should also lead to lower expression on page 4 paragraph 3.

5) What's the recommended pext cutoff for variant prioritization? If we use a cutoff of 0.1, only a small fraction of putatively benign mutations will be filtered out (Figure 5). Can we do a better job by tweaking the cutoff? Is there any principled way to choose it?

In this manuscript, we have binned pext scores in terms of low (mean pext across GTEx < 0.1), medium (0.1 ≤ x ≤ 0.9) and high (> 0.9) expression. In other words, in this manuscript pext allows for filtering low-expressed variants, which are more likely to capture annotation errors (as observed in the Supplementary Figures 1 and 6), and prioritizes regions with near-constitutive expression (exemplified in the higher rate ratios seen for *de novo* variants across a range of disorders and higher MAPS values). We acknowledge this bifurcation in line 249 in the manuscript and note that intermediate levels of pext are more difficult to interpret. Based on the reviewer’s comment, have added in Supplementary Figure 9, which shows the proportion of variants filtered from ClinVar and gnomAD across the set of 3 gene lists used in Figure 4. Here, instead of using categorical bins for average pext, we use the exact cutoff values for mean pext across GTEx tissues (SuppFig9 - top), maximum pext across GTEx tissues (Supp Fig9 - middle) and minimum pext across GTEx tissues (SuppFig9 -bottom). We note that as expected, as the pext cutoff values increases, more variants are filtered from both ClinVar and gnomAD. Here the choice of 0.1 as a cutoff for the gene list comparison seems effective, considering slightly tweaking the value to 0.2 or 0 does not have a large impact on the selective filtering of pLoF variants from gnomAD compared to GTEx. We have added text starting on line 252 to acknowledge that the chosen cutoffs will be a consideration for variant interpretation. Beyond this, we have thoroughly benchmarked pext at the chosen cutoffs in this manuscript, and recommend this, akin to the CADD suggesting a cutoff of 15 or 20, but allowing users to determine their own cutoffs for their specific analyses.

Referee #3 (Remarks to the Author):

The paper by Cummings et al. describe a method that uses transcriptomic data to help in assessing the functional impact of genetic variants. In short, the authors propose a measure, pext, which roughly corresponds, if I understood correctly, to the sum of the expression of all transcript isoforms including that variant over the total expression of the gene. The assumption is that variants with high pext score (i.e. in highly expressed transcripts) are likely to have larger impact than those with low score (i.e. in lowly expressed transcripts).

pext would be ideally computed on large number of samples across multiple tissues, but in principle can computed using any number of samples. The assess the pext metric, the authors use the GTEx data set to compute it for a set of putative LoF variants found in 61 manually curated haploinsuficient developmental genes. The authors show that for low pext scores, a larger proportion of likely incorrect LoF variants are filtered out than of high quality pathogenic variants. Finally, they show that the pext score can be efficiently used to prioritize causative variants in a number of conditions.

It looks to me that the approach developed by the authors is sensible. The method, while straightforward, appears to be effective, and it has been overall well benchmarked. There is a lot of manual curation involved through the entire paper, which adds interest to the paper as resource. Also important as a resource is the precomputed values for every variant in the human genome. The paper is, thus, mostly methodological and does not bring many novel biological insights. I believe, for instance, that the relation between levels expression and sequence conservation has been quite well documented, and previous work should actually be referred. The paper is overall clear and logically structured, and I do not have serious issues with it.

1. One of the issues that is less clear to me is that the 61 disease genes selected for evaluation are likely to be lethal during development (actually, this is the reason why they are selected). However, their expression is calculated in GTEx tissues that are all adults. That a transcript containing a pathogenic variant is not expressed in adult tissues, it does not mean that it is not expressed during development. Therefore, in my opinion, it would be much more informative to compute the pext score on developmental transcriptomic samples than in adult ones. Actually the authors allude to this in Figure 6D. Is there a reason why they do not employ transcriptomic developmental data (maybe combined with GTEx) through all the analysis?

We thank the reviewer for the comment. The reviewer is correct that at the initial submission of the manuscript, we had provided precomputed pext values based on the Human Brain Developmental Resource dataset, of which we had described the processing and annotation in the Methods section (line 533). In the initial submission we did not perform analyses on the HBDR fetal RNA-seq dataset given that GTEx is much richer in scope, with approximately 11,000 samples compared to the 600 in HBDR, and because we did not want to suggest a comparison in the quality of the datasets. One analysis showing the importance of the fetal isoform dataset to resolve the expression status of a conserved region was shown in Supplementary Figure 6D.

Based on the reviewer’s comment, we have evaluated the use of the HBDR dataset for filtering variants in haploinsufficient genes from gnomAD and ClinVar, and added in Supplementary Figure 10 showing the varying effects of individual fetal tissues. Here, we show the proportion of variants filtered from gnomAD and ClinVar in these genes with

varying cutoffs of pext, per fetal tissue. We note that the fetal isoform dataset does not provide any more selective filtering than GTEx, likely due to the fact that pext here is mostly capturing annotation errors and fetal RNA-seq does not provide additional information than the GTEx dataset.

2. The rationale for the authors to use transcript isoform quantification tools, rather than simply read coverage of variants, is that the tools would hopefully deal with the technical biases implicit in short read RNASeq (such as 3' bias). This may be the case, but also, as the authors acknowledge, there are also many issues involved in the deconvolution of RNASeq reads to produce transcript quantifications. It is not at all clear that one compensates for the other. It maybe useful to compute the pext score based on read coverage and compare the results when computed based on transcript quantifications. If the results are comparable, there maybe a rationale to prefer read coverage, since it implies less data processing.

The removal of confounding bias caused by 3' bias and other biases with isoform quantification tools is quite well-established in the transcriptome sequencing literature (PMIDs 22285831, 21816040, 28263959, 21771300 among others). We also display this in the manuscript in Supplementary Figure 2. Showing an example of *LAMA2* we note that while exon 5 and 55 are annotated as belonging on a single transcript together, the median read coverage in GTEx for these exons is 0.8 and 3.25 respectively. This represents a 4x difference in read pileup, which is a known effect caused by 3' bias. Therefore using read coverage alone would no doubt result in differential filtering of variants based on their proximity to the 3'. This effect is also known to be augmented for longer isoforms, and therefore we expect biases in genes with a combination of long and short isoforms that are difficult to resolve. This is precisely the goal of isoform quantification tools, which statistically account for 3' bias, as well as other biases. While we acknowledge in our manuscript that statistical assignment of isoform expression based on short read data is imperfect (line 100) we believe the confounding effect of 3' bias completely prevents the use of simple read pileup for variant interpretation.

Logistically, the exon expression counts provided by GTEx would not be sufficient to annotate pext given that in many cases, isoform contain parts of composite exons (for an example see transcripts B and C in Figure 2A). Therefore, recomputing pext based on coverage alone would require processing all of GTEx for pileup to validate an already well-known result. We however note that if this processing existed, the software provided through the manuscript allows for efficient pext quantification, which we emphasize will only improve for example with the large scale application of long read RNA-seq data, minimizing the biases set forward by statistical isoform quantification. In summary, the benefit of statistical quantification over simple read pileup is evident for RNA-seq data, and we do not believe this will provide a substantial improvement of the manuscript.

3. Related with the point above, the authors used an arbitrary metric, the average transcript expression across tissue samples, but, as they also acknowledge, other metrics could be used. They explicitly mention the minimum expression (but it could also be the maximum). Since the computation of the pext score is quite efficient and inexpensive, it would really be useful to provide a comparative evaluation of the different such metrics. In general, the three comments above all refer to the investigation of the impact on the calculation of pext score of a number of factors: transcriptome dataset, method to compute the score and statistic to aggregate over samples. I think these analyses would provide a more general framework to understand the relevance of the method proposed.

We thank the reviewer for the comment. We have now incorporated two additional Supplementary Figures based on the comment that it would be useful to evaluate differing cutoff and RNA-seq datasets for their impact on the analysis. We note that this is in addition to Supplementary Figure 11 which shows the analyses in the manuscript via two different isoform quantification tools, salmon and RSEM, to show the results are robust to the specific tool utilized. In Supplementary Figure 9 we evaluate the proportion of variants filtered across varying gene lists using the average, maximum and minimum pext across tissues. In this figure we also use the cutoff values themselves instead of categorical binning of the pext scores. We find that general trends of selective filtering of variants from gnomAD over ClinVar with pext is consistent across the varying methods for summarizing pext. As the cutoff increases, so does the number filtered variants, but neither of the other summarizing approaches (minimum or maximum) or the specific cutoff used improve the selective filtering, showing that for this analysis, the summarizing method and cutoff used in the manuscript are representative of the effect of pext. In Supplementary Figure 10, we evaluate a fetal brain RNA-seq dataset from the Human Brain Developmental resource. Here we again do not see a significant improvement in selective filtering of variants from ClinVar versus gnomAD, suggesting the method and analyses presented in the manuscript are consistent with alternative summarizing approaches.

4. There has been recently a quite intense debate on the relevance of alternative splicing in generating protein diversity, with claims that this relevance has been vastly overestimated (DOI:10.1016/j.tibs.2016.08.008) subsequently disputed (DOI:https://doi.org/10.1016/j.tibs.2017.04.001). It looks to me that the paper by Cummings et al. does support the relevance of alternative splicing in establishing phenotypes. Maybe the authors want to discuss on this.

This is an excellent point - thank you. We would like to note that we touch on the role of alternative splicing in variant interpretation in our introduction, calling into attention for example the effect of isoform diversity in genes affecting cardiomyopathy (lines 61 - 68). Due to the nature of the binning of pext scores, we emphasize the role of the metric to filter out annotation errors and to prioritize variants that are near-constitutively expressed. While we believe pext will be useful to delineate the effect of more nuanced alternative splicing patterns across tissues on phenotypes, we do not capture this with global analyses in our

manuscript. We expect such effects to emerge with more directed analyses by individuals with domain knowledge of a gene or phenotype of interest, as we acknowledge in our discussion, lines 249 - 256. Based on the reviewers comment, we have added the suggested citations in the discussion on line 251, acknowledging that such directed analyses of intermediate pext scores for genes will help elucidate the effect of alternative splicing on phenotype diversity.

Minor points :

1. To me the only difference between Figure 3C and 5D is the inclusion of the missense variants in the latter, but other than that they are identical. I am not sure that the two figures are needed.

We agree with the reviewer. The initial Supplementary 5D was meant to show the effect of pext stratification on missense variants, which follow a comparable pattern to pLoF variants. Therefore initially Supplementary Figure 5D was similar to figure 3C, with missense variants included. Following the reviewer's suggestion, we have now replaced Supplementary Figure 5D with an updated version which shows only missense variants, in the probably damaging PolyPhen-2 category.

All other language and spelling suggestions by the reviewer have also been updated. We thank the reviewer for their careful reading of the manuscript.

Referee #4 (Remarks to the Author):

Cummings et al. develop a novel annotation metric (i.e., pext) that summarizes exon expression information across transcripts and describe a new annotation tool that uses this information to facilitate variant interpretation. They demonstrate that development of an expression-based annotation metric addresses a common confounder (i.e., transcript errors) of variant annotation and that the tools is fairly effective at flagging falsely annotated pLOF variants. More importantly, they show that expression-based annotation identifies functionally important regions of the genome, provides orthogonal information to conservation estimates, and that it can be used to facilitate interpretation of variant with large, deleterious effects that underlie Mendelian conditions. The manuscript is clear and well-written, the conclusions well-supported, and the applicability nicely illustrated. I have no major concerns about the strategy used to develop the annotation metric, the use cases presented, or it's potential usefulness to the field. All of this withstanding, it's not clear to me that this metric or the tool developed represents such a major advance as to warrant publication in Nature. Indeed, the manuscript seems like other reports of new annotators, tools, etc. that are commonly published in discipline-specific human genetics journals.

We thank the reviewer for their comments on the efficacy of the method, the clarity of the manuscript and the demonstration of broad usage. We note the reviewer did not provide any technical concerns, and believe we are able to address the broader implications of our annotation tool in the letter to the editor and reviewers.

Reviewer Reports on the First Revision:

Referee # 1 The authors have addressed my technical concerns properly. However, I still feel this manuscript may not be a good fit for publication in Nature. This manuscript provides limited biological insights even though pext seems to be a useful tool for variant prioritization. I would recommend publishing this paper in a more specialized journal in the Nature family.

Referee # 3 The authors have addressed most of the issues (rather comments/suggestions) that I had after reading the manuscript for the first time. As I had already said, the manuscript is technically sound and the authors show that the method can be useful in assessing the impact of putative loss of function (pLoF) variants. One of the results of the paper is that pLoF variants in weakly expressed regions have effect sizes similar to those of synonymous variants. Two things that are unclear to me is how the method behaves in the case of genes with very tissue specific expression—if the tissue is not included in the set of tissues for which the ptex score is computed. Second ptex scores are computed from the GTEx samples. As additional RNASeq samples become available (for instance, from single cell projects) is there a way in which the ptex scores could be updated from the GTEx computed scores.

Author Rebuttals to First Revision:

Reviewer #3:

The authors have addressed most of the issues (rather comments/suggestions) that I had after reading the manuscript for the first time. As I had already said, the manuscript is technically sound and the authors show that the method can be useful in assessing the impact of putative loss of function (pLoF) variants. One of the results of the paper is that pLoF variants in weakly expressed regions have effect sizes similar to those of synonymous variants.

1. Two things that are unclear to me is how the method behaves in the case of genes with very tissue specific expression—if the tissue is not included in the set of tissues for which the ptex score is computed.

We thank the author for the comment. If a gene is not expressed in a tissue or group of interest it is not considered in our analyses of the tissue. The pext calculation involves normalizing the expression value for a given variant annotation by the total expression of the gene on which the variant occurs. If the gene expression value (and thus the denominator) in a given tissue is 0, the pext value will not be available for that tissue (it will receive an NA value). This is described in the Methods section of the manuscript.

Depending on analysis goal, variants with an NA pext value can be included into the final variant set, or filtered. One can imagine two differing analyst decisions on utilizing NA values. In one case, variants for which the pext value is NA can be included in the analysis, since there is not enough information to filter this variant. Alternatively, if the analyst is interested in looking at variants in a brain phenotype and is interested in brain tissues, they may filter out variants with an NA pext value, given that an NA pext would mean the gene is not expressed in a brain tissue, and may not be as biologically relevant.

It is the case that biologically critical gene regions may not be expressed at all in any of the adult tissues represented in GTEx. Users of the pext score need to bear this in mind in their interpretation, particularly for phenotypes where there is a high likelihood that causal regions may be expressed in a specific developmental window or in a specific tissue that is not captured by GTEx. We note, however that while this may affect specific genes with very confined expression, it does not have an impact on the global analyses performed in the manuscript (evidenced by the functional validation of pext, shown in Figure 3).

In summary, in the cases the reviewer has flagged, pext will not be available, and these are clearly denoted in the gnomAD browser (an example of a gene not expressed in any GTEx tissue and the pext annotation which clearly states “gene is not expressed in GTEx tissues can be found here
: https://gnomad.broadinstitute.org/gene/ENSG00000186092?dataset=gnomad_r2_1)

Finally, we note the point brought up by the author is a motivation behid our writing the pext algorithm to be generically applicable to any isoform expression dataset, to encourage the use of multiple different resources, which we underline with a region expressed only in development in Supplementary Figure 6D.

2. Second ptex scores are computed from the GTEx samples. As additional RNASeq samples become available (for instance, from single cell projects) is there a way in which the ptex scores could be updated from the GTEx computed scores

The transcript expression annotation pipeline is written to be generically applicable to annotate any variant dataset with any isoform expression data and we encourage using the pipeline with other datasets. To demonstrate the application of other RNA-seq datasets in the manuscript we provide annotations of gnomAD and ClinVar variants haploinsufficient developmental delay genes with isoform expression data from the Human Brain Developmental Resource which includes 558 samples from varying brain subregions across developmental time points, which we analyze in Supplementary Figure 10. In addition, in our github repository we lay out the file format requirements for a variant dataset (which is only

the presence of columns for chromosome, position, reference and alterative alleles) and the isoform expression matrix (which must have transcript id and gene id columns) to make the application of our pipeline to other datasets as straightforward as possible.
